# Iminosugar-Based Nicotinamide Phosphoribosyltransferase (NAMPT) Inhibitors as Potential Anti-Pancreatic Cancer Agents

**DOI:** 10.3390/pharmaceutics15051472

**Published:** 2023-05-11

**Authors:** Irene Conforti, Andrea Benzi, Irene Caffa, Santina Bruzzone, Alessio Nencioni, Alberto Marra

**Affiliations:** 1 Institut des Biomolécules Max Mousseron (IBMM, UMR 5247), Université de Montpellier, Pôle Chimie Balard Recherche, 1919 Route de Mende, CEDEX 5, 34293 Montpellier, France; 2 Dipartimento di Medicina Sperimentale-DIMES, Scuola di Scienze Mediche e Farmaceutiche, Università degli Studi di Genova, Viale Benedetto XV 1, 16132 Genova, Italy; 3 Dipartimento di Medicina Interna e Specialità Mediche-DIMI, Università degli Studi di Genova, Viale Benedetto XV 6, 16132 Genova, Italy; 4 IRCCS, Ospedale Policlinico San Martino, 16132 Genova, Italy

**Keywords:** *C*-iminoglycosides, glycosyltransferase inhibition, NAD, organocatalysis

## Abstract

The nicotinamide phosphoribosyltransferase (NAMPT) is considered a very promising therapeutic target because it is overexpressed in pancreatic cancer. Although many inhibitors have been prepared and tested, clinical trials have shown that NAMPT inhibition may result in severe haematological toxicity. Therefore, the development of conceptually new inhibitors is an important and challenging task. We synthesized ten β-d-iminoribofuranosides bearing various heterocycle-based chains carbon-linked to the anomeric position starting from non-carbohydrate derivatives. They were then submitted to NAMPT inhibition assays, as well as to pancreatic tumor cells viability and intracellular NAD^+^ depletion evaluation. The biological activity of the compounds was compared to that of the corresponding analogues lacking the carbohydrate unit to assess, for the first time, the contribution of the iminosugar moiety to the properties of these potential antitumor agents.

## 1. Introduction

According to the World Health Organization (WHO), pancreatic cancer is the 12th most common cancer worldwide (both sexes, all ages) and has a very high ratio of mortality to incidence, resulting in a low chance of survival. Unfortunately, pancreatic cancer is often diagnosed at an advanced stage, when it cannot be removed by surgery, due to the lack of specific screening tests and the absence of evident symptoms in the early stages of the disease.

### 1.1. The Role of NAD Co-Enzyme in Cancer Cells

It is well-known that the unique energy metabolism occurring in cancer cells allows their rapid proliferation. In non-malignant cells, under normal aerobic conditions, d-glucose undergoes glycolysis to produce pyruvate, which is then converted to acetyl-CoA that enters the citric acid cycle in the mitochondria to give 32 molecules of ATP via oxidative phosphorylation. On the other side, in malignant cells, even in aerobic conditions, anaerobic glycolysis predominates while oxidative phosphorylation is reduced, leading to the reduction of pyruvate to lactate, thus producing 2 molecules of ATP from one molecule of d-glucose. This reprogrammed metabolic process is called the Warburg effect [1]. Cancer cells exploit the benefits of the anaerobic glycolysis, i.e., faster rate of ATP production and reduced generation of reactive oxygen species (ROS) produced during respiration, instead of taking advantage of a more efficient ATP-generation. However, due to the lower amount of ATP available in cancer cells, the Warburg effect is closely related to the higher production of nicotinamide adenine dinucleotide (NAD), crucial to support rapid growth and proliferation. Since NAD is a co-enzyme that mediates redox reactions in many metabolic pathways, including glycolysis, its continuous replenishment promotes the proliferation and survival of fast-dividing cancer cells [2].

**Scheme 1:**
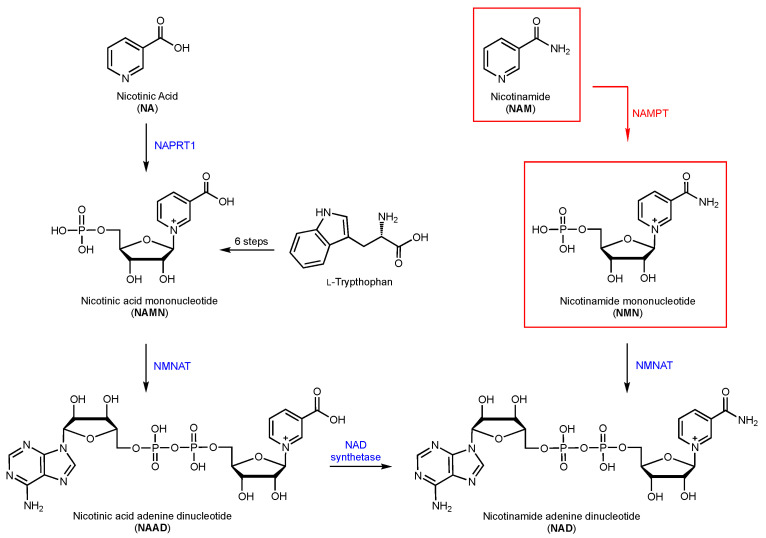
Biosynthesis of nicotinamide adenine dinucleotide (NAD).

Although living organisms can synthesize NAD (Scheme 1) from tryptophan, nicotinic acid and nicotinamide (NAM), in the case of mammals NAD is produced starting from the latter compound, thanks to a couple of key enzymes: the nicotinamide phosphoribosyltransferase (NAMPT) and the nicotinamide mononucleotide adenylyltransferase (NMNAT) [2]. NAMPT, which can be intracellular and extracellular [3], is a glycosyltransferase (GT), i.e., an enzyme catalysing the regio- and stereospecific transfer of a sugar moiety from a sugar donor bearing a leaving group (phosphate or nucleotide diphosphate) at the anomeric position onto saccharide and non-saccharide acceptors. The glycosyltransferases catalyze the synthesis of the glycosides with complete inversion (inverting GTs), as in the case of NAMPT, or retention (retaining GTs) of the sugar donor anomeric configuration. Being overexpressed in several types of malignant tumors, including pancreatic cancer, NAMPT was considered a very promising therapeutic target. A large number of selective inhibitors have been developed and tested in vitro and in vivo, however, since NAMPT is also essential for health cells, clinical trials have shown that its inhibition may trigger severe haematological toxicity.

### 1.2. The Structure of the NAMPT Enzyme

**Figure 1:**
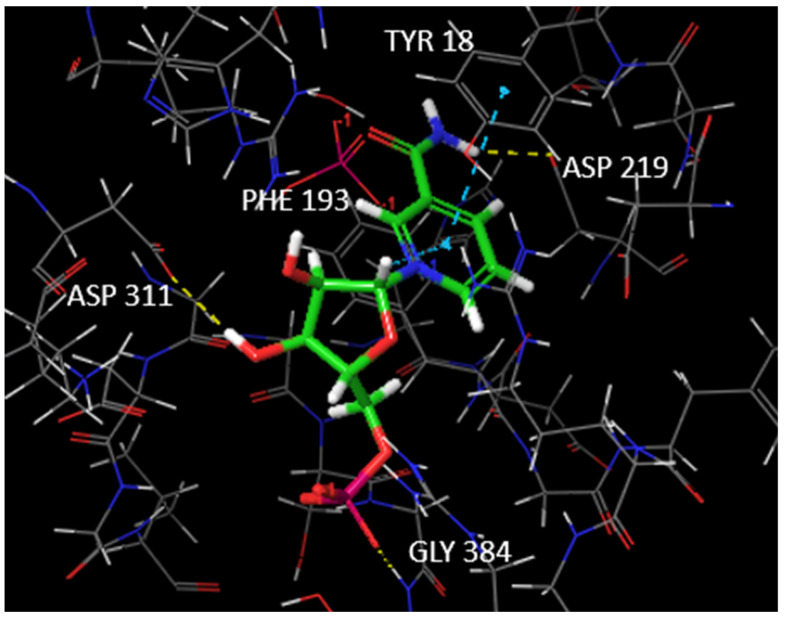
Nicotinamide mononucleotide (NMN) complexed at the active site of NAMPT.

The crystal structure of human NAMPT was published in 2006 [4], when nicotinamide mononucleotide (NMN) was co-crystallized to study its binding mode (Figure 1). The co-crystal of NAMPT proved that the enzyme is a dimer, where the two monomers are arranged head to tail, with domain A in one monomer contacting domain B in the other monomer. NMN binds near the top of the central β-sheet in domain B, but both domains B and A have several residues playing a crucial role in recognizing NMN. The nicotinamide moiety of NMN is sandwiched between the side chain of Phe193 of one monomer and a residue of Tyr18 of the other monomer. The nitrogen of the amide is hydrogen-bonded to the side chain of Asp219. These behaviours suggest that NAMPT is only active in its dimeric form. Moreover, the activity of NAMPT depends on the phosphorylation of the His 247 that strongly increases its affinity for the phosphoribosyl pyrophosphate (PRPP). In the same way, the complex of the phosphorylated enzyme with PRPP displays a 160,000-fold increase of affinity for nicotinamide (NAM) and a 1000-fold efficiency in the production of nicotinamide mononucleotide (NMN) [5].

### 1.3. The Interactions of NAMPT with Its Inhibitors

**Figure 2:**
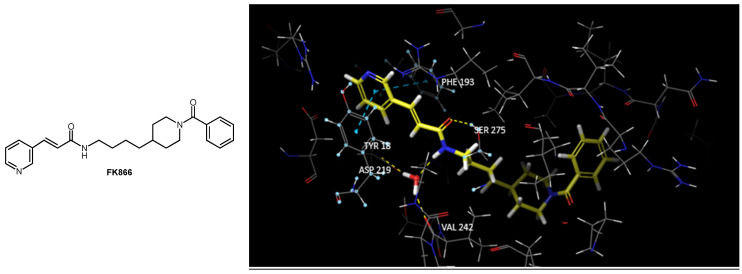
Binding mode of **FK866** complexed at the active site of NAMPT.

In the same 2006 article [4], it was also described the co-crystallization of the enzyme with **FK866** (Figure 2), a strong, tight-binding competitive inhibitor (IC_50_ = 3.3 nM) whose activity is very likely due to a very slow rate of dissociation from the enzyme [4]. Therefore, **FK866** functions as an irreversible NAMPT inhibitor during the kinetic assays [4]. The X-ray structure revealed that the pyridine moiety of **FK866**, analogously to the pyridine unit of the NMN, is sandwiched between Phe193 and Tyr18 of the active site, making a π–π stacking interaction between the aromatic rings of the two amino acids. The carbonyl oxygen atom of acrylamide is hydrogen-bonded to the hydroxyl of Ser275, while the nitrogen of amide is bonded to a water molecule that occupies the binding side and is stabilized by interactions with Asp219 and Val242. The alkyl chain, a linker between the inner and the external parts of the active site of NAMPT, does not show any interactions in the hydrophobic tunnel. The solvent-exposed part of the molecule, i.e., the benzoylpiperidine moiety, can be considered as a tail group that anchors the molecule and could increase the affinity toward the enzyme.

**Figure 3:**
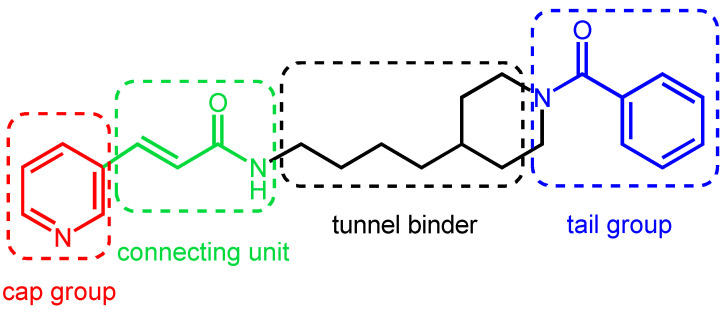
Pharmacophore model of NAMPT inhibitors based on the structure of **FK866**.

Then, other crystal structures of human NAMPT in complex with different inhibitors were obtained, suggesting a common pharmacophore model that could have the same or even more convenient interactions with the active site of NAMPT (Figure 3).

This model contains a tail group that is important for establishing interactions in the solvent-exposed region of the enzyme, and a hydrophobic tunnel binder where the alkyl chain could be replaced by a variety of spacer groups, such as benzene rings or hexyl, heptyl and octyl chains. The spacer is bounded to a connecting unit, e.g., an amide, which acts as a hydrogen-bond acceptor. However, due to the long and narrow binding pocket of NAMPT, modification of **FK866** appears to be mainly limited to the end portions. The cap group, mimicking the NAM unit, is usually a pyridine or other nitrogenated heterocycles.

### 1.4. The Controversial Role of the Pyridine Moiety

Over the past 20 years, many potential inhibitors carrying a pyridine ring have been prepared and assayed. Interestingly, in the publication [4] describing the crystal structure of human NAMPT, no interactions between the nitrogen of the pyridine and any residues of the binding site were reported in order to justify the presence of a nitrogen-contain heterocycle in the inner site. Later on, several explanations were provided and some studies confirmed the presence of a hydrogen bond between the pyridine nitrogen, and a residue of Asp 219 [6] or the hydroxyl group of Tyr18 [7]. In 2014, it was demonstrated that the nitrogen atom can also be phosphoribosylated after bonding to the enzyme, thus enhancing the in vivo antitumor efficacy [8]. However, this evidence was then undermined by the discovery of potent inhibitors that did not contain a pyridine moiety [9].

However, in vitro metabolic studies have shown that pyridine-based NAMPT inhibitors suffered from microsomal oxidation leading to the *N*-oxide derivative [10], which was much less active in vivo than the reduced form. The presence of a pyridine ring might also be problematic due to the ability of the nitrogen of pyridine to chelate the iron (II) atom of the heme moiety in several cytochromes [11]. These findings paved the way to the design of inhibitors lacking the pyridine ring. Since 2015, some new inhibitors carrying various heterocycles as cap groups (benzothiophene, thiophene, pyrazole, isoindoline) showed a promising activity both in vitro and in vivo [7,12].

### 1.5. Mimics of the NAMPT Transition State as New Inhibitors

**Figure 4:**
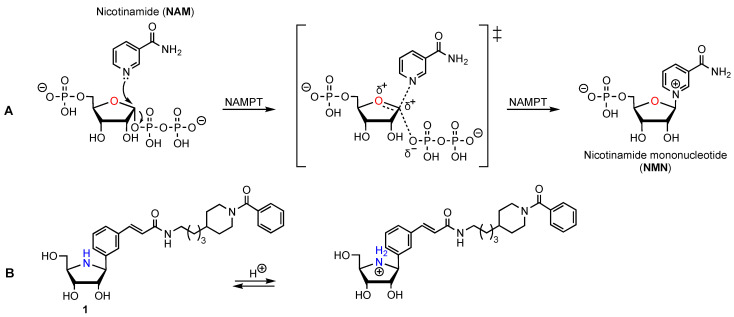
(**A**) Mechanism of the NAMPT-catalyzed glycosylation of nicotinamide (NAM) to give the nicotinamide mononucleotide (NMN) by S_N_2 reaction, involving a positively charged ribofuranose moiety in the transition state. (**B**) Structure of the potential NAMPT inhibitor **1** and its protonated form mimicking the transition state of the glycosylation.

In 2012, the conceptually new NAMPT inhibitor **1** was synthetized by Vogel and co-workers [13] in the aim of mimicking the transition state formed in the glycosylation process catalysed by NAMPT (Figure 4). The compound **1** featured an iminofuranoside unit anomerically *C*-linked to a moiety closely related to **FK866**, the pyridine ring being replaced by a phenyl ring. Since the basic nitrogen atom of the d-*ribo*-configured iminosugar unit is protonated at a physiological pH, it mimicks the partial positive charge located onto the oxygen atom of the d-ribose moiety in the glycosylation transition state. Furthermore, due to the known, poor solubility of the NAMPT inhibitors in water, the conjugation with a highly hydrophilic structure, such as an iminosugar, should also improve the pharmacokinetics properties. Unfortunately, the biological assays of **1** have never been reported in the literature and, in our hands, its synthesis could not be replicated. Therefore, we could not test the molecule as a NAMPT inhibitor and anticancer agent.

## 2. Materials and Method

The reactions were monitored by TLC on silica gel 60 F_254_ with detection by charring with *p*-anisaldehyde, KMnO_4_, ninhydrin or with reagent [(NH_4_)_6_MoO_4_, Ce(SO_4_)_2_, H_2_SO_4_, H_2_O]. Purification by column chromatography was carried out using either hand-packed glass columns (silica gel, 40–60 μm) or Puriflash XS520 Plus Interchim system with prepacked cartridges. Optical rotations were measured at 20 ± 2 °C in the stated solvent; [α]_D_ values are given in deg mL g^−1^ dm^−1^. ^1^H NMR (600 MHz) and ^13^C NMR (150 MHz) spectra (Bruker Avance 600, Vienna, Austria) were recorded in the stated solvent at room temperature unless otherwise specified. All the assignments were confirmed by 2D spectra (COSY and HSCQ). High-resolution mass spectrometry (Waters Micromass Q-TOF, Milford, MA, USA) analyses were carried out at the Laboratoire de Mesures Physiques, University of Montpellier. The purity of representative final compounds (>97%) was assessed by HPLC (Thermo Scientific, UltiMate3000, Waltham, MA, USA) on C18 reversed-phase column (Thermo Scientific, Hypersil GOLD aQ, 2.1 × 50 mm, 1.9 µm). Elution was performed using a binary gradient (solvent A: H_2_O + 0.1% TFA; solvent B: CH_3_CN + 0.1% TFA). The samples were eluted with a linear gradient from 95% A and 5% B to 0% A and 100% B, between 15 and 25 min. All chromatograms were registered at 254 nm wavelength. Preparative and semi-preparative HPLC were performed on a VWR instrument equipped with a LaPrep P110 pump and a LaPrep P314 Dual absorbance detector. A C18 reversed-phase column (Waters, XSelect CSH C18 OBD, 30 × 250 mm, 5 µm) was used for preparative HPLC and a C18 reversed-phase column (Macherey-Nagel, Nucleodur C18 HTec, 21 × 250 mm, 7 µm, Dueren, Germany) was used for semi-preparative HPLC. Elution was performed using a binary gradient (solvent A: H_2_O + 0.1% TFA; solvent B: CH_3_CN + 0.1% TFA).

**Computer-aided Drug Design** (**CADD**). All computational studies were performed using Maestro 13.3.121 (for academic use only). The chemical structure of iminosugar-containing compounds was prepared using Ligprep interface in Schrödinger with an OPLS3 force field at pH 7 ± 1, using Epik. The protein used for the docking study, human NAMPT in complex with **FK866**, was selected from PDB databank (PDB code: 2GVJ). The crystallized 3D structure was prepared with “protein preparation wizard” at a pH of 7 ± 1. In addition, the water molecules were removed from the protein during the preparation process, except for the water molecule described to be involved in the binding with the connecting unit. The receptor grid, i.e., the area of interaction between the protein and the ligand, was produced using the receptor grid generation tool, which identifies the area around the active site in terms of “Centroid of Workspace ligand”. The molecular-docking calculation was performed using the XP (Extra Precision) Glide mode, with ligands as flexible.

**NAMPT inhibition**. Ten ng of recombinant human NAMPT protein (#ab198090, Abcam, Cambridge, UK) were incubated in 40 μL reaction buffer (0.4 mM PRPP, 2 mM ATP, 0.02% BSA, 2 mM DTT, 12 mM MgCl_2_ and 50 mM Tris-HCl) in Eppendorf tubes, in the presence or absence of the different compounds. After a 5 min incubation at 37 °C, 9.0 μL of NAM (0.2 mM final concentration) were added and the reaction was stopped after 15 min, by heating samples at 95 °C for 1 min. Samples were then cooled to 0 °C, and NMN was detected by adding 20 μL of 20% acetophenone and 20 μL KOH (2M) into each tube. The mixture was vortexed and kept at 0 °C for 3 min; 90 μL of 88% formic acid was then added and the tubes were incubated at 37 °C for 10 min. Finally, 100 μL of the mixture was transferred into a flat-bottom 96-well plate and the fluorescence (excitation 382 nm, emission 445 nm) was measured using a CLARIOstar^®^Plus (BMG Labtech).

**Cell viability assay**. MiaPaCa-2 cells (2 × 10^3^/well) were plated in 96−well plates and left to adhere overnight. After 24 h the cells were treated with the inhibitors. Viability was determined after 72 h. The culture plates were fixed with cold 3% trichloroacetic acid at 4 °C for 30 min, washed with cold water and dried overnight. Finally, the plates were stained with 0.4% sulforodhamine B (SRB) in 1% acetic acid, washed four times with 1% acetic acid to remove unbound dye, dried overnight and then the stain was extracted with 10 mM Tris Base and the absorbance was read at 560 nm.

**Measurement of intracellular NAD^+^ levels**. MiaPaCa-2 cells were plated at a density of 6.5 × 10^4^ cells/well in 12−well plates. After 24 h, cells were treated (or not) with the different compounds and cultured for a further 24 h. Cells were harvested and lysed in 0.1 mL 0.6 M perchloric acid. Intracellular NAD^+^ levels were determined with a cycling enzymatic assay, as previously reported [14].

## 3. Results and Discussion

### 3.1. Docking Studies

**Figure 5:**
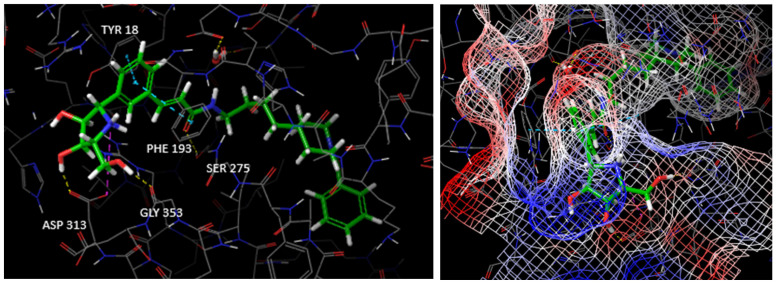
(**Left**) Interactions of **1** in the binding site of NAMPT. (**Right**) Electrostatic surface potentials of the binding site are in red and blue for negative and positive charges, respectively, whereas white represents neutral residues.

Despite the absence of biological results, we performed a docking analysis of **1** (see Figure 4) to evaluate its ability to occupy the active site of the enzyme (Figure 5). The iminofuranoside unit forms two hydrogen bonds between two hydroxyl groups and the Asp313 and Gly353 residues, and a salt bridge between the protonated nitrogen and Asp313. In addition, it displays all the other interactions featured by **FK866**, except for the hydrogen bond between the amide nitrogen atom and the water molecule. Moreover, the iminofuranoside moiety fits perfectly in the inner hydrophilic task of the binding site. The docking score revealed that **1** (−10.818 kcal/mol) was better suited for the binding site than **FK866** (−8.059 kcal/mol).

**Scheme 2:**
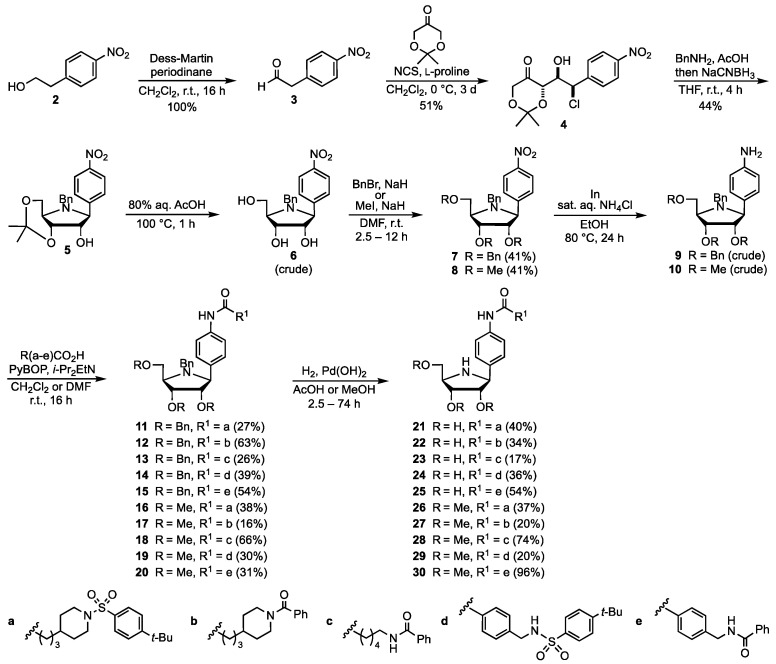
Synthesis of free-OH and *O*-methylated *C*-iminofuranosides.

**Figure 6:**
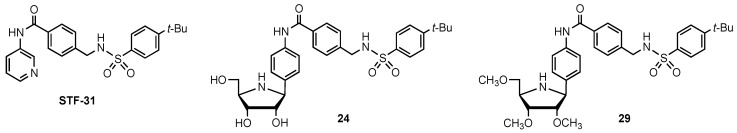
Structures of the NAMPT inhibitors **STF-31**, **24** and **29** (see Scheme 2 for their synthesis).

These promising results encouraged us to further pursuit the synthesis of iminosugar-based NAMPT inhibitors. First, we performed docking studies of newly designed molecules resulting from the assembly of known NAMPT inhibitors, or their analogues following the same pharmacophore model, with a *C*-iminofuranoside moiety. As an example, we describe here the docking behaviors of **24** (Figure 6, see Scheme 2 in Section 3.2 for its synthesis), the iminosugar-linked derivative of the pyridine-based NAMPT inhibitor **STF-31** (Figure 6).

**Figure 7:**
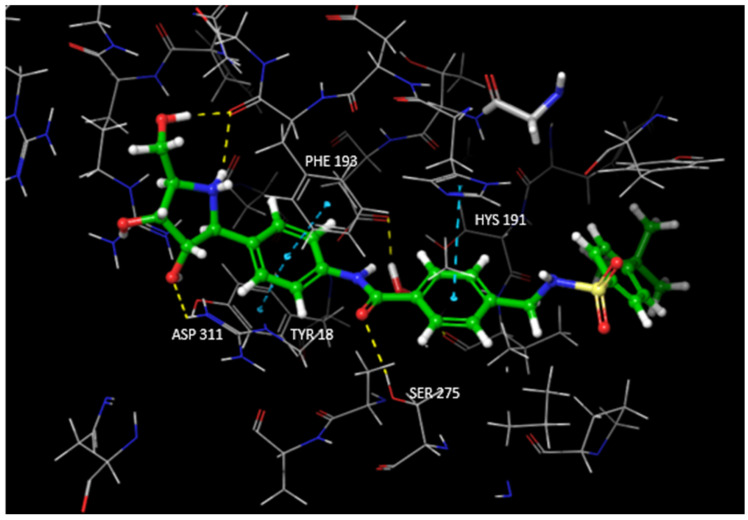
Docking model of the interaction between **24** and the NAMPT target. Compound **24** establishes interactions with key residues at the ligand binding site (LBD) of the enzyme: hydrogen bonds with Phe193, Arg 311, Ser 275 (represented by dotted yellow lines) and π–π stacking between Tyr18 and Phe193, His 191 (represented by dotted light blue lines).

We compared the binding pose of **1** to that of **24** and found that the *C*-iminofuranoside unit of **24** and **1** occupies the same site, but their interactions were different (Figure 7). The iminosugar was able to bind effectively with residues of Phe193 and Arg 311, forming hydrogen bonds. Moreover, inside the hydrophobic tunnel, the central benzene ring of **24** was bound via a π–π stacking interaction with a His 191 residue. It was also found that other interactions such as the hydrogen bond with the carbonyl oxygen atom of the amide, and the π–π stacking between Phe193 and Tyr18 were the same observed for **FK866** and other known inhibitors. For these reasons, the binding affinity of **24** was improved compared to **FK866**, as proved by a docking score of −10.428 kcal/mol, a value close to that observed for **1**.

**Figure 8:**
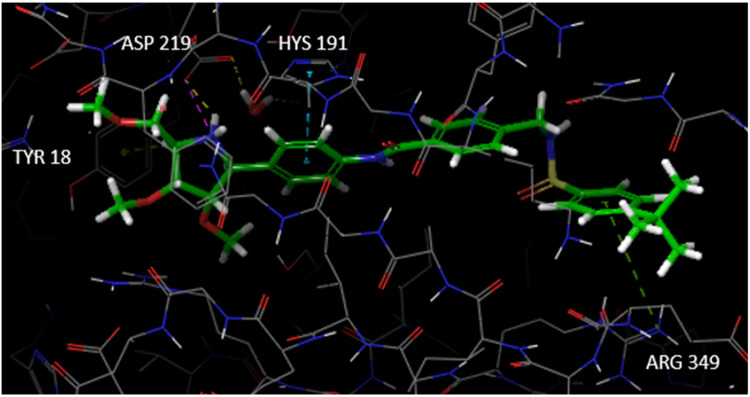
Docking model of the interaction between **29** and the NAMPT target. Compound **29** forms hydrogen bonds with Asp 219 (dotted yellow lines), π–π stacking with Hys191 (dotted light blue lines), salt bridge with Asp 219 (dotted pink lines), π-cation interactions with Tyr 18 and Arg 349 (dotted green lines).

Then, we decided to investigate the biological properties of less polar but more metabolically stable iminosugar-based NAMPT inhibitors. To this aim, we envisaged the preparation of **29** (Figure 6, see Scheme 2 in Section 3.2 for its synthesis), the analogue of **24** whose hydroxyl functions were converted into the corresponding methyl ether groups (see Figure 6). In this manner, the esterification and/or oxidation reactions of the iminosugar alcohols that may occur in vivo should be greatly reduced or even suppressed. The docking study revealed that the iminosugar moiety of **29** forms a salt bridge with a residue of Asp219, a hydrogen bond and a π-cation interaction (Figure 8). In addition, the benzene ring located at the anomeric position is involved in a π–π stacking with Hys191, whereas the other benzene ring has a π-cation interaction with Arg349. It is worth noting that **29**, unlike **24**, does not fit deep inside the pocket; nevertheless, the above-mentioned interactions lead to a rather good docking score (−6.940 kcal/mol).

The docking models of the interaction between NAMPT and the iminosugar derivatives **21**–**30** (for their syntheses see Scheme 2 in Section 3.2) are shown in the Supplementary Materials (pages S43–S47).

### 3.2. Synthesis of the Iminosugar Derivatives

**Figure 9:**
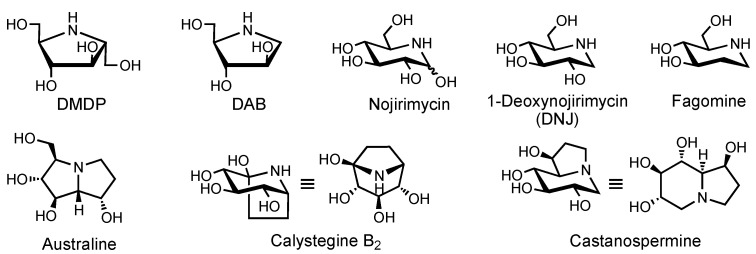
Structure of some naturally occurring, monocyclic and bicyclic iminosugars.

Iminosugars [15,16,17,18,19,20] are natural, enantiomerically pure compounds closely related to carbohydrates because they bear a basic nitrogen instead of the endocyclic oxygen atom (Figure 9). These monocyclic or bicyclic molecules are well-known inhibitors of both glycosidases (the enzymes that hydrolyses the glycosidic bond of oligosaccharides and glycoconjugates) [21,22] and glycosyltransferases (the enzymes that catalyze the formation of the glycosidic bond) [23,24,25,26,27,28].

In order to prepare new NAMPT inhibitors featuring the heterocycle-based pharmacophore that is carbon-linked to the anomeric position of the d-*ribo*-configured iminosugar, a few strategies already developed for the synthesis of simple *C*-iminofuranosides from natural carbohydrates could be exploited. One of these synthetic approaches, employed by Vogel and co-workers for the preparation of **1**, was first described by Horenstein and co-workers [29] and relies on the addition of Grignard reagents to the cyclic imine derived from an *O*-protected 1-deoxy-iminofuranose. Although promising, in our hands, this synthetic methodology led to a poor yield of the aryl *C*-iminofuranosides. Therefore, we decided to prepare the latter compounds taking advantage of a novel approach, recently published by Britton and co-workers [30], that is based on the organocatalysed, enantioselective tandem chlorination-aldol reaction of aliphatic aldehydes followed by conventional reductive amination. Moreover, we envisaged to synthesize the iminosugar derivatives **26**–**30** (Scheme 2), i.e., the tri-*O*-methylated analogues of the free-OH analogues **21**–**25**. In this manner, the esterification and/or oxidation reactions of the iminosugar alcohols that may occur in vivo should be greatly reduced or even suppressed.

**Scheme 3:**
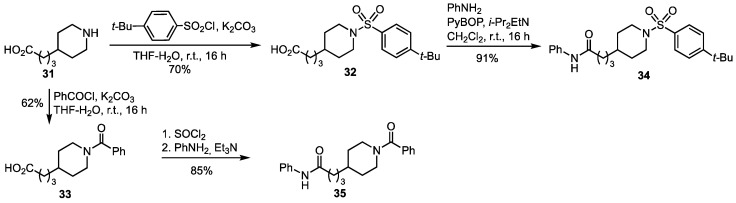
Synthesis of the potential NAMPT inhibitors **34** and **35**.

**Scheme 4:**
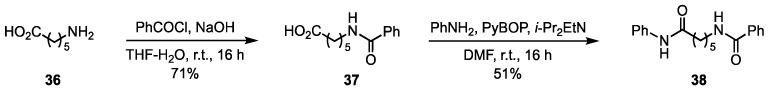
Synthesis of the potential NAMPT inhibitor **38**.

**Scheme 5:**
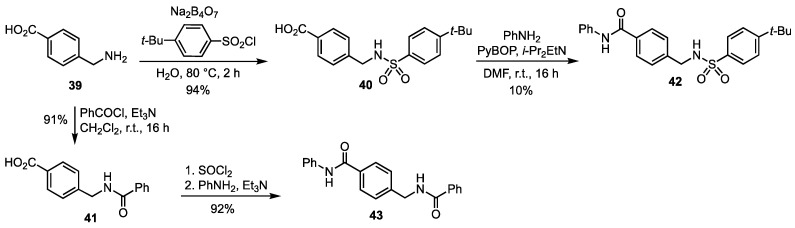
Synthesis of the potential NAMPT inhibitors **42** and **43**.

The commercially available 2-(4-nitrophenyl)-ethanol (**2**) was quantitatively oxidated to the aldehyde **3** (Scheme 2) that was treated with *N*-chlorosuccinimide (NCS) and 2,2-dimethyl-1,3-dioxan-5-one in the presence of l-proline, to first afford the corresponding α-chloroaldehyde as a racemic mixture [31]. Then, a proline-catalysed enantioselective aldol reaction took place between the (*R*)-configured α-chloroaldehyde and the dioxanone [32] to form a 3.6:1 mixture of the *syn*-adduct **4** and the *anti* diastereoisomer (not shown) from which the pure, known [30] compound **4** (99% *ee*) could be isolated in a 51% overall yield by column chromatography on silica gel. It is worth noting that the l-proline catalyzed the racemization of the unreacted (*S*)-α-chloroaldehyde, thus allowing a dynamic kinetic resolution [32]. The ketone **4** was then submitted to the reductive amination using benzyl amine and sodium cyanoborohydride, followed by the intramolecular nucleophilic substitution to afford the known [30] pyrrolidine **5** in a 44% isolated yield. This product was deprotected under acidic conditions to give the crude triol **6,** which was directly alkylated with benzyl bromide or methyl iodide, in the presence of sodium hydride, to afford **7** or **8**, respectively. After various reduction attempts, we found that the reaction of the *O*-and *N*-alkylated 4-nitrophenyl *C*-β-d-iminoribofuranosides **7** and **8** with indium and ammonium chloride in ethanol-water [33] cleanly gave the corresponding 4-aminophenyl derivatives **9** and **10**. These amines were not isolated but directly coupled with the carboxylic acids **32**, **33** (see Scheme 3 in Section 3.3), **37** (see Scheme 4 in Section 3.3), **40** and **41** (see Scheme 5 in Section 3.3) under standard conditions for the amide bond formation (PyBOP and *i*-Pr_2_EtN), to afford the tri-*O*-benzylated iminosugars **11**–**15** as well as the tri-*O*-methylated analogues **16**–**20** (Scheme 2). Finally, the catalytic hydrogenation of compounds **11**–**20** led to the corresponding *N*-deprotected iminofuranosides **21**–**30**. In the case of the methylated products **16**–**20**, the hydrogenation proceeded smoothly in methanol, whereas the tetra-benzylated products **11**–**15** required acidic conditions (AcOH as the solvent) to afford the fully deprotected compounds **21**–**25**. The detailed experimental procedures as well as the analytical and spectral data of compounds **3**–**30** are available in the Supplementary Materials (pages S3–S11). Moreover, copies of the NMR spectra of compounds **7**, **8**, **11**–**30** can be found in the Supplementary Materials (pages S14–S35).

### 3.3. Synthesis of the Non Iminosugar-Based Inhibitors

Aiming to evaluate the actual contribution of the iminosugar moiety in compounds **21**–**30** (see Scheme 2) to the NAMPT inhibition, we synthesized their analogues lacking the iminosugar unit. The commercially available 4-(piperidin-4-yl)-butanoic acid (**31**) was reacted with 4-*t*-butylbenzenesulfonyl chloride to give the acid **32** that was employed for the above-mentioned couplings (see Scheme 2), and for the synthesis of the potential inhibitor **34** by reaction with aniline (Scheme 3). On the other hand, the amino acid **31** was converted into the *N*-benzoyl derivative **33**, also used for the couplings shown in Scheme 2, which gave the target diamide **35** upon transformation into acyl chloride and reaction with aniline (Scheme 3).

The compound **38** was easily obtained by *N*-benzoylation of the commercial 6-aminohexanoic acid **36,** followed by coupling with aniline (Scheme 4). The known [34] acid intermediate **37** was also coupled with the *C*-iminoglycoside amines **9** and **10** to give the amides **13** and **18**, respectively (see Scheme 2).

The two potential NAMPT inhibitors **42** and **43** were prepared from the commercial 4-(aminomethyl) benzoic acid **39** (Scheme 5). The reaction of the latter with 4-*t*-butylbenzenesulfonyl chloride in the presence of sodium tetraborate, and amidation of the resulting, known [35] acid **40** with aniline afforded **42,** whereas **43** was obtained by *N*-benzoylation to give the known [36] acid **41** that was converted into the corresponding acid chloride and coupled with aniline. As already mentioned, the acid intermediates **40** and **41** were also exploited for the synthesis of the iminosugar amides **14**, **15**, **19** and **20** (see Scheme 2).

The detailed experimental procedures as well as the analytical and spectral data of compounds **32**–**35**, **37**, **38**, **40**–**43** are available in the Supplementary Materials (pages S11–S13). Copies of the NMR spectra of compounds **32**–**35**, **38**, **42**, **43** can be found in the Supplementary Materials (pages S36–S42).

### 3.4. Biological Tests

The NAMPT inhibition properties of the iminosugar-based products **21**–**25,** as well as their *O*-methylated analogues **26**–**30** (see Scheme 1) and the non-iminosugar derivatives **34**, **35**, **38**, **42**, **43,** were evaluated (Table 1) as described in the literature [37], using recombinant human NAMPT and compared to those of the benchmark inhibitor **FK866** (see Figure 2). It was found that ten new molecules (**22**, **23**, **24**, **26**, **28**, **29**, **34**, **38**, **42**, **43**) were able to inhibit the enzyme (IC_50_ = 43–603 μM), although less efficiently than the nanomolar inhibitor **FK866** (Table 1, entry 1), whereas the remaining five compounds were not inhibitors (IC_50_ > 1 mM). Upon analysis of the data outlined in Table 1, it appears that the NAMPT inhibition is not directly related to the presence of free or methylated hydroxyl groups onto the iminosugar unit. Indeed, the deprotected compound **21** is not an inhibitor (entry 2), while the methylated counterpart **26** (entry 3) inhibits the enzyme, and while for the analogues **22** and **27** the former is a rather good inhibitor (entry 5) but the *O*-methylated iminosugar derivative **27** does not inhibit the NAMPT (entry 6). Moreover, the *C*-iminoglycosides **23** and **28** (entries 8 and 9), as well as the couple **24** and **29** (entries 11 and 12), are all inhibitors, while the free-OH and *O*-methylated analogues **25** and **30**, respectively, are both lacking inhibition properties (entries 14 and 15). Finally, a clear relationship cannot be established between the presence of the iminosugar moiety linked to the pharmacophore and its enzyme inhibition activity, because in some cases the non-carbohydrate derivatives are stronger inhibitors than the iminosugar-based counterparts (**38** vs. **23** and **28**, **42** vs. **24** and **29**, **43** vs. **25** and **30**), while in the other cases the opposite is true (**34** vs. **21** and **26**, **35** vs. **22**).

Then, the new compounds were tested in vitro for their anti-proliferative effect, using the human pancreatic cancer cell line MiaPaca-2 in the cytotoxicity assays (Table 1). Nine out of fifteen compounds showed cells’ viability inhibition (IC_50_ = 32–920 μM), but to a much more limited extent when compared to that of **FK866** (IC_50_ = 2.4 nM). Again, the data collected in Table 1 cannot allow to determine the influence of the iminosugar moiety on the cell viability inhibition, since some non-carbohydrate derivatives were found to be more active than the iminosugar-based analogues (**35** vs. **22** and **27**, **38** vs. **23** and **28**, **43** vs. **25** and **30**), while in two cases the *C*-iminoglycosides, both tri-*O*-methylated, are better inhibitors (**26** and **29**).

**Figure 10:**
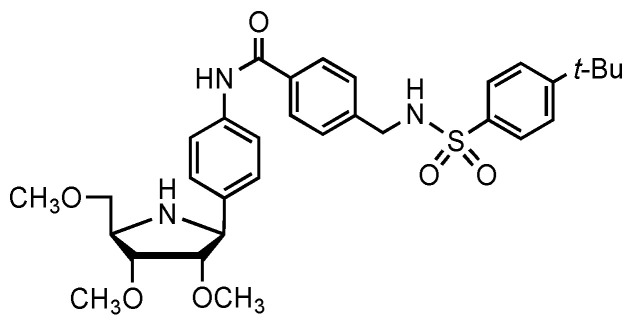
Structure of compound **29**, the most active iminosugar-based NAMPT-inhibitor synthetized in the present work (IC_50_ = 63 μM).

In order to confirm that the above-mentioned anti-proliferative effect of the new NAMPT inhibitors were associated with NAD^+^ depletion, we evaluated the intracellular NAD^+^ concentration (iNAD^+^) in MiaPaCa-2 cells for all the compounds (at a fixed 100 mM concentration). After 24 h, only the iminosugar-based inhibitors **22** (77%) and **29** (79%), as well as the non-carbohydrate analogue **42** (61%), led to significant depletion; the other compounds exerted an effect in the 0–10% range. Taken together, the biological data indicate that the tri-*O*-methylated compound **29** (Table 1, entry 12 and Figure 10) is the most promising member of this series of iminosugar-based NAMPT inhibitors, endowed with potential anticancer activity, and followed by another *O*-methylated derivative, **26** (entry 3), and the free-OH *C*-iminoglycoside **22** (entry 5).

## 4. Conclusions

Eleven years after the synthesis of a single iminosugar-based potential inhibitor of NAMPT reported by Vogel and co-workers [13], we have described in the present article a straightforward access to chemically stable inhibitors bearing a highly (free hydroxyl derivatives **21**–**25**) or moderately (*O*-methylated derivatives **26**–**30**) hydrophilic *C*-iminoribofuranoside moiety. One of them (compound **29**, Figure 10) was endowed with good enzyme inhibition activity and quite interesting in cellulo anticancer properties. Therefore, our work paves the way for further development of this poorly explored class of NAMPT inhibitors.

## Figures and Tables

**Table 1:** Inhibition of NAMPT enzymatic activity and MiaPaCa-2 cells viability, and intracellular NAD^+^ (iNAD^+^) depletion in 24 h on MiaPaCa-2 cells at 100 μM of inhibitor. The data reported for the inhibitor **FK866** (entry 1), obtained in our laboratory, are in agreement with those described in the literature (NAMPT inhibition [37]; anti-proliferative activity and iNAD depletion [38]). SD < 20% in all the measurements.

Entry	Compound	NAMPT Inhibition (IC_50_, μM)	MiaPaCa-2 Cells Viability (IC_50_, μM)	iNAD^+^ Depletion (%)
1	**FK866**	0.0033	0.0024	90
2	**21**	NI ^1^	NI ^2^	0
3	**26**	138.9	37	10
4	**34**	357	46.6	6
5	**22**	99.5	108	77
6	**27**	NI ^1^	539	0
7	**35**	NI ^1^	91.4	4
8	**23**	363	NI ^3^	5
9	**28**	603	NI ^3^	3
10	**38**	113	340	0
11	**24**	549	NI ^3^	9
12	**29**	63	32	79
13	**42**	43	NI ^3^	61
14	**25**	NI ^1^	NI ^3^	0
15	**30**	NI ^1^	920	0
16	**43**	283	698	1

^1^ No inhibition at concentration < 1 mM. ^2^ No inhibition at concentration < 100 μM. ^3^ No inhibition at concentration < 400 μM.

## Data Availability

The data supporting the reported results are available upon request from the corresponding author.

## Supplementary Materials

The following supporting information can be downloaded at: https://www.mdpi.com/article/10.3390/pharmaceutics15051472/s1, File S1: Experimental procedures for the synthesis of compounds **3**–**30**, **32**–**35**, **37**–**38**, **40**–**43**; ^1^H– and ^13^C–NMR spectra for compounds **7**, **8**, **11**, **12**–**14**, ^13^C–NMR spectrum for compound **15**; ^1^H– and ^13^C–NMR spectra for compounds **16**–**30**, **32**–**35**, **38**, **42**, **43**; docking figures for compounds **21**–**30**. Reference [39] is cited in Supplementary Materials.
