# Supplementary material for: Iminosugar-Based Nicotinamide Phosphoribosyltransferase (NAMPT) Inhibitors as Potential Anti-Pancreatic Cancer Agents
Source: Pharmaceutics. 2023 May 11;15(5):1472. doi: 10.3390/pharmaceutics15051472 (PMC10222582; doi:10.3390/pharmaceutics15051472)

Iminosugar-Based Nicotinamide Phosphoribosyltransferase (NAMPT) Inhibitors as Potential Anti-Pancreatic Cancer Agents

Irene Conforti,[1] Andrea Benzi,[2] Irene Caffa,[3,4] Santina Bruzzone,[2,4] Alessio Nencioni,[3,4] Alberto Marra[1,*]

1 Institut des Biomolécules Max Mousseron (IBMM, UMR 5247), Université de Montpellier, Pôle Chimie Balard Recherche, 1919 Route de Mende, CEDEX 5, 34293 Montpellier, France

2 Dipartimento di Medicina Sperimentale-DIMES, Scuola di Scienze Mediche e Farmaceutiche, Università degli Studi di Genova, Viale Benedetto XV 1, 16132 Genova, Italy

3 Dipartimento di Medicina Interna e Specialità Mediche-DIMI, Università degli Studi di Genova, Viale Benedetto XV 6, 16132 Genova, Italy

4 IRCCS, Ospedale Policlinico San Martino, 16132 Genova, Italy

* Correspondence: alberto.marra@umontpellier.fr

Supporting Information

Table of contents page

Synthesis, analytical and spectral data of **3-7** S3
Synthesis, analytical and spectral data of **8-11** S4
Synthesis, analytical and spectral data of **12** and **13** S5
Synthesis, analytical and spectral data of **14-16** S6
Synthesis, analytical and spectral data of **17-19** S7
Synthesis, analytical and spectral data of **20-22** S8
Synthesis, analytical and spectral data of **23-26** S9
Synthesis, analytical and spectral data of **27-29** S10
Synthesis, analytical and spectral data of **30**, **32**, **33** S11
Synthesis, analytical and spectral data of **34**, **35**, **37**, **38** S12
Synthesis, analytical and spectral data of **40-43** S13
^{1}H- and ^{13}C-NMR spectra of **7** S14
^{1}H- and ^{13}C-NMR spectra of **8** S15
^{1}H- and ^{13}C-NMR spectra of **11** S16
^{1}H- and ^{13}C-NMR spectra of **12** S17
^{1}H- and ^{13}C-NMR spectra of **13** S18
^{1}H- and ^{13}C-NMR spectra of **14** S19
^{1}H-NMR spectrum of **15** S20
^{1}H- and ^{13}C-NMR spectra of **16** S21

^{1}H- and ^{13}C-NMR spectra of **17** S22

^{1}H- and ^{13}C-NMR spectra of **18** S23

^{1}H- and ^{13}C-NMR spectra of **19** S24

^{1}H- and ^{13}C-NMR spectra of **20** S25

^{1}H- and ^{13}C-NMR spectra of **21** S26

^{1}H- and ^{13}C-NMR spectra of **22** S27

^{1}H- and ^{13}C-NMR spectra of **23** S28

^{1}H- and ^{13}C-NMR spectra of **24** S29

^{1}H- and ^{13}C-NMR spectra of **25** S30

^{1}H- and ^{13}C-NMR spectra of **26** S31

^{1}H- and ^{13}C-NMR spectra of **27** S32

^{1}H- and ^{13}C-NMR spectra of **28** S33

^{1}H- and ^{13}C-NMR spectra of **29** S34

^{1}H- and ^{13}C-NMR spectra of **30** S35

^{1}H- and ^{13}C-NMR spectra of **32** S36

^{1}H- and ^{13}C-NMR spectra of **33** S37

^{1}H- and ^{13}C-NMR spectra of **34** S38

^{1}H- and ^{13}C-NMR spectra of **35** S39

^{1}H- and ^{13}C-NMR spectra of **38** S40

^{1}H- and ^{13}C-NMR spectra of **42** S41

^{1}H- and ^{13}C-NMR spectra of **43** S42

Docking of **21** and **22** S43

Docking of **23** and **24** S44

Docking of **25** and **26** S45

Docking of **27** and **28** S46

Docking of **29** and **30** S47

***p*-Nitrophenylacetaldehyde (3)**. A mixture of alcohol **2** (2.10 g, 12.5 mmol) and Dess-Martin periodinane (6.39 g, 15 mmol) in dry CH_2Cl_2 (40 mL) was stirred at room temperature for 16 h and then quenched by addition of 1M aq. $Na_2S_2O_3$ (30 mL) and sat. aq. $NaHCO_3$ (175 mL). The aqueous phase was extracted with CH_2Cl_2 (200 mL), and the organic phase was washed with sat. aq. $NaHCO_3$ (3 x 200 mL), dried over Na_2SO_4, and concentrated to give the known [39] aldehyde **3** in quantitative yield as an orange solid. ^{1}H NMR (400 MHz, $CDCl_3$): δ 9.82 (t, 1H, *J* = 1.7 Hz, CHO) 8.24 (d, 2H, *J* = 8.8 Hz, Ar), 7.40 (d, 2H, *J* = 8.8 Hz, Ar), 3.87 (d, 2H, *J* = 1.7 Hz, CH_2).

4-[(1*R*,2*R*)-2-chloro-1-hydroxy-2-(4-nitrophenyl)ethyl]-2,2-dimethyl-1,3-dioxan-5-one (4). To a cooled (0 °C), stirred solution of aldehyde **3** (2.10 g, 12.7 mmol), NCS (1.78 g, 12.7 mmol), and l-proline (1.17 g, 7.6 mmol) in dry CH_2Cl_2 (64 mL) was added 2,2-dimethyl-1,3-dioxan-5-one (1.59 mL, 12.7 mmol) and the mixture was stirred at 0 °C for 72 h. The reaction mixture was then diluted with CH_2Cl_2 (350 mL) and the organic phase was washed with water (2 x 250 mL) and brine (250 mL), dried (Na_2SO_4), and concentrated. The product was purified by flash chromatography (cyclohexane:EtOAc, 9:1 to 8:2) to afford the known [30] compound **4** (2.01 g, 51% yield) as a yellow solid. ^{1}H NMR (400 MHz, $CDCl_3$): δ 8.22 (d, 2H, *J* = 8.8 Hz, Ar), 7.72 (d, 2H, *J* = 8.8 Hz, Ar), 5.31 (d, 1H, *J* = 2.3 Hz, CHCl), 4.47 (dd, 1H, *J* = 1.5, 8.2 Hz, CHCO), 4.27 (dd, 1H, *J* = 1.5, 17.7 Hz, CH_2), 4.14 (ddd, 1H, *J* = 2.3, 8.2 Hz, C*H*OH), 4.09 (d, 1H, *J* = 17.7 Hz, CH_2), 1.56 (s, 3H, CH_3), 1.48 (s, 3H, CH_3).

1-C-(1*S*)-1-[4-(*N*-benzyl-3,5-*O*-isopropylidene)-nitrophenyl]-1,4-dideoxy-1,4-imino-D-ribitol (5). To a solution of **4** (2.01 g, 6.1 mmol) in dry THF (60 mL) were added acetic acid (0.26 mL, 6.1 mmol) and $BnNH_2$ (1.66 mL, 15.2 mmol), the solution was stirred at room temperature for 2 h, then $NaCNBH_3$ (0.955 g, 15.2 mmol) was added, and the mixture was vigorously stirred for two additional hours. Then the reaction mixture was diluted with CH_2Cl_2 (300 mL) and washed with water (250 mL) and brine (250 mL), dried (Na_2SO_4) and concentrated. Purification of the crude product by flash chromatography (cyclohexane:EtOAc, 3:1) afforded the known [30] iminofuranoside **5** (1.40 g, 44%) as a yellow solid. ^{1}H NMR (400 MHz, $CDCl_3$): δ 8.21 (d, 2H, *J* = 8.7 Hz, Ar), 7.67 (d, 2H, *J* = 8.7 Hz, Ar), 7.26-7.21 (m, 5H, NCH_2*Ph*), 4.00 (d, 1H, $J_{2,3}$ = 4.6 Hz, H-2), 3.84 (s, 1H, H-1), 3.81 and 3.56 (2 d, 2H, *J* = 12.9 Hz, N*CH₂*Ph), 3.70 (dd, 1H, $J_{3,4}$ = 9.8 Hz, H-3), 3.53 (dd, 1H, $J_{5,5'}$ = 10.4 Hz, H-5), 3.36 (dd, 1H, $J_{4,5'}$ = 4.1 Hz, H-5'), 2.95 (ddd, 1H, H-4), 2.38 (br s, 1H, OH), 1.43 (s, 3H, CH_3), 1.41 (s, 3H, CH_3).

1-C-(1*S*)-1-(*N*-benzyl-*p*-nitrophenyl)-1,4-dideoxy-1,4-imino-D-ribitol (6). A solution of the known [30] *C*-iminofuranoside **5** (0.95 g, 2.5 mmol) in acetic acid 80% (25 mL) was stirred at 100 °C for 1 h, then concentrated. The crude product **6** was directly used in the following step.

1-C-(1*S*)-1-[4-(*N*-benzyl-2,3,5-tri-*O*-benzyl)-nitrophenyl]-1,4-dideoxy-1,4-imino-D-ribitol (7). The crude **6** (0. 86 g, 2.5 mmol) was dissolved in dry DMF (10 mL) under argon atmosphere. Sodium hydride (60% dispersion in mineral oil) was added in 2 portions (0.72 g, 30 mmol) at 0 °C and the mixture was stirred at this temperature for 30 min. Then benzyl bromide (1.78 mL, 15 mmol) was added and stirring was continued at room temperature for 15 h. Methanol (6 mL) was added dropwise, and the mixture stirred for an additional hour. The reaction mixture was then concentrated, and the residue was dissolved with a mixture of CH_2Cl_2 (80 mL) and water (35 mL). The organic phase was then washed again with brine (35 mL), dried (Na_2SO_4), filtered and concentrated. The yellowish residue was purified by column chromatography (cyclohexane:AcOEt, 10:0 to 8:2) to give **7** as a yellow oil (0.63 g, 41%); $[\alpha]_D$ = +17.3 (c 1.0 in $CHCl_3$). ^{1}H NMR (400 MHz, $CDCl_3$): δ 8.17–8.10 (d, 2H, *J* = 8.8 Hz, Ar), 7.68–7.60 (d, 2H, *J* = 8.8 Hz, Ar), 7.40–7.28 (m, 8H, Ar), 7.25–7.13 (m, 10H, Ar), 7.00–6.94 (m, 2H, Ar), 4.69 and 4.60 (2 d, 2H, *J* = 12.3 Hz, PhCH_2), 4.39 and 4.32 (2 d, 2H, *J* = 12.0 Hz, PhCH_2), 4.37 and 4.14 (2 d, 2H, *J* = 12.1 Hz, PhCH_2) 4.25 (d, 1H, *J* = 8.9 Hz, H-2), 3.80

and 3.61 (2 d, 2H, *J* = 13.1 Hz, PhC*H*$_2$N), 3.93 (d, 1H, *J* = 4.6 Hz, H-1), 3.61 (m, 1H, H-3), 3.32 (dd, 1H, *J* = 7.6, 3.8 Hz, H-4), 3.11–3.07 (m, 2H, H-5,H-5'). ^{13}C NMR (100 MHz, CDCl$_3$): δ 150.3, 147.4, 138.3, 138.2, 138.1, 137.6 (C Ar), 129.5, 128.7, 128.5, 128.5, 128.4, 128.3, 128.3, 127.8, 127.7, 127.7, 127.7, 127.6, 127.4, 123.5 (CH Ar), 84.3 (C-3), 77.6 (C-1), 73.38 (Ph*C*H$_2$), 71.9 (C-5), 71.7, 70.8 (Ph*C*H$_2$), 69.8 (C-2), 66.9 (C-4), 58.5 (NCH$_2$Ph). HRMS (ESI/Q-TOF): *m/z* calcd. for $C_{39}H_{39}N_2O_5$ [M+H]$^+$ 615.2853, found 615.2853.

1-C-(1*S*)-1-[4-(*N*-benzyl-2,3,5-tri-*O*-methyl)-nitrophenyl]-1,4-dideoxy-1,4-imino-D-ribitol (8). The crude **6** (2.22 g, 0.37 mmol) was dissolved in dry DMF (5 mL) under argon atmosphere. Sodium hydride (60% dispersion in mineral oil) was added in two portions (0,143 g, 4.4 mmol) at 0 °C and the mixture was stirred at this temperature for 30 min. Then iodomethane (0.27 mmol, 4.4 mmol) was added and stirring was continued at room temperature for 2.5 h. Methanol (3 mL) was added dropwise, and the mixture was stirred for 1 additional hour. The reaction mixture was concentrated, and the residue was then dissolved with a mixture of CH_2Cl_2 (50 mL x 2) and water (35 mL). The organic phase was then washed again with brine (35 mL), dried (Na_2SO_4), and concentrated. The yellowish residue was purified by column chromatography (cyclohexane:AcOEt, 10:0 to 7:3) to give the compound **8** as a yellow oil (58 mg, 41%); $[\alpha]_D$ = -30.0 (c 1.0 in CHCl$_3$). ^{1}H NMR (400 MHz, CDCl$_3$): δ 8.19 (d, 2H, *J* = 8.8 MHz, Ar), 7.69 (d, 2H, *J* = 8.8 MHz, Ar), 7.29–7.18 (m, 5H, *Ph*CH$_2$), 4.09 (d, 1H, *J* = 8.7 Hz, H-2), 3.76 and 3.58 (2 d, 2H, *J* = 13 Hz, NC*H*$_2$Ph), 3.76 (m, 1H, H-1), 3.50 (dd, 1H, *J* = 8.7, 4.4 Hz, H-3), 3.43 (s, 3H,), 3.25 (s, 3H, CH$_3$), 3.24 (s, 3H, CH$_3$), 3.22–3.18 (m, 1H, H-5), 3.09–3.01 (m, 2H, H-5', H-4). ^{13}C NMR (100 MHz, CDCl$_3$): δ 150.3, 147.4, 138.1 (C), 129.4, 128.6, 128.2, 123.6 (CH Ar), 86.6 (C-3), 80.5 (C-2), 74.2 (C-4), 69.6 (C-1), 65.6 (C-5), 59.0, 58.4 (CH$_3$), 58.0 (N*C*H$_2$Ph), 57.0 (CH$_3$). HRMS (ESI/Q-TOF): *m/z* calcd. for $C_{21}H_{27}N_2O_5$ [M+H]$^+$ 387.1914, found 387.1914.

1**-C-(1*S*)-1-[4-(*N*-benzyl-2,3,5-tri-*O*-benzyl)-aminophenyl]-1,4-dideoxy-1,4-imino-D-ribitol (9)**. To a solution of **7** (1.53 g, 2.5 mmol) in absolute ethanol (10 mL) was added aqueous saturated ammonium chloride solution (3 mL) and indium powder (2.0 g, 17.4 mmol). The mixture was stirred under reflux for 12 h and monitored by LC-MS to confirm the complete consumption of the starting material. The cooled reaction mixture was diluted with water (50 mL) and filtered through Celite. The aqueous filtrate was adjusted to pH 9 with NaOH 1 M and extracted with CH_2Cl_2 (3 x 15 mL). The combined organic layers were dried (Na_2SO_4) and concentrated. The crude product was directly used in the following step. ^{1}H NMR (400 MHz, CDCl$_3$ with K_2CO_3): δ 7.48–7.43 (m, 2H, Ar), 7.42–7.38 (m, 2H, Ar), 7.26–6.99 (m, 18H, Ar), 6.45–6.39 (m, 2H, Ar), 4.71–4.63 (m, 2H, PhC*H*$_2$), 4.37–4.29 (m, 2H, PhC*H*$_2$), 4.26–4.18 (m, 3H, PhC*H*$_2$, H-2), 4.08 (d, 1H, *J* = 4.5, H-1), 3.96 and 3.54 (2 d, 2H, *J* = 13.4 Hz, PhC*H*$_2$N), 3.82 (m, 1H, H-3), 3.47–3.39 (m, 1H, H-4), 3.17–3.08 (m, 2H, H-5, H-5').

1-C-(1*S*)-1-[4-(*N*-benzyl-2,3,5-tri-*O*-methyl)-aminophenyl]-1,4-dideoxy-1,4-imino-D-ribitol (10). Compound **8** (0.89 g, 2.5 mmol) was treated as described for the synthesis of **9** to give **10** that was directly used in the following step.

1-C-(1*S*)-1-[(1-(*N*-benzyl-2,3,5-tri-*O*-benzyl)-(4-*tert*-butylphenyl)sulfonyl)piperidin-4-yl)-butanamidophenyl]-1,4-dideoxy-1,4-imino-D-ribitol (11). A solution of **9** (70 mg, 0.12 mmol), **32** (66 mg, 0.18 mmol) and PyBOP (106 mg, 0.21 mmol) in dry DMF (1.5 mL) was cooled to 0 °C then *i*-Pr_2NEt (0.04 mL, 0.24 mmol) was slowly added, and the reaction mixture was stirred at room temperature for 16 h. The solvent was removed, and a solution of the residue in AcOEt (20 mL) washed with sat. aq. solution of NH_4Cl (2 x 20 mL), dried (Na_2SO_4), filtered and concentrated. The residue was purified by flash column chromatography (cyclohexane:AcOEt, from 10:0 to 7:3) to give **11** (44.5 mg, 27%) as a colourless oil; $[\alpha]_D$ = -24.2 (c 0.75 in CHCl$_3$). ^{1}H NMR (CDCl$_3$): δ 7.69–7.65

(d, 2H, J = 8.5 Hz, Ar), 7.54–7.50 (d, 2H, J = 8.5 Hz, Ar), 7.48–7.45 (m, 2H, Ar), 7.36–7.27 (m, 10H, Ar), 7.24–7.10 (m, 10H, Ar), 7.01 (d, 2H, J = 8.6 Hz, Ar), 4.64 and 4.57 (2 d, 2H, J = 12.3 Hz, PhCH_2), 4.31 and 4.19 (2 d, 2H, J = 12.1 Hz, PhCH_2), 4.30–4.24 (m, 2H, PhCH_2), 4.11–4.07 (m, 1H, H-2), 3.89–3.85 (m, 1H, H-1), 3.81 and 3.49 (2d, 2H, J = 13.1 Hz, NCH_2Ph), 3.79–3.75 (m, 2H, CH_2NCH_2), 3.61–3.59 (m, 1H, H-3), 3.26–3.20 (m, 1H, H-4), 3.05–2.95 (m, 2H, H-5, H-5'), 2.32 (t, 2H, J = 7.4 Hz, NCOCH_2), 2.24 (t, 2H, J = 11.4, CH_2NCH_2), 1.75 (m, 2H, CH_2CH$_2$NCH$_2$CH_2), 1.74 (quint, 2H, CH_2CH$_2$CON), 1.35 (s, 9H, t-Bu), 1.33–1.19 (m, 5H, CH_2CH$_2$NCH$_2$CH_2, CH, CH_2CH$_2$CH$_2$CON). ^{13}C NMR (CDCl$_3$): δ 171.3 (CO), 156.4, 138.9, 138.6, 138.2, 138.0, 137.2, 133.4, 129.2, 128.8 (C Ar), 129.6, 128.5, 128.4, 128.4, 128.2, 127.7, 127.7, 127.6, 127.5, 127.5, 127.4, 127.4, 127.2, 127.1, 126.04, 119.7 (CH Ar), 84.3 (C-3), 78.2 (C-1), 73.2 (PhCH$_2$), 72.0 (PhCH$_2$, PhCH$_2$), 70.8 (C-5, C-5'), 69.7 (C-2), 66.6 (C-4), 57,7 (PhCH$_2$), 46.6 (CH$_2$NCH$_2$), 37.9 (CH$_2$CON), 35.8 (CH$_2$CH$_2$CH$_2$CON), 35.3 (CH), 31.6 (CH$_2$CH$_2$NCH$_2$$CH_2$), 31.2 (C t-Bu), 31.45 (t-Bu), 22.8 (CH$_2$CH$_2$CON). HRMS (ESI/Q-TOF): m/z calcd. for $C_{58}H_{68}N_3O_6S$ [M+H]$^+$ 934.4829, found 934.4833.

1-C-(1*S*)-1-[4-(1-(*N*-benzyl-2,3,5-tri-*O*-benzyl)-benzoylpiperidin-4-yl)-butanamido-phenyl]-1,4-dideoxy-1,4-imino-D-ribitol (12). The iminosugar **9** (20 mg, 0.12 mmol) was allowed to react with the acid **33** (27 mg, 0.24 mmol) as described for the synthesis of **11** to give, after the same work-up and purification (CH$_2$Cl$_2$:AcOEt, 100:0 to 75:25), **12** (63.8 mg, 63%) as a colourless oil; [α]$_D$ = -23.6 (c 1.0 in CHCl$_3$). ^{1}H NMR (DMSO-d6): δ 9.85 (s, 1H, NH), 7.56 (d, 2H, J = 8.3 Hz, Ar), 7.44–7.39 (m, 3H, Ar), 7.40–7.15 (m, 22H, Ar), 7.08–7.03 (m, 2H, Ar), 4.54 and 4.46 (2 d, 2H, J = 12.0 Hz, PhCH_2), 4.52–4.49 (m, 2H, CH_2NCH_2), 4.38–4.31 (m, 3H, PhCH_2, PhCH_2), 4.21 (d, 1H, J = 12.1 Hz, PhCH_2), 3.96–3.88 (m, 2H, H-2, H-1), 3.75 and 3.48 (2 d, 2H, J = 13.6 Hz, PhCH_2N), 3.69 (dd, 1H, J = 8.4, 4.5 Hz, H-3), 3.24–3.09 (m, 3H, H-4, H-5, H-5'), 3.14–3.01 (m, 2H, CH_2NCH_2), 2.29 (t, 2H, J = 7.3 Hz, CH$_2$CON), 1.62 (quint, 2H, J = 7.5 Hz, CH_2CH$_2$CON), 1.57–1.47 (m, 1H, CH) 1.35–1.16 (m, 4H, CH_2CH$_2$NCH$_2$CH_2), 1.16 - 1.00 (m, 2H, CH_2CH$_2$CH$_2$CON). ^{13}C NMR (DMSO-d6): δ 171.0 (CO), 168.8 (CO), 138.5, 138.5, 138.4, 138.3, 138.1, 136.5, 136.1 (C Ar), 129.2, 128.4, 128.4, 128.2, 128.2, 128.1, 128.1, 128.0, 127.8, 127.4, 127.4, 127.3, 127.2, 127.1, 127.0, 126.6, 119.0 (CH Ar), 84.0 (C-3), 78.0 (C-1), 72.2 (PhCH$_2$), 71.3 (PhCH$_2$), 70.7 (PhCH$_2$), 70.1 (PhCH$_2$), 68.6 (C-2), 65.6 (C-4), 56.1 (NCH$_2$Ph), 47.7 (CH$_2$NCH$_2$), 36.5 (CH$_2$CON), 35.4 (CH), 35.2 (CH$_2$CH$_2$CH$_2$CON), 31.7 (CH$_2$CH$_2$NCH$_2$$CH_2$), 22.8 ($CH_2CH_2$CON). HRMS (ESI/Q-TOF): m/z calcd. for $C_{55}H_{60}N_3O_5$ [M+H]$^+$ 842.4539, found 842.4527.

1-C-(1*S*)-1-[(6-benzamide)-1-hexylamidophenyl-(*N*-benzyl-2,3,5-tri-*O*-benzyl)]-1,4-dideoxy-1,4-imino-D-ribitol (13). The iminosugar **9** (94 mg, 0.16 mmol) was allowed to react with the acid **37** (75.6 mg, 0.32 mmol) as described for the synthesis of **11** to give, after the same work-up and purification by semi-preparative reverse-phase HPLC (linear gradient H$_2$O + 0.01% TFA/acetonitrile + 0.01% TFA, 60:40 to 0:100 in 30 min), **13** (34 mg, 26%) as a colourless oil; [α]$_D$ = -26.1 (c 1.0 in CHCl$_3$). ^{1}H NMR (CDCl$_3$): δ 8.55 (s, 1H, NH), 7.76 (d, 2H, J = 7.2 Hz, Ar), 7.58 (d, 2H, J = 8.2 Hz, Ar), 7.52–7.46 (m, 1H, Ar), 7.45–7.11 (m, 22H, Ar), 7.01–6.96 (m, 2H, Ar), 6.92–6.87 (m, 1H, NH), 4.58 and 4.38 (d, 2H, J = 11.8 Hz, PhCH_2), 4.56 (d, 1H, J = 13.1 Hz, PhCH_2), 4.51 and 4.43 (d, 2H, J = 11.3 Hz, PhCH_2), 4.41–4.34 (m, 2H, H-2, H-1), 4.29–4.20 (m, 3H, PhCH_2), 3.99–3.92 (m, 1H, H-3), 3.86–3.81(m, 1H, H-4), 3.81–3.75 (m, 1H, H-5), 3.71–3.66 (m, 1H, H-5'), 3.51–3.44 (m, 2H, CH$_2$N), 2.45 (t, 2H, J = 7.3 Hz, CH$_2$CO), 1.78 (quint, 2H, J = 7.3 Hz, CH_2CH$_2$CO), 1.68 (quint, 2H, J = 7.2 Hz, CH_2CH$_2$N), 1.47 (quint, 2H, J = 7.6 Hz, CH_2CH$_2$CH$_2$CO). ^{13}C NMR (CDCl$_3$): δ 173.5 (CO), 169.5 (CO), 139.8, 136.7, 136.5, 136.4, 133.7, 132.3, 132.3 (C Ar), 132.1, 130.7, 129.7, 129.5, 128.8, 128.8, 128.8, 128.7, 128.5, 128.5, 128.4, 128.3, 128.2, 127.3, 127.17, 125.74, 120.90 (CH Ar), 80.4 (C-2), 76.4 (C-3), 73.9 (PhCH$_2$), 73.5 (PhCH$_2$), 73.0 (PhCH$_2$), 70.9 (C-1), 68.7 (C-4), 66.7 (C-

5), 57.4 (NCH$_2$Ph), 40.1 (CH$_2$N), 37.1 (CH$_2$CO), 28.7 (*C*H$_2$CH$_2$N), 26.02 (*C*H$_2$CH$_2$CH$_2$CO), 24.85 (*C*H$_2$CH$_2$CO). HRMS (ESI/Q-TOF): *m/z* calcd. for $C_{52}H_{56}N_3O_5$ [M+H]$^+$ 802.4214, found 802.4208.

1-C-(1*S*)-1-[(1-(*N*-benzyl-2,3,5-tri-*O*-benzyl)-(4-*tert*-butylphenyl)sulfonamido)methyl)-benzamide]-1,4-dideoxy-1,4-imino-D-ribitol (14). The iminosugar **9** (108 mg, 0.18 mmol) was allowed to react with the acid **40** (128 mg, 0.36 mmol) as described for the synthesis of **11** to give, after the same work-up and purification (cyclohexane:AcOEt, 75:25 to 60:40), **14** (40 mg, 39%) as a colourless oil; [α]$_D$ = -8.0 (c 0.4 in CHCl$_3$). ^{1}H NMR (CDCl$_3$): δ 7.81–7.72 (m, 7H, Ar), 7.58–7.53 (m, 2H, Ar), 7.50–7.45 (m, 4H, Ar), 7.33–7.21 (m, 12H, Ar), 7.19–7.10 (m, 9H, Ar), 7.04–7.00 (m, 2H, Ar), 4.91 (t, 1H, *J* = 6.4 Hz, NH) 4.66 and 4.57 (2 d, 2H, *J* = 12.4 Hz, PhC*H$_2$*), 4.35–4.25 (m, 4H, PhC*H$_2$*, CH$_2$N), 4.24–4.20 (m, 2H, PhC*H$_2$*), 4.16–4.12 (m, 1H, H-2), 3.89 (d, 1H, *J* = 4.6 Hz, H-1), 3.84 and 3.53 (2 d, 2H, *J* = 13.3 Hz, NPhC*H$_2$*), 3.62 (dd, 1H, *J* = 8.9, 4.4 Hz, H-3), 3.28–3.23 (m, 1H, H-4), 3.0–2.94 (m, 2H, CH$_2$), 1.35 (s, 9H, *t*-Bu). ^{13}C NMR (CDCl$_3$): δ 165.2 (CO), 156.9, 140.6, 138.9, 138.6, 138.5, 138.5, 138.2, 137.1, 136.8, 134.7 (C Ar), 129.6, 128.8, 128.6, 128.4, 128.4, 128.4, 128.3, 128.3, 128.2, 127.7, 127.6, 127.6, 127.5, 127.2, 127.1, 126.4, 120.9, 120.2 (CH Ar), 84.3 (C-3), 78.2 (C-1), 73.2 (PhCH$_2$), 72.0 (C-5, PhCH$_2$), 70.8 (PhCH$_2$), 69.7 (C-2), 66.6 (C-4), 57.7 (NCH$_2$Ph), 47.0 (CH$_2$), 31.24 (t-Bu). HRMS (ESI/Q-TOF): *m/z* calcd. for $C_{57}H_{60}N_3O_6S$ [M+H]$^+$ 914.4197, found 914.4187.

1-C-(1*S*)-1-[4-(benzamidomethyl)-benzamide-(*N*-benzyl-2,3,5-tri-*O*-benzyl)-phenyl]-1,4-dideoxy-1,4-imino-D-ribitol (15). The iminosugar **9** (60 mg, 0.1 mmol) was allowed to react with the acid **41** (53 mg, 0.2 mmol) as described for the synthesis of **11** to give, after the same work-up and purification (cyclohexane:AcOEt:MeOH 7:3:0 to 6:4:1), **15** (60 mg, 54%) as a colourless oil slightly contaminated by uncharacterized by-products. This compound was employed in the next step without further purification. ^{1}H NMR (CD$_3$Cl): δ 8.07 (s, 1H, NH), 7.81–7.70 (m, 4H, CH Ar), 7.60–7.58 (m, 2H, CH Ar), 7.48–7.38 (m, 3H, CH Ar), 7.38–7.18 (m, 11H, CH Ar), 7.18–7.06 (m, 10H, CH Ar), 6.98–6.88 (m, 2H, CH Ar), 6.74 (t, 1H, *J* = 6.0 Hz, NH), 4.57 (m, 3H, C*H$_2$*Ph, C*H$_2$*Ph), 4.50 (d, 1H, *J* = 12.3 Hz, C*H$_2$*Ph), 4.29–4.01 (m, 4H, 2 C*H$_2$*Ph), 4.06 (d, 1H, *J* = 8.8 Hz, H1), 3.82 (d, 1H, *J* = 4.6 Hz, H2), 3.77 (d, 1H, *J* = 13.3 Hz, CH$_2$NH), 3.55 (dd, 1H, *J* = 8.7, 4.6 Hz, H3), 3.44 (d, 1H, *J* = 13.2 Hz, CH$_2$NH), 3.20 - 3.17 (m, 1H, H4), 2.96 - 2.93 (m, 2H, H5, H5').

1-C-(1*S*)-1-[(1-(4-*tert*-butylphenyl)sulfonyl)piperidin-4-yl)-(*N*-benzyl-2,3,5-tri-*O*-methyl)butanamidophenyl]-1,4-dideoxy-1,4-imino-D-ribitol (16). The iminosugar **10** (35 mg, 0.1 mmol) was allowed to react with the acid **32** (72 mg, 0.2 mmol) as described for the synthesis of **11** to give, after the same work-up and purification by semi-preparative reverse-phase HPLC (linear gradient H$_2$O + 0.01% TFA/acetonitrile + 0.01% TFA, 60:40 to 0:100 in 20 min), **16** (130 mg, 38%) as an amorphous white solid; [α]$_D$ = +23 (c 0.75 in CHCl$_3$). ^{1}H NMR (CDCl$_3$): δ 7.68–7.64 (d, 2H, *J* = 8.6 Hz, Ar), 7.53–7.50 (d, 2H, *J* = 8.6 Hz, Ar), 7.50–7.43 (m, 3H, Ar), 7.34–7.30 (m, 1H, Ar), 7.26–7.16 (m, 5H, Ar), 3.90 (d, 1H, *J* = 8.8 Hz, H-2), 3.80–3.74 (m, 4H, PhCH$_2$, CH$_2$NCH$_2$), 3.72–3.69 (m, 1H, H-1), 3.48–3.41 (m, 3H, H-3, PhCH$_2$), 3.39 (s, 3H, CH$_3$), 3.11-3.06 (m, 1H, H-4), 3.19 (s, 3H, CH$_3$), 3.17 (s, 3H, CH$_3$), 3.02-2.98 (m, 2H, H-5, H-5'), 2.34 (t, 2H, *J* = 7.5 Hz, CH$_2$CON), 2.35–2.27 (m, 2H, CH$_2$NCH$_2$), 1.76–1.72 (m, 2H, CH$_2$CH$_2$NCH$_2$CH$_2$), 1.72–1.63 (m, 2H, CH$_2$CH$_2$CON), 1.36 (s, 9H, *t*-Bu), 1.32–1.17 (m, 5H, CH$_2$CH$_2$NCH$_2$CH$_2$, CH$_2$CH$_2$CH$_2$CON, CH). ^{13}C NMR (CDCl$_3$): δ 171.1 (CO), 156.4, 138.9, 137.9, 137.3, 133.3 (C Ar), 129.5, 128.6, 128.2, 127.7, 127.2, 126.0, 119.9 (CH Ar), 86.5 (C-3), 80.8 (C-1), 74.4 (C-5), 69.6 (C-2), 65.3 (C-4), 59.0, 58.1 (CH$_3$), 57.7 (NCH$_2$Ph), 57.0 (CH$_3$), 46.5 (CH$_2$NCH$_2$), 37.8 (*C*H$_2$CON), 36.8 (CH$_2$CH$_2$CH$_2$CON), 36.3 (CH), 31.3 (CH$_2$CH$_2$NCH$_2$CH$_2$), 31.2 (C t-Bu), 25.8(CH$_2$CH$_2$CON). HRMS (ESI/Q-TOF): *m/z* calcd. for $C_{40}H_{56}N_3O_6S$ [M+H]$^+$ 702.3814, found 702.3810.

1-C-(1*S*)-1-[(4-(1-benzoylpiperidin-4-yl)-butanamidophenyl)-(*N*-benzyl-2,3,5-tri-*O*-methyl)]-1,4-dideoxy-1,4-imino-D-ribitol (17). The iminosugar **10** (40 mg, 0.11 mmol) was allowed to react with the acid **33** (62 mg, 0.22 mmol) as described for the synthesis of **11** to give, after the same work-up and purification by semi-preparative reverse-phase HPLC (linear gradient H_2O + 0.01% TFA/acetonitrile + 0.01% TFA, 50:50 to 0:100 in 20 min), **17** (11 mg, 16%) as a colourless oil; $[\alpha]_D$ = +20.9 (c 0.8 in $CHCl_3$). ^{1}H NMR (CD_3OD): δ 8.19 (s, 1H, NH), 7.66 (d, 2H, *J* = 8.3 Hz, Ar), 7.51–7.45 (m, 2H, Ar), 7.45–7.33 (m, 10H, Ar), 4.70 (d, 1H, *J* = 12.9 Hz, CH_2NCH_2), 4.61 (d, 1H, *J* = 13.3 Hz, NCH_2Ph), 4.29–4.19 (m, 3H, NCH_2Ph, H-2, H-1), 4.00–3.88 (m, 2H, H-5, H-3), 3.85–3.76 (m, 3H, H-5', H-4, CH_2NCH_2), 3.43, 3.40 and 3.19 (3 s, 9H, 3 CH_3), 3.02 (t, 1H, *J* = 12.9 Hz, CH_2NCH_2), 2.81 (t, 1H, *J* = 12.9 Hz, CH_2NCH_2), 2.41 (t, 2H, *J* = 7.4 Hz, CH_2CON), 1.86–1.77 (m, 1H, CH_2CH_2N), 1.75 (quint, 2H, *J* = 7.5 Hz, CH_2CH_2CON), 1.59–1.56 (m, 1H, CH), 1.57–1.48 (m, 2H, , $CH_2CH_2CH_2CON$), 1.24–1.13 (m, 3H, $CH_2CH_2NCH_2CH_2$). ^{13}C NMR (CD_3OD): δ 172.6 (CO), 171.5 (CO), 140.0, 134.9 (C Ar), 132.5 (CH Ar), 130.7, 130.2 (C Ar), 130.0, 129.4, 128.7, 127.2, 126.9, 125.8, 120.8 (CH Ar), 82.6 (C-2), 78.3 (C-3), 70.6 (C-1), 69.7 (C-5), 67.7 (C-4), 59.4, 59.4, 58.8 (CH_3), 56.5 (NCH_2Ph), 48.5 (CH_2NCH_2), 43.2 (CH_2NCH_2), 37.5 (CH_2CON), 35.9 (CH), 35.7 ($CH_2CH_2CH_2CON$), 32.8 (CH_2CH_2N), 31.9 (CH_2CH_2N), 22.8 (CH_2CH_2CON). HRMS (ESI/Q-TOF): *m/z* calcd. for $C_{37}H_{48}N_3O_5$ $[M+H]^+$ 614.3588, found 614.3593.

1-C-(1*S*)-1-[(6-benzamide)-1-hexylamidophenyl-(*N*-benzyl-2,3,5-tri-*O*-methyl)]-1,4-dideoxy-1,4-imino-D-ribitol (18). The iminosugar **10** (40 mg, 0.11 mmol) was allowed to react with the acid **37** (62 mg, 0.22 mmol) as described for the synthesis of **11** to give, after the same work-up and purification (cyclohexane:AcOEt:MeOH, 7:3:0 to 54:36:1), **18** (53.3 mg, 66%) as a colourless oil; $[\alpha]_D$= +23.6 (c 0.7 in $CHCl_3$). ^{1}H NMR (CD_3OD): δ 8.00 (s, 1H, NH), 7.74–7.65 (m, 2H, Ar), 7.52–7.45 (m, 2H, Ar), 7.44–7.25 (m, 5H, Ar), 7.24–7.10 (m, 5H, Ar), 6.59 (t, 1H, *J* = 6.0 Hz, NH), 3.86 (d, 1H, *J* = 8.8 Hz, H-2), 3.70–3.63 (m, 1H, H-1), 3.57–3.43 (m, 4H. H-3, CH_2NH, NCH_2Ph), 3.43–3.34 (m, 3H, H-5, H-5', NCH_2Ph), 3.33 (s, 3H, CH_3), 3.18–3.09 (m, 6H, 2 CH_3), 3.08–2.97 (m, 1H, H-4), 2.31 (t, 2H, *J* = 7.3 Hz, CH_2CO), 1.68 (quint, 2H, *J* = 7.3 Hz, CH_2CH_2NH), 1.56 (quint, 2H, *J* = 7.3 Hz, CH_2CH_2CO), 1.37 (quint, 2H, *J* = 7.3 Hz, $CH_2CH_2CH_2CO$). ^{13}C NMR (CD_3OD): δ 171.9 (CO), 168.1 (CO), 134.6, 131.7 (C Ar), 131.6, 129.9 (CH Ar), 128.8, 128.7 (C Ar), 128.6, 128.3, 127.1, 127.0, 126.19, 120.1 (CH Ar), 84.0 (C-3), 80.4 (C-1), 69.8 (C-2), 65.6 (C-4), 59.2 (C-5), 59.1 (CH_3, CH_2NH), 58.3 (CH_3), 57.3 (CH_3), 39.7 (NCH_2Ph), 37.3 (CH_2CO), 29.1 (CH_2CH_2CO), 26.2 ($CH_2CH_2CH_2CO$), 24.8 (CH_2CH_2NH). HRMS (ESI/Q-TOF): *m/z* calcd. for $C_{34}H_{44}N_3O_5$ $[M+H]^+$ 574.3275, found 574.3277.

1-C-(1*S*)-1-[(((4-(*tert*-butyl)phenyl)sulfonamido)methyl)-benzamide-(*N*-benzyl-2,3,5-tri-*O*-methyl)-phenyl]-1,4-dideoxy-1,4-imino-D-ribitol (19). The iminosugar **10** (31 mg, 0.08 mmol) was allowed to react with the acid **40** (45 mg, 0.12 mmol) as described for the synthesis of **11** to give, after the same work-up and purification (cyclohexane:AcOEt:MeOH, 7:3:0 to 54:36:1), **19** (19 mg, 30%) as an amorphous white solid; $[\alpha]_D$= +9.7 (c 1.0 in $CHCl_3$). ^{1}H NMR (CD_3CN): δ 8.87 (s, 1H, NH), 7.83–7.79 (m, 2H, Ar), 7.79–7.75 (m, 2H, Ar), 7.73–7.69 (m, 2H, Ar), 7.58–7.52 (m, 2H, Ar), 7.51–7.45 (m, 2H, Ar), 7.45–7.39 (m, 5H, Ar), 7.37–7.32 (m, 2H, Ar), 6.17 (t, 1H, *J* = 6.4 Hz, NH), 4.36–4.27 (m, 2H, H-1, NCH_2Ph), 4.19–4.10 (m, 4H, $PhCH_2$, H-2, CH_2), 3.98 (dd, 1H, *J* = 4.3, 1.9 Hz, H-3), 3.76–3.72 (m, 1H, H-4), 3.51 (dd, 1H, *J* = 10.7, 3.6 Hz, H-5), 3.38 (dd, 1H, *J* = 10.7, 4.9 Hz, H-5'), 3.35 (s, 3H, CH_3), 3.34 (s, 3H, CH_3), 3.22 (s, 3H, CH_3), 1.32 (s, 9H, *t*-Bu). ^{13}C NMR (CD_3CN): δ 166.6 (CO), 157.3, 142.6, 141.2, 138.7, 134.8 (C Ar), 132.4, 131.9 (CH Ar), 130.7 (C Ar), 130.6, 129.9, 128.9, 128.6, 127.7, 127.1, 121.6 (CH Ar), 84.5 (C-2), 80.2 (C-3), 71.5 (C-1), 70.8 (C-5), 69.2 (C-4), 59.4 (CH_3), 59.3 (NCH_2Ph), 58.4, 58.1 (CH_3), 47.2 (CH_2), 35.8 (C *t*-Bu), 31.3 (*t*-Bu). HRMS (ESI/Q-TOF): *m/z* calcd. for $C_{39}H_{48}N_3O_6S$ $[M+H]^+$ 686.3258, found 686.3245.

1-C-(1*S*)-1-[4-(*N*-benzyl-2,3,5-tri-*O*-methyl)-(benzamidomethyl)-benzamide(2,3,5-tri-*O*-methyl)-phenyl]-1,4-dideoxy-1,4-imino-D-ribitol (20). The iminosugar **10** (31 mg, 0.08 mmol) was allowed to react with the acid **41** (45 mg, 0.12 mmol) as described for the synthesis of **11** to give, after the same work-up and purification by semi-preparative reverse-phase HPLC (linear gradient H_2O + 0.01% TFA/acetonitrile + 0.01% TFA, 70:30 to 0:100 in 1h), **20** (42 mg, 31%) as a colourless oil; $[\alpha]_D$ = +22.4 (c 0.9 in $CHCl_3$). ^{1}H NMR (CD_3OD): δ 7.98–7.93 (m, 2H, Ar), 7.92–7.88 (m, 2H, Ar), 7.72–7.68 (m, 2H, Ar), 7.61–7.56 (m, 1H, Ar), 7.56–7.48 (m, 6H, Ar), 7.31–7.21 (m, 5H, Ar), 4.69 (s, 2H, CH_2), 3.87 (d, 1H, J = 8.7 Hz, H-2), 3.82 (d, 1H, J = 13.2 Hz, NCH_2Ph), 3.77–3.74 (m, 1H, H-1), 3.60 (dd, 1H, J = 8.7, 4.6 Hz, H-3), 3.48 (d, 1H, J = 13.3 Hz, NCH_2Ph), 3.40 (s, 3H, CH_3), 3.22–3.18 (m, 6H, 2 CH_3), 3.14–3.06 (m, 2H, H-4, H-5), 2.99 (dd, J = 8.6, 3.0 Hz, 1H, H-5'). ^{13}C NMR (CD_3OD): δ 170.3 (CO), 168.6 (CO), 144.5, 139.9, 139.2, 139.2, 135.5, 135.1 (C Ar), 132.9, 130.6, 129.7, 129.5, 129.2, 129.0, 128.6, 128.4, 128.2, 122.3 (CH Ar), 87.8 (C-3), 81.9 (C-1), 75.3 (C-5), 70.9 (C-2), 66.8 (C-4), 59.2 (CH_3), 58.5 (NCH_2Ph), 58.2, 57.2 (CH_3), 44.2 (CH_2). HRMS (ESI/Q-TOF): *m/z* calcd. for $C_{36}H_{40}N_3O_5$ $[M+H]^+$ 594.2962, found 594.2959.

1-C-(1*S*)-1-[(1-(4-*tert*-butylphenyl)sulfonyl)piperidin-4-yl)-butanamidophenyl]-1,4-dideoxy-1,4-imino-D-ribitol (21). A vigorously stirred mixture of **11** (44.5 mg, 0.05 mmol), 20% palladium hydroxide on carbon (20 mg), and acetic acid (2.5 mL) was degassed under vacuum and saturated with hydrogen (by a H_2-filled balloon) three times. The suspension was stirred at room temperature for 24 h under a slightly positive pressure hydrogen (balloon) and followed by HPLC or LCMS. When the reaction was completed, the solution was filtered through a plug of cotton and concentrated. The residue was purified by semi-preparative reverse-phase HPLC (linear gradient H_2O + 0.01% TFA/acetonitrile + 0.01% TFA, 40:60 to 0:100 in 20 min) to obtain the desired compound **21** (11 mg, 40%); $[\alpha]_D$ = -9.4 (*c* 0.45 in CH_3OH). ^{1}H NMR (CD_3OD): δ 7.70–7.63 (m, 6H, Ar), 7.51–7.47 (d, 2H, *J* = 8.6 Hz, Ar), 4.53 (dd, 1H, *J* = 4.5, 9.0 Hz, H-2), 4.50 (d, 1H, *J* = 9.0 Hz, H-1), 4.25 (dd, 1H, *J* = 4.5, 3.1 Hz, H-3), 3.88 (d, 2H, *J* = 4.1 Hz, H-5, H-5'), 3.76–3.69 (m, 3H, H-4, C*H*$_2$NC*H*$_2$), 2.33 (t, 2H, *J* = 7.4 Hz, C*H*$_2$CON), 2.25 (td, 2H, *J* = 11.5, 2.6 Hz, C*H*$_2$NC*H*$_2$), 1.77 (m, 2H, C*H*$_2$CH$_2$NCH$_2$C*H*$_2$), 1.67 (quint, 2H, *J* = 7.54 Hz, *CH*$_2$CH$_2$CON), 1.36 (s, 9H, *t*-Bu), 1.32–1.18 (m, 5H, C*H*$_2$CH$_2$NCH$_2$C*H*$_2$, C*H*). ^{13}C NMR (CD_3OD): δ 174.6 (CO), 157.9, 141.3, 134.5 (C Ar), 130.2 (CH Ar), 129.3 (C Ar), 128.7, 127.2, 121.43 (CH Ar), 75.9 (C-2) , 73.1 (C-3), 67.6 (C-4), 65.6 (C-1), 60.1 (C-5), 47.7 (*C*H$_2$N*C*H$_2$), 37.9 (*C*H$_2$CON), 36.7 (*C*H$_2$CH$_2$CH$_2$CON), 36.1 (CH), 36.0 (C *t*-Bu), 32.7 (*C*H$_2$CH$_2$NCH$_2$*C*H$_2$), 31.45 (*t*-Bu), 23.8 (*C*H$_2$CH$_2$CON). HRMS (ESI/Q-TOF): *m/z* calcd. for $C_{30}H_{44}N_3O_6S$ $[M+H]^+$ 574.2945, found 574.2952.

1-C-(1*S*)-1-[4-(1-benzoylpiperidin-4-yl)-butanamidophenyl]-1,4-dideoxy-1,4-imino-D-ribitol (22). The product **12** (56 mg, 0.07 mmol) was treated as described for the synthesis of **21** to give after 48 h and purification by semi-preparative reverse-phase HPLC (linear gradient H_2O + 0.01% TFA/acetonitrile + 0.01% TFA, 80:20 to 0:100 in 45 min), **22** (10 mg, 34%) as a colourless oil; $[\alpha]_D$ = -8.2 (*c* 0.5 in CH_3OH). ^{1}H NMR (CD_3OD): δ 7.71–7.65 (d, 2H, *J* = 8.7 Hz, Ar), 7.53–7.48 (d, 2H, *J* = 8.7 Hz, Ar), 7.48–7.42 (m, 3H, Ar), 7.40–7.34 (m, 2H, Ar), 4.65–4.58 (m, 1H, C*H*$_2$NCH$_2$), 4.56–4.48 (m, 2H, H-2, H-1), 4.25 (dd, *J* = 4.2, 3.1 Hz, H-3), 3.88 (d, 2H, *J* = 4.1 Hz, H-5, H-5'), 3.74–3.67 (m, 2H, H-4, CH$_2$NC*H*$_2$), 3.15–3.04 (m, 1H, C*H*$_2$NCH$_2$), 2.90–2.78 (m, 1H, CH$_2$NC*H*$_2$), 2.39 (t, 2H, *J* = 7.4 Hz, C*H*$_2$CON), 1.88 (m, 1H, C*H*$_2$CH$_2$NCH$_2$), 1.80–1.57 (m, 3H, CH, C*H*$_2$CH$_2$CON), 1.42–1.07 (m, 5H, C*H*$_2$CH$_2$CH$_2$CON, C*H*$_2$CH$_2$NCH$_2$C*H*$_2$). ^{13}C NMR (CD_3OD): δ 174.7 (CO), 172.4 (CON), 141.4, 137.3 (C Ar), 130.9, 130.2, 129.7 (CH Ar), 129.3 (C Ar), 127.7, 121.4 (CH Ar), 75.93 (C-2), 73.2 (C-3), 67.6 (C-4), 65.6 (C-1), 60.2 (C-5), 48.6 (*C*H$_2$NCH$_2$), 43.7 (CH$_2$N*C*H$_2$), 38.0 (*C*H$_2$CON), 37.0 (CH, *C*H$_2$CH$_2$CH$_2$CON), 36.9 e 33.9 (*C*H$_2$CH$_2$NCH$_2$*C*H$_2$), 33.1 (*C*H$_2$CH$_2$CON). HRMS (ESI/Q-TOF): *m/z* calcd. for $C_{27}H_{36}N_3O_5$ $[M+H]^+$ 482.2649, found 482.2642.

1-C-(1*S*)-1-[(6-benzamide)-1-hexylamidophenyl]-1,4-dideoxy-1,4-imino-D-ribitol (23). The product **13** (18 mg, 0.023 mmol) was treated as described for the synthesis of **21** to give after 32 h and purification by semi-preparative reverse-phase HPLC (linear gradient H_2O + 0.01% TFA/acetonitrile + 0.01% TFA, 95:5 to 0:100 in 1h), **23** (2.8 mg, 17%) as a white solid; $[\alpha]_D$ = -0.2 (*c* 0.2 in CH_3OH). ^{1}H NMR (CD_3OD): δ 7.81 -7.77 (m, 2H, Ar), 7.69–7.65 (m, 2H, Ar), 7.55–7.42 (m, 5H, Ar), 4.54 (dd, 1H, *J* = 9.0, 4.5 Hz, H-2), 4.50 (d, 1H, *J* = 9.0 Hz, H-1), 4.25 (dd, 1H, *J* = 4.5, 3.0 Hz, H-3), 3.88 (d, 2H, *J* = 4.0 Hz, H-5, H-5'), 3.72 –3.69 (m, 1H, H-4), 3.40 (t, 2H, *J* = 7.1 Hz, C*H*$_2$N), 2.41 (t, 2H, *J* = 7.4 Hz, C*H*$_2$CO), 1.76 (quint, 2H, *J* = 7.5 Hz, C*H*$_2$CH$_2$CO), 1.68 (quint, 2H, *J* = 7.3 Hz, C*H*$_2$CH$_2$N), 1.52–1.43 (m, 2H, C*H*$_2$CH$_2$CH$_2$CO). ^{13}C NMR (CD_3OD): δ 174.8 (CO), 170.3 (CO), 141.4, 135.8 (C), 132.6, 130.2, 129.5 (CH), 129.3 (C), 128.2, 121.5 (CH), 75.9 (C-2), 73.2 (C-3), 67.5 (C-4), 65.6 (C-1), 60.2 (C-5), 40.7 (*C*H$_2$N), 37.8 (*C*H$_2$CO), 30.2 (*C*H$_2$CH$_2$N), 27.6 (*C*H$_2$CH$_2$CH$_2$CO), 26.5 (*C*H$_2$CH$_2$CO). HRMS (ESI/Q-TOF): *m/z* calcd. for $C_{24}H_{32}N_3O_5$ $[M+H]^+$ 442.2336, found 442.2336.

1-C-(1*S*)-1-[(1-(4-*tert*-butylphenyl)sulfonamido)methyl)benzamide]-1,4-dideoxy-1,4-imino-D-ribitol (24). The product **14** (40 mg, 0.04 mmol) was treated as described for the synthesis of **21** to give after 32h and purification by semi-preparative reverse-phase HPLC (linear gradient H_2O + 0.01% TFA/acetonitrile + 0.01% TFA 80:20 to 50:50 in 20 min), **24** (6.5 mg, 36%) as an amorphous white solid; $[\alpha]_D$ = -2.7 (*c* 0.7 in CH_3OH). ^{1}H NMR (CD_3OD): δ 7.82 (d, 2H, *J* = 8.5 Hz, Ar), 7.81 (d, 2H, *J* = 8.5 Hz, Ar), 7.72 (d, 2H, *J* = 8.8 Hz, Ar), 7.57 (d, 2H, *J* = 8.5 Hz, Ar), 7.54 (d, 2H, *J* = 8.8 Hz, Ar), 7.37 (d, 2H, *J* = 8.5 Hz, Ar), 4.57 (dd, 1H, *J* = 9.0, 4.4 Hz, H-2), 4.54 (d, 1H, *J* = 9.0 Hz, H-1), 4.27 (dd, 1H, *J* = 4.4, 3.0 Hz, H-3), 4.16 (s, 2H, CH$_2$), 3.90 (d, 2H, *J* = 4.1 Hz, H-5, H-5'), 3.73 (td, 1H, *J* = 4.1, 3.0 Hz, H-4), 1.33 (s, 9H, *t*-Bu). ^{13}C NMR (CD_3OD): δ 168.4 (CO), 157.4, 143.3, 141.3, 139.2, 134.8 (C Ar), 130.2 (CH Ar), 129.9 (C Ar), 129.0, 128.7, 127.9, 127.1, 122.5 (CH Ar), 75.95 (C-2), 73.16 (C-3), 67.61 (C-4), 65.61 (C-1), 60.17 (C-5), 47.42 (*C*H$_2$), 31.47 (t-Bu). HRMS (ESI/Q-TOF): *m/z* calcd. for $C_{29}H_{36}N_3O_6S$ $[M+H]^+$ 554.2325, found 554.2320.

1-C-(1*S*)-1-[4-(benzamidomethyl)-benzamide-phenyl]-1,4-dideoxy-1,4-imino-D-ribitol (25). The product **15** (80 mg, 0.137 mmol) was treated as described for the synthesis of **21** to give after 24h and purification by semi-preparative reverse-phase HPLC (linear gradient H_2O + 0.01% TFA/acetonitrile + 0.01% TFA, 90:10 to 50:50 in 40 min), **25** (60 mg, 54%) as a colourless oil; $[\alpha]_D$ = -17.7 (*c* 0.6 in CH_3OH). ^{1}H NMR (CD_3OD): δ 7.93 (d, 2H, *J* = 8.3 Hz, Ar), 7.90–7.86 (m, 2H, Ar), 7.84 (d, 2H, *J* = 8.6 Hz, Ar), 7.56 (d, 2H, *J* = 8.6 Hz, Ar), 7.56–7.54 (m, 1H, Ar), 7.51 (d, 2H, *J* = 8.3 Hz, Ar), 7.51–7.46 (m, 3H, Ar), 4.67 (s, 2H, CH$_2$), 4.57 (dd, 1H, *J* = 9.0, 4.4 Hz, H-2), 4.54 (d, *J* = 9.0 Hz, H-1), 4.26 (dd, 1H, *J* = 4.4, 3.0 Hz, H-3), 3.90 (d, 2H, *J* = 4.1 Hz, H-5, H-5'), 3.74–3.70 (m, 1H, H-4). ^{13}C NMR (CD_3OD): δ 170.3 (CO), 168.7 (CO), 144.8, 141.4, 135.4, 134.8 (C), 132.9, 130.2 (CH), 129.9 (C), 129.7, 129.0, 128.6, 128.3, 122.5 (CH), 76.0 (C-2), 73.2 (C-3), 67.6 (C-4), 65.6 (C-1), 60.2 (C-5), 44.2 (CH$_2$). HRMS (ESI/Q-TOF): *m/z* calcd. for $C_{26}H_{28}N_3O_5$ $[M+H]^+$ 462.2029, found 462.2031.

1-C-(1*S*)-1-[(1-(4-*tert*-butylphenyl)sulfonyl)piperidin-4-yl)-(2,3,5-tri-*O*-methyl)butanamido phenyl]-1,4-dideoxy-1,4-imino-D-ribitol (26). A vigorously stirred mixture of **16** (44.5 mg, 0.05 mmol), 20% palladium hydroxide on carbon (15 mg), and methanol (2.5 mL) was degassed under vacuum and saturated with hydrogen (by a H_2-filled balloon) three times. The suspension was stirred at room temperature for 2 h under a slightly positive pressure of hydrogen (balloon) and followed by HPLC or LCMS. When the reaction was completed, the solution was filtered through a plug of cotton and concentrated. The residue was purified by semi-preparative reverse-phase HPLC (linear gradient H_2O + 0.01% TFA/acetonitrile + 0.01% TFA, 50:50 to 0:100 in 20 min) to obtain the desired compound **26** as a colourless oil (8.2 mg, 37%); $[\alpha]_D$ = -22.7 (*c* 0.75 in CH_3OH). ^{1}H NMR (CD_3OD): δ 7.71–7.63 (m, 6H, H Ar), 7.45 (d, 2H, *J* = 8.7 Hz, H Ar), 4.56 (d, 1H, *J* = 9.4 Hz, H-1), 4.36 (dd,

1H, J = 9.4, 4.4 Hz, H-2), 4.16 (dd, 1H, J = 4.4, 2.4 Hz, H-3), 3.97–3.94 (m, 1H, H-4), 3.77–3.70 (m, 3H, H-5, CH_2NCH_2), 3.68 (dd, 1H, J = 10.8, 3.4 Hz, H-5'), 3.53 (s, 3H, CH_3), 3.46 (s, 3H, CH_3), 3.39 (s, 3H, CH_3), 2.34 (t, 2H, J = 7.5 Hz, CH_2CON), 2.24 (m, 2H, CH_2NCH_2), 1.78 (m, 2H, $CH_2CH_2NCH_2CH_2$), 1.72–1.63 (m, 2H, CH_2CH_2CON), 1.36 (s, 9H, *t*-Bu), 1.32–1.17 (m, 5H, $CH_2CH_2NCH_2CH_2$, $CH_2CH_2CH_2CON$, CH). ^{13}C NMR (CD_3OD): δ 174.7 (CO), 157.9, 141.6, 134.4 (C Ar), 130.2 (CH Ar), 128.9 (C Ar), 128.8 (CH Ar), 127.2 (CH Ar), 121.4 (CH Ar), 84.2 (C-2), 80.5 (C-3), 70.5 (C-5), 64.3 (C-1), 63.6 (C-4), 59.5 (CH_3), 58.8 (CH_3), 58.6 (CH_3), 47.7 (CH_2NCH_2), 37.9 (CH_2CON), 36.7 ($CH_2CH_2CH_2OH$), 36.1 (CH), 36.1 (C), 32.7 ($CH_2CH_2NCH_2CH_2$), 31.4 (*t*-Bu), 23.8 (CH_2CH_2OH). HRMS (ESI/Q-TOF): *m/z* calcd. for $C_{33}H_{50}N_3O_6S$ $[M+H]^+$ 616.3415, found 616.3399.

1-C-(1*S*)-1-[(4-(1-benzoylpiperidin-4-yl)-butanamidophenyl)-(2,3,5-tri-*O*-methyl)]-1,4-dideoxy-1,4-imino-D-ribitol (27). The product **17** (11 mg, 0.02 mmol) was treated as described for the synthesis of **26**. Purification by semi-preparative reverse-phase HPLC (linear gradient H_2O + 0.01% TFA/acetonitrile + 0.01% TFA, 60:40 to 0:100 in 20 min), **27** (4.2 mg, 50%) as a colourless oil; $[\alpha]_D$ = -24.9 (*c* 0.42 in CH_3OH). ^1H NMR (CD_3OD): δ 7.73–7.67 (m, 2H, Ar), 7.50–7.42 (m, 5H, Ar), 7.42–7.35 (m, 2H, Ar), 4.62 (d, 1H, J = 12.7 Hz, CH_2NCH_2), 4.56 (d, 1H, J = 9.4 Hz, H-1), 4.36 (dd, 1H, J = 9.4, 4.4 Hz, H-2), 4.17 (dd, 1H, J = 4.4, 2.4 Hz, H-3), 3.96 (td, 1H, J = 3.7, 2.4 Hz, H-4), 3.78–3.65 (m, 3H, H-5, H-5', CH_2NCH_2), 3.53 (s, 3H, CH_3), 3.46 (s, 3H, CH_3), 3.39 (s, 3H, CH_3), 3.09 (t, J = 13.0 Hz, CH_2NCH_2), 2.84 (t, 1H, J = 12.7 Hz, CH_2NCH_2), 2.40 (t, 2H, J = 7.4 Hz, CH_2CON), 1.91–1.83 (m, 1H, $CH_2CH_2NCH_2CH_2$), 1.74 (quint, 2H, CH_2CH_2CON), 1.73–1.67 (m, 1H, $CH_2CH_2NCH_2CH_2$), 1.67–1.58 (m, 1H, CH), 1.41–1.31 (m, 2H, $CH_2CH_2CH_2CON$), 1.26–1.09 (m, 2H, $CH_2CH_2NCH_2CH_2$). ^{13}C NMR (CD_3OD): δ 173.3 (CO), 171.0 (CO), 140.2,135.9, 129.5 (C Ar), 128.8, 128.31, 127.4, 126.34, 120.06 (CH Ar), 82.8 (C-2), 79.1 (C-3), 69.1 (C-5), 62.5 (C-1), 62.2 (C-4), 58.1, 57.4, 57.1 (CH_3), 42.3, 36.6 (2 CH_2NCH_2), 35.6 (CH_2CON), 35.5 (CH, $CH_2CH_2CH_2CON$), 32.4, 31.6 (2 $CH_2CH_2NCH_2CH_2$), 22.46 (CH_2CH_2CON). HRMS (ESI/Q-TOF): *m/z* calcd. for $C_{30}H_{42}N_3O_5$ $[M+H]^+$ 524.3119, found 524.3110.

1-C-(1*S*)-1-[(6-benzamide)-1-hexylamidophenyl-(2,3,5-tri-*O*-methyl)]-1,4-dideoxy-1,4-imino-D-ribitol (28). The product **18** (43.3 mg, 0.08 mmol) was treated as described for the synthesis of **26**. Purification by semi-preparative reverse-phase HPLC (linear gradient H_2O + 0.01% TFA/acetonitrile + 0.01% TFA, 50:50 to 0:100 in 20 min), **28** (24.4 mg, 74%) as a colourless oil; $[\alpha]_D$ = -27.9 (*c* 1.0 in CH_3OH). ^1H NMR (CD_3OD): δ 7.82–7.77 (m, 2H), 7.72–7.66 (m, 2H), 7.55–7.49 (m, 1H), 7.48–7.40 (m, 4H), 4.57 (d, 1H, J = 9.3 Hz, H-1), 4.36 (dd, 1H, J = 9.4, 4.4 Hz, H-2), 4.16 (dd, J = 4.4, 2.5 Hz, H-3), 3.99–3.94 (m, 1H, H-4), 3.75 (dd, 1H, J = 10.8, 4.1 Hz, H-5), 3.69 (dd, 1H, J = 10.8, 3.4 Hz, H-5'), 3.53 (s, 3H, CH_3), 3.46 (s, 3H, CH_3), 3.39 (t, 2H, J = 7.4 Hz, CH_2NCO), 3.38 (s, 3H, CH_3), 2.42 (t, 2H, J = 7.4 Hz, CH_2CON), 1.76 (quint, 2H, J = 7.4 Hz, CH_2CH_2CON), 1.71–1.61 (m, 2H, CH_2CH_2NCO), 1.52–1.40 (m, 2H, $CH_2CH_2CH_2NCO$). ^{13}C NMR (CD_3OD): δ 174.76 (CO), 170.27 (CO), 141.59, 135.84 (C Ar), 132.54, 130.14, 129.52 (CH Ar), 128.86 (C Ar), 128.21, 121.53 (CH Ar), 84.15 (C-2), 80.48 (C-3), 70.48 (C-5), 64.34 (C-1), 63.61 (C-4), 59.53 (CH_3), 58.83 (CH_3), 58.56 (CH_3), 40.76 (CH_2NCO), 37.82 (CH_2CON), 30.21 (CH_2CH_2CON), 27.59 (CH_2CH_2NCO), 26.43 ($CH_2CH_2CH_2CON$). HRMS (ESI/Q-TOF): *m/z* calcd. for $C_{27}H_{38}N_3O_5$ $[M+H]^+$ 484.2806, found 484.2806.

1-C-(1*S*)-1-[(((4-(*tert*-butyl)phenyl)sulfonamido)methyl)-benzamide-(2,3,5-tri-*O*-methyl)-phenyl]-1,4-dideoxy-1,4-imino-D-ribitol (29). The product **19** (18 mg, 0.03 mmol) was treated as described for the synthesis of **26**. Purification by semi-preparative reverse-phase HPLC (linear gradient H_2O + 0.01% TFA/acetonitrile + 0.01% TFA, 60:40 to 0:100 in 20 min), **29** (3.5 mg, 20%) as an amorphous white solid; $[\alpha]_D$ = -5.9 (*c* 0.15 in CH_3OH). ^1H NMR (CD_3OD): δ 7.86–7.80 (m, 4H, H Ar), 7.75–7.70 (m, 2H, H Ar), 7.57–7.50 (m, 4H, H Ar), 7.39–7.35 (m, 2H, H Ar), 4.61 (d, 1H, J

= 9.3 Hz, H-1), 4.40 (dd, 1H, J = 9.3, 4.4 Hz, H-2), 4.18 (dd, 1H, J = 4.4, 2.5 Hz, H-3), 4.16 (s, 2H, CH2), 4.00–3.96 (m, 1H, H-4), 3.77 (dd, 1H, J = 10.8, 3.4 Hz, H-5), 3.70 (dd, 1H, J = 10.8, 3.4 Hz, H-5'), 3.55 (s, 3H, CH_3), 3.48 (s, 3H, CH_3), 3.41 (s, 3H, CH_3), 1.34 (s, 9H, *t*-Bu). ^{13}C NMR (CD_3OD): δ 168.5 (CO), 157.5, 143.3, 141.6, 139.2, 134.8 (C Ar), 130.1 (CH Ar), 129.4 (C Ar), 129.0, 128.8, 127.9, 127.1, 122.6 (CH Ar), 84.2 (C-2), 80.5 (C-3), 70.5 (C-5), 64.4 (C-1), 63.7 (C-4), 59.6, 58.9, 58.6 (CH_3), 47.4 (CH_2), 36.0 (C t-Bu), 31.47 (*t*-Bu). HRMS (ESI/Q-TOF): *m/z* calcd. for $C_{32}H_{42}N_3O_6S$ $[M+H]^+$ 596.2789, found 596.2784.

1-C-(1*S*)-1-[4-(benzamidomethyl)-benzamide-(2,3,5-tri-*O*-methyl)-phenyl]-1,4-dideoxy-1,4-imino-D-ribitol (30). The product **20** (29.8 mg, 0.05 mmol) was treated as described for the synthesis of **26**. Purification by semi-preparative reverse-phase HPLC (linear gradient H_2O + 0.01% TFA/acetonitrile + 0.01% TFA, 60:40 to 0:100 in 15 min), **30** (24 mg, 96%) as a colourless oil; $[\alpha]_D$ = -24.9 (*c* 1.14 in CH_3OH). ^1H NMR (CD_3OD): δ 7.96–7.90 (m, 2H, Ar), 7.90–7.82 (m, 4H, Ar), 7.58–7.44 (m, 7H, Ar), 4.66 (s, 2H, CH_2), 4.60 (d, 1H, J = 9.3 Hz, H-1), 4.39 (dd, 1H, J = 9.3, 4.4 Hz, H-2), 4.18 (dd, 1H, J = 4.4, 2.5 Hz, H-3), 4.00–3.95 (m, 1H, H-4), 3.76 (dd, 1H, J = 10.8, 4.0 Hz, H-5), 3.70 (dd, 1H, J = 10.8, 3.4 Hz, H-5'), 3.54 (s, 3H, CH_3), 3.47 (s, 3H, CH_3), 3.41 (s, 3H, CH_3). ^{13}C NMR (CD_3OD): δ 144.8 (CO), 141.6 (CO), 135.4, 134.7, 132.9, 132.9 (C Ar), 130.1, 129.6 (CH Ar), 129.4 (C Ar), 129.0, 128.6, 128.3, 122.6 (CH Ar), 84.2 (C-2), 80.5 (C-3), 70.5 (C-5), 64.4 (C-1), 63.7 (C-4), 59.5, 58.8, 58.6 (CH_3), 44.19 (CH_2). HRMS (ESI/Q-TOF): *m/z* calcd. for $C_{29}H_{34}N_3O_5$ $[M+H]^+$ 504.2493, found 504.2497.

4-(1-((4-*tert*-butylphenyl)sulfonyl)piperidin-4-yl)butanoic acid (32). 4-(Piperidin-4-yl)butanoic acid (300 mg, 1.75 mmol), potassium carbonate (726 mg, 5.26 mmol) were dissolved in THF (1.5 mL) and H_2O (1.5 mL), and the solution was cooled to 0 °C. 4-*tert*-butylbenzenesulfonyl chloride (530 mg, 2.28 mmol) was added and the solution was warmed to room temperature and stirred for 16 h. The reaction was diluted with H_2O (30 mL) and the aqueous layer was washed with AcOEt (30 mL), and the organic layer was discarded. The aqueous layer was acidified with 1 M HCl to pH 1-2 and the solution was extracted with AcOEt (30 mL) for three times. The organic fractions were combined, washed with brine (50 mL), dried (Na_2SO_4), and concentrated. The crude was purified by semi-preparative reverse-phase HPLC (linear gradient H_2O + 0.01% TFA/acetonitrile + 0.01 %TFA, 60:40 to 0:100 in 30 min) to give **32** as a white solid (447 mg, 70% yield). ^1H NMR (DMSO-d6): δ 7.70–7.64 (m, 2H, Ar), 7.55–7.49 (m, 2H, Ar), 3.84-3.75 (m, 2H, CH_2NCH_2), 2.30 (t, 2H, J = 7.4 Hz, CH_2CO_2H), 2.23 (td, 2H, J = 11.8, 2.5 Hz, CH_2NCH_2), 1.77–1.67 (m, 2H, $CH_2CH_2NCH_2CH_2$), 1.66–1.55 (m, 3H, $CH_2CH_2CO_2H$, CH), 1.35 (s, 9H, *t*-Bu), 1.33–1.13 (m, 4H, $CH_2CH_2NCH_2CH_2$, $CH_2CH_2CH_2CO_2H$). ^{13}C NMR (DMSO-d6): δ 178.6 (CO), 156.4, 133.9 (C), 127.7, 126.0 (CH Ar), 46.5 (CH_2NCH_2), 35.5 ($CH_2CH_2CO_2H$), 35.0 (C *t*-Bu), 33.8 (CH_2CO_2H), 31.5 ($CH_2CH_2NCH_2CH_2$), 31.2 (CH), 21.8 ($CH_2CH_2CH_2CO_2H$). HRMS (ESI/Q-TOF): *m/z* calcd. for $C_{19}H_{28}NO_4S$ $[M-H]^-$ 366.1745, found 366.1752.

4-(1-Benzoylpiperidin-4-yl)butanoic (33). 4-(Piperidin-4-yl)butanoic acid (100 mg, 0.54 mmol), potassium carbonate (242 mg, 1.6 mmol), were dissolved in THF (1.5 mL) and H_2O (1.5 mL), and the solution was cooled to 0 °C. Benzoyl chloride (0.68 mL, 0.54 mmol) was added and the solution was warmed to room temperature and stirred for 16 h. The reaction was diluted with H_2O (10 mL) and the aqueous layer was washed with AcOEt (10 mL), and the organic layer was discarded. The aqueous layer was acidified with 1 M HCl to pH 1-2 and the solution was extracted with AcOEt (15 mL) for three times. The organic fractions were combined, washed with brine (30 mL), dried (Na_2SO_4), and concentrated. The crude was purified by semi-preparative reverse-phase HPLC (linear gradient H_2O + 0.01% TFA/acetonitrile + 0.01% TFA, 60:40 to 0:100 in 30 min) to give the product **33** as a white solid (96 mg, 62%). ^1H NMR (DMSO-d6): δ 7.44–7.39 (m, 3H, Ar), 7.38–7.32 (m, 2H,

Ar), 4.08–3.98 (m, 2H, CH_2NCH_2), 2.95–2.88 (m, 2H, CH_2NCH_2), 2.21 (t, 2H, J = 7.3 Hz, CH_2CO_2H), 1.74–1.65 (m, 2H, $CH_2CH_2NCH_2CH_2$), 1.64–1.50 (m, 3H, $CH_2CH_2NCH_2CH_2$, CH), 1.36–1.26 (m, 2H, $CH_2CH_2CO_2H$), 1.18–1.06 (m, 2H, $CH_2CH_2CH_2CO_2H$). ^{13}C NMR (DMSO-d6): δ 173.2 (COOH), 136.3 (CO), 128.4 (C), 127.5, 125.9 (CH Ar), 43.8 (CH_2NCH_2), 34.5 ($CH_2CH_2CO_2H$), 34.5 (CH), 33.3 (CH_2CO_2H), 31.2 ($CH_2CH_2NCH_2CH_2$), 21.1 ($CH_2CH_2CH_2CO_2H$). HRMS (ESI/Q-TOF): *m/z* calcd. for $C_{16}H_{21}NO_3$ [M-H]$^-$ 276.1600, found 276.1594.

4-(1-((4-(*tert*-butyl)phenyl)sulfonyl)piperidin-4-yl)-*N*-phenylbutanamide (34). A solution of aniline (50 mg, 0.12 mmol), **32** (280 mg, 0.18 mmol) and PyBOP (475 mg, 0.2 mmol) in dry DMF (1.5 mL) under argon, was cooled to 0 °C. *i*-Pr_2NEt (0.04 mL, 0.24 mmol) was slowly added at 0 °C, and the reaction mixture was stirred at room temperature for 16 h. The solvent was removed and purified by flash column chromatography (cyclohexane:AcOEt, 10:0 to 5:5) to afford **34** (218 mg, 91%) as an amorphous white solid. ^{1}H NMR (DMSO-d6): δ 9.80 (s, 1H, NH), 7.65 (s, 4H, Ar), 7.59–7.52 (m, 2H, Ar), 7.28 –7.24 (m, 2H, Ar), 7.03–6.96 (m, 1H, Ar), 3.66–3.58 (m, 2H, CH_2NCH_2), 2.23 (t, 2H, J = 7.4 Hz, CH_2CON), 2.20–2.10 (m, 2H, CH_2NCH_2), 1.73–1.69 (m, 2H, $CH_2CH_2NCH_2CH_2$), 1.54 (m, 2H, CH_2CH_2CON), 1.31 (s, 9H, *t*-Bu), 1.23–1.03 (m, 5H, CH, $CH_2CH_2NCH_2CH_2$, $CH_2CH_2CH_2CON$). ^{13}C NMR (DMSO-d6): δ 171.2 (CONH), 155.9, 139.3, 132.7 (C Ar), 128.6, 127.4, 126.1, 122.9, 119.0 (CH Ar), 46.1 (CH_2NCH_2), 36.4 (CH_2CON), 35.1 (CH_2CH_2CON), 34.9 ($CH_2CH_2NCH_2CH_2$), 33.9 (CH), 30.9 (C t-Bu), 30.8 (*t*-Bu), 22.2 ($CH_2CH_2CH_2CON$). HRMS (ESI/Q-TOF): *m/z* calcd. for $C_{25}H_{35}N_2O_3S$ [M+H]$^+$ 443.2368, found 443.2369.

4-(1-Benzoylpiperidin-4-yl)-*N*-phenylbutanamide (35). The acid **33** (0.25 mmol) was dissolved in dichloroethane (3 mL), and $SOCl_2$ (3.3 mmol) was added. The reaction was stirred at room temperature for 3 h. Then solvent was removed, and the residue dissolved in dry CH_2Cl_2 (2 mL). The solution was then cooled to 0 °C and aniline (1 mmol) and Et_3N (1.5 mmol) were added, and the mixture stirred at room temperature for 2.5 h. The residue was purified by flash column chromatography (cyclohexane:AcOEt, 5:5) to give **35** (75.8 mg, 85%) as a white solid. ^{1}H NMR (DMSO-d6): δ 9.45 (s, 1H, NH), 7.59–7.54 (m, 2H, Ar), 7.44–7.40 (m, 3H, Ar), 7.39–7.32 (m, 2H, Ar), 7.29–7.24 (m, 2H, Ar), 7.04–6.99 (m, 1H, Ar), 4.06–3.94 (m, 2H, CH_2NCH_2), 2.95–2.86 (m, 2H, CH_2NCH_2), 2.31 (t, 2H, J = 7.3 Hz, CH_2CO_2H), 1.75–1.63 (m, 4H, $CH_2CH_2NCH_2CH_2$), 1.62–1.53 (m, 1H, CH), 1.37–1.30 (m, 2H, $CH_2CH_2CO_2H$), 1.17–1.09 (m, 2H, $CH_2CH_2CH_2CO_2H$). ^{13}C NMR (DMSO-d6): δ 170.5 (CO), 168.5 (CO), 138.8, 136.3 (C Ar), 128.4, 127.8, 127.6, 125.9, 122.3, 119.0 (CH Ar), 44.3 (CH_2NCH_2), 36.1 (CH_2CON), 34.7 (CH_2CH_2CON), 34.6 (CH), 30.5 ($CH_2CH_2NCH_2CH_2$), 22.1 ($CH_2CH_2CH_2CON$). HRMS (ESI/Q-TOF): *m/z* calcd. for $C_{22}H_{27}N_2O_2$ [M+H]$^+$ 351.2073, found 351.2073.

6-(Benzoylamino)hexanoic acid (37). To a solution of 6-aminohexanoic acid (0.15 g, 1.14 mmol) in aqueous 10% NaOH (5 mL) benzoyl chloride (0.4 mL, 3.4 mmol) was slowly added. The mixture was stirred at room temperature for 6 h, then acidified until pH 1 with 1 M aqueous HCl and extracted three times with AcOEt (50 mL). The organic phase was dried (Na_2SO_4), filtered and concentrated under reduced pressure. The residue was purified by flash column chromatography (cyclohexane:AcOEt, 9:1 to 1:1 + 1% AcOH) to give the known [34] acid **37** (0.19 g, 71%) as an amorphous white solid. ^{1}H NMR ($CDCl_3$): δ 10.41 (br s, 1H, NH), 7.85–7.61 (m, 2H, Ar), 7.52–7.29 (m, 3H, Ar), 6.27 (br s, 1H, NH), 3.45 (q, 2H, J = 6.0 Hz, CH_2CO_2H), 2.34 (t, 2H, J = 7.0 Hz, CH_2NCO), 1.18-1.85 (m, 6H, $CH_2CH_2CH_2$).

***N*-(6-oxo-6-(phenylamino)hexyl)benzamide (38)**. The same procedure described for the synthesis of **34** was applied to the acid **37** to give the amide **38**. Column chromatography (CH_2Cl_2:cyclohexane:AcOEt, 30:70:0 to 25:62:13). Yield: 51%, amorphous white solid. ^{1}H NMR (DMSO-d6): δ 9.84 (s, 1H, NH), 8.44 (t, 1H, J = 5.7 Hz, NH), 7.87–7.77 (m, 2H, Ar), 7.61–7.54 (m,

2H, Ar), 7.54–7.47 (m, 1H, Ar), 7.47–7.40 (m, 2H, Ar), 7.32–7.21 (m, 2H, Ar), 7.05–6.96 (m, 1H, Ar), 3.29–3.21 (m, 2H, C*H*$_2$NHCO), 2.31 (t, 2H, *J* = 7.4 Hz, C*H*$_2$CO), 1.67–1.57 (quint, 2H, *J* = 7.4 Hz, NHCH$_2$C*H*$_2$), 1.59–1.50 (quint, 2H, *J* = 7.4 Hz, COCH$_2$C*H*$_2$), 1.39–1.29 (m, 2H, COCH$_2$CH$_2$C*H*$_2$). ^{13}C NMR (DMSO-d6): δ 171.2, 166.1 (CO), 139.3, 134.7 (C Ar), 130.9, 128.6, 128.2, 127.1, 122.9, 119.0 (CH Ar), 39.1, 36.3, 28.9, 26.1, 24.9 (CH$_2$). HRMS (ESI/Q-TOF): *m/z* calcd. for $C_{19}H_{23}N_2O_2$ [M+H]$^+$ 311.1760, found 311.1760.

4-(((4-(*tert*-butyl)phenyl)sulfonamido)methyl)benzoic acid (40). To a solution of 4-(aminomethyl)benzoic acid (0.3 g, 1.98 mmol) in H_2O (3 mL) 4-(*tert*-butyl)benzenesulfonyl chloride (0.5 g, 2.2 mmol) and $Na_2B_4O_7$ (1.2 g, 6.3 mmol) were added. The mixture stirred at 80 °C overnight, then diluted with H_2O (50 mL), acidified until pH 1 with 1 M aqueous HCl, and extracted three times with AcOEt (50 mL). The organic phase was dried (Na_2SO_4), filtered and concentrated. The residue was purified by flash column chromatography (1:1 cyclohexane:AcOEt, containing 1% of AcOH) to give the known [35] acid **40** (0.65 g, 94%) as a white solid. ^{1}H NMR (DMSO-d6): δ 8.20 (t, 1H, *J* = 6.4 Hz, NH), 7.81–7.75 (d, 2H, *J* = 8.2 Hz, Ar), 7.68–7.63 (d, 2H, *J* = 8.5 Hz, Ar), 7.55–7.49 (d, 2H, *J* = 8.5 Hz, Ar), 7.30 (d, 2H, *J* = 8.2 Hz, Ar), 4.07 (d, 2H, *J* = 6.4 Hz, CH$_2$), 1.28 (s, 9H, *t*-Bu).

4-(Benzamidomethyl)benzoic acid (41). A suspension of 4-(aminomethyl)benzoic acid (0.300 g, 1.98 mmol) in dry CH_2Cl_2 (2 mL) and dry DMF (0.5 mL) was cooled to 0 °C, then benzoyl chloride (0.25 mL, 1.98 mmol) and *i*-Pr$_2$NEt (0.04 mL, 0.24 mmol) were slowly added in sequence at 0 °C. The reaction mixture was stirred at room temperature for 16 h, then diluted with AcOEt (300 mL) and washed with 1 M HCl (200 mL). The aqueous phase was extracted with AcOEt (2 x 200 mL). The combined organic phases were dried (Na_2SO_4), filtered and concentrated under reduced pressure. The residue was purified by flash column chromatography (cyclohexane:AcOEt, 7:3 to 1:1 + 1% AcOH) to give the known [36] acid **41** (0.460 g, 91%) as a white solid. ^{1}H NMR (DMSO-d6): δ 9.12 (t, 1H, *J* = 6.0 Hz, NH), 7.94–7.87 (m, 4H, Ar), 7.58–7.52 (m, 1H, Ar), 7.52–7.45 (m, 2H, Ar), 7.45–7.40 (m, 2H, Ar), 4.55 (d, 2H, *J* = 6.0 Hz, CH$_2$).

4-(((4-(tert-butyl)phenyl)sulfonamido)methyl)-*N*-phenylbenzamide (42). The same procedure described for the synthesis of **34** was applied to the acid **40** to give the amide **42**. Column chromatography (CH_2Cl_2:cyclohexane:AcOEt, from 30:70:0 to 25:62:13). Yield: 10%, amorphous white solid. ^{1}H NMR (DMSO-d6): δ 9.35 (s, 1H, NH), 7.92–7.87 (m, 2H, Ar), 7.83–7.78 (m, 4H, Ar), 7.62–7.57 (m, 2H, Ar), 7.43–7.38 (m, 2H, Ar), 7.35–7.28 (m, 2H, Ar), 7.13–7.04 (m, 2H, Ar, NH), 4.16 (d, 2H, *J* = 6.3 Hz, CH$_2$), 1.39 (s, 9H, *t*-Bu). ^{13}C NMR (DMSO-d6): δ 164.1 (CO), 153.8, 141.45, 139.7, 138.9, 134.7 (C Ar), 129.0, 127.4, 127.3, 126.7, 125.7, 123.1, 119.7 (CH Ar), 46.4 (CH$_2$), 34.8 (C *t*-Bu), 30.4 (*t*-Bu). HRMS (ESI/Q-TOF): *m/z* calcd. for $C_{24}H_{26}N_2O_3S$ [M+H]$^+$ 423.1737, found 423.1745.

4-(Benzamidomethyl)-*N*-phenylbenzamide (43). The same procedure described for the synthesis of **35** was applied to the acid **41** to give the amide **43**. Column chromatography (cyclohexane:AcOEt, from 7:3 to 4:6). Yield: 92%, amorphous white solid. ^{1}H NMR (DMSO-d6): δ 10.18 (s, 1H, NH), 9.13 (t, *J* = 6.0 Hz, NH), 7.95–7.88 (m, 4H, Ar), 7.80–7.74 (m, 2H, Ar), 7.58–7.53 (m, 1H, Ar), 7.52–7.44 (m, 4H, Ar), 7.37–7.31 (m, 2H, Ar), 7.09 (tt, 1H, *J* = 7.4, 1.2 Hz, Ar), 4.56 (d, 2H, *J* = 6.0 Hz, CH$_2$). ^{13}C NMR (DMSO-d6): δ 166.3 (CO), 165.3 (CO), 143.4, 139.2, 134.2, 133.4 (C Ar), 131.3, 128.6, 128.3, 127.7, 127.2, 127.0, 123.6, 120.3 (CH Ar), 42.4 (CH$_2$). HRMS (ESI/Q-TOF): *m/z* calcd. for $C_{21}H_{19}N_2O_2$ [M+H]$^+$ 331.1441, found 331.1444.

Compound **7**

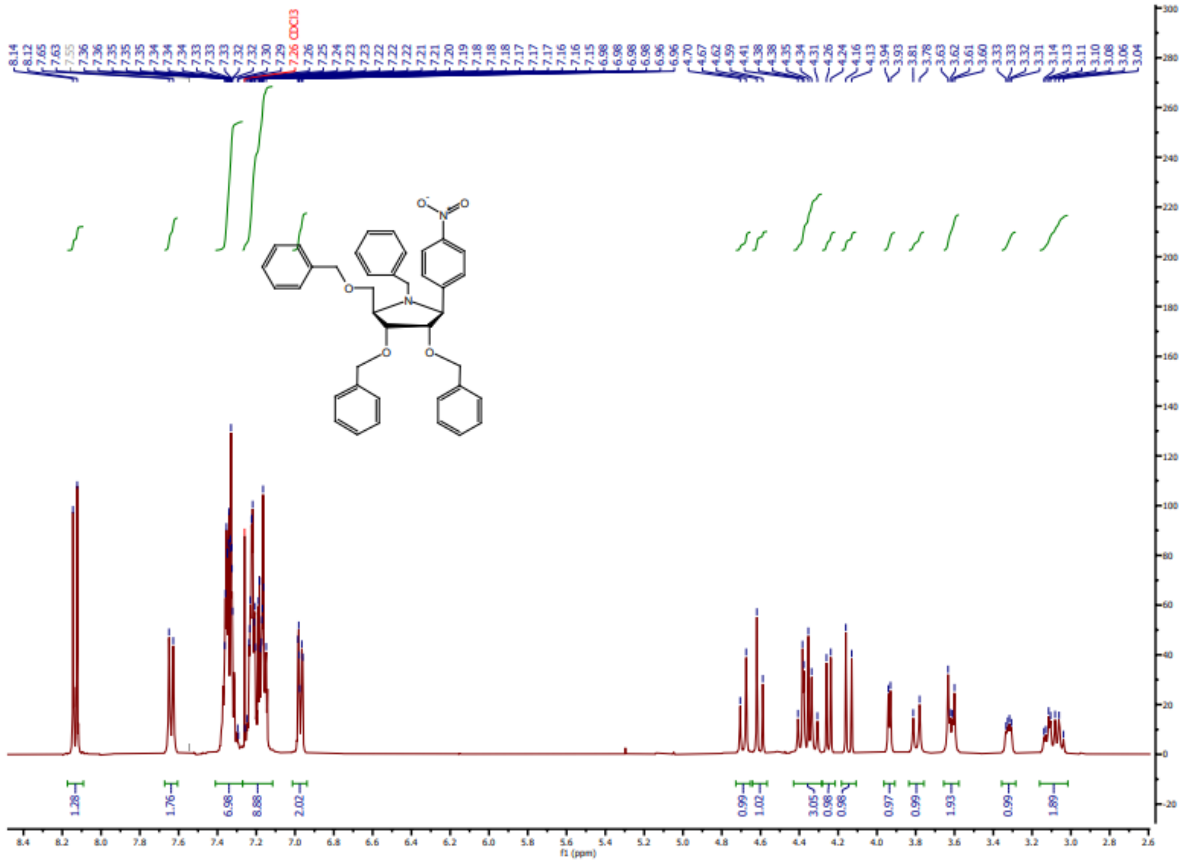

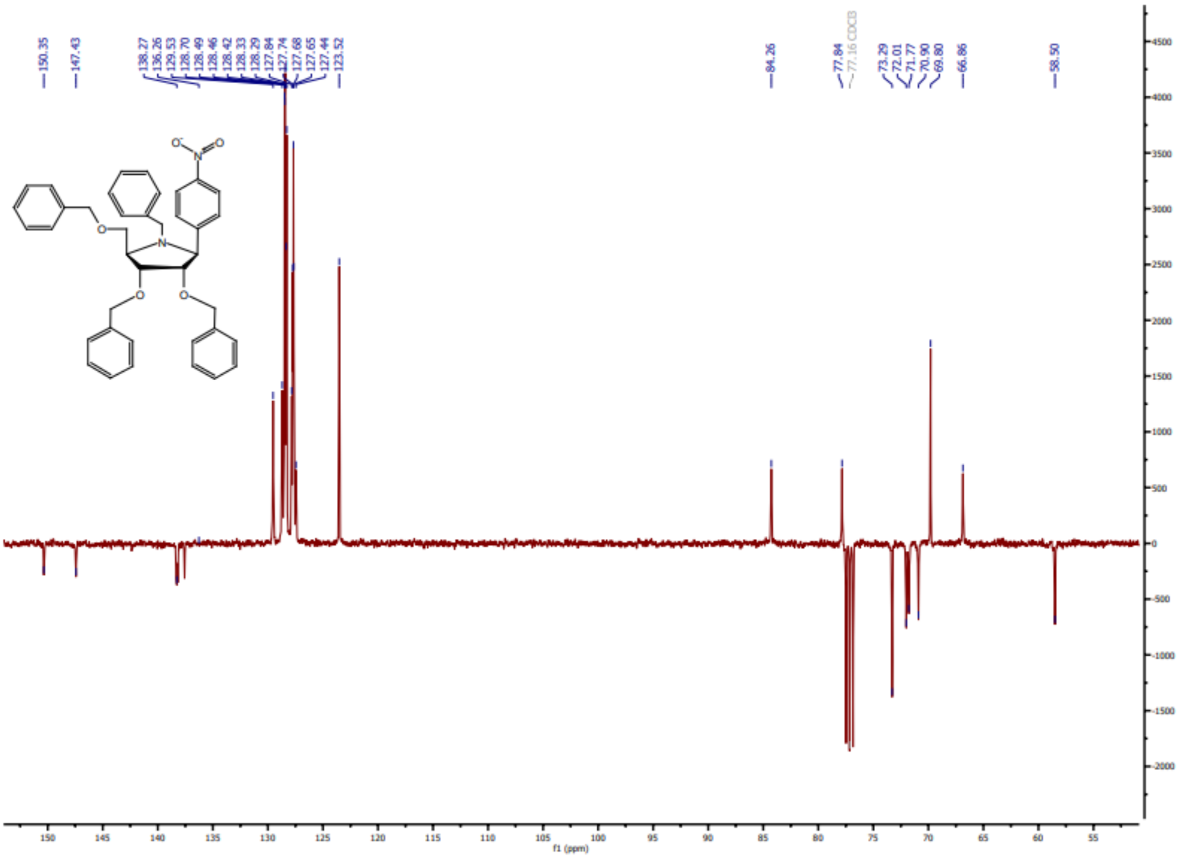

Compound **8**

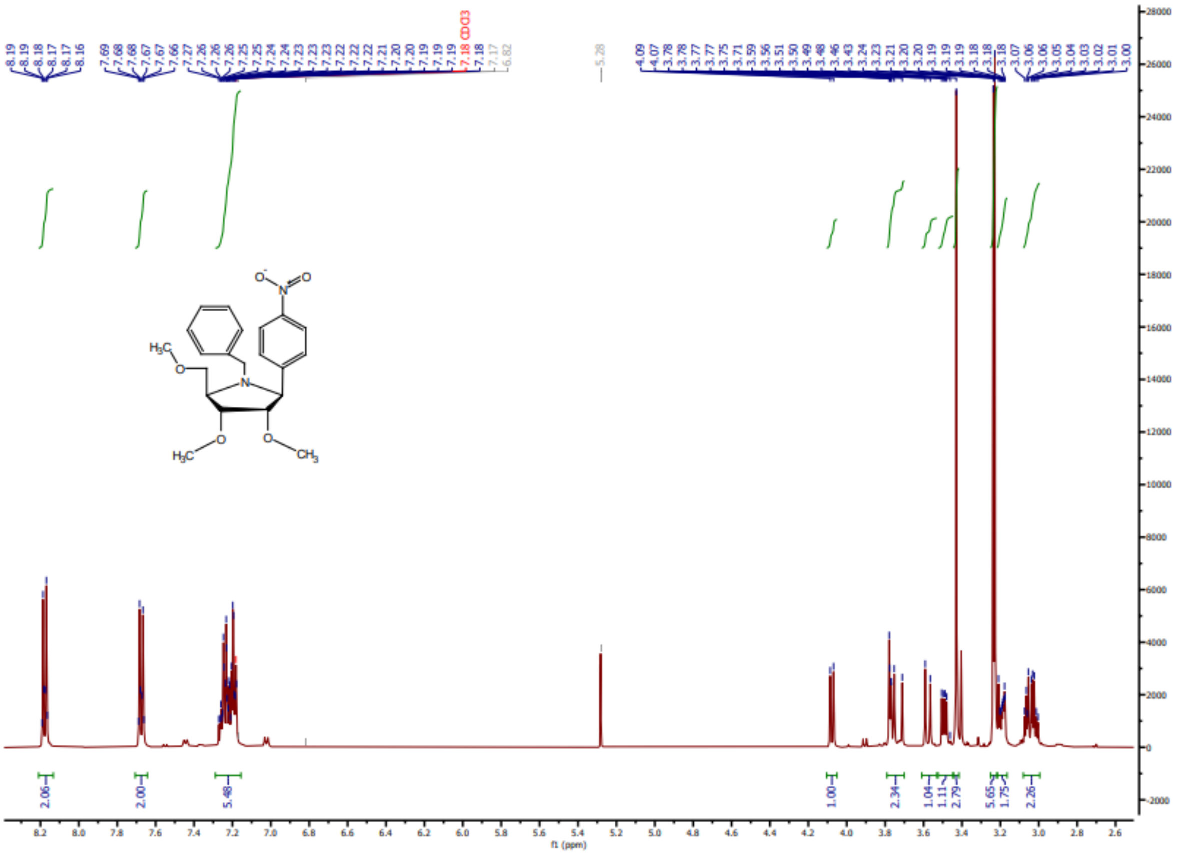

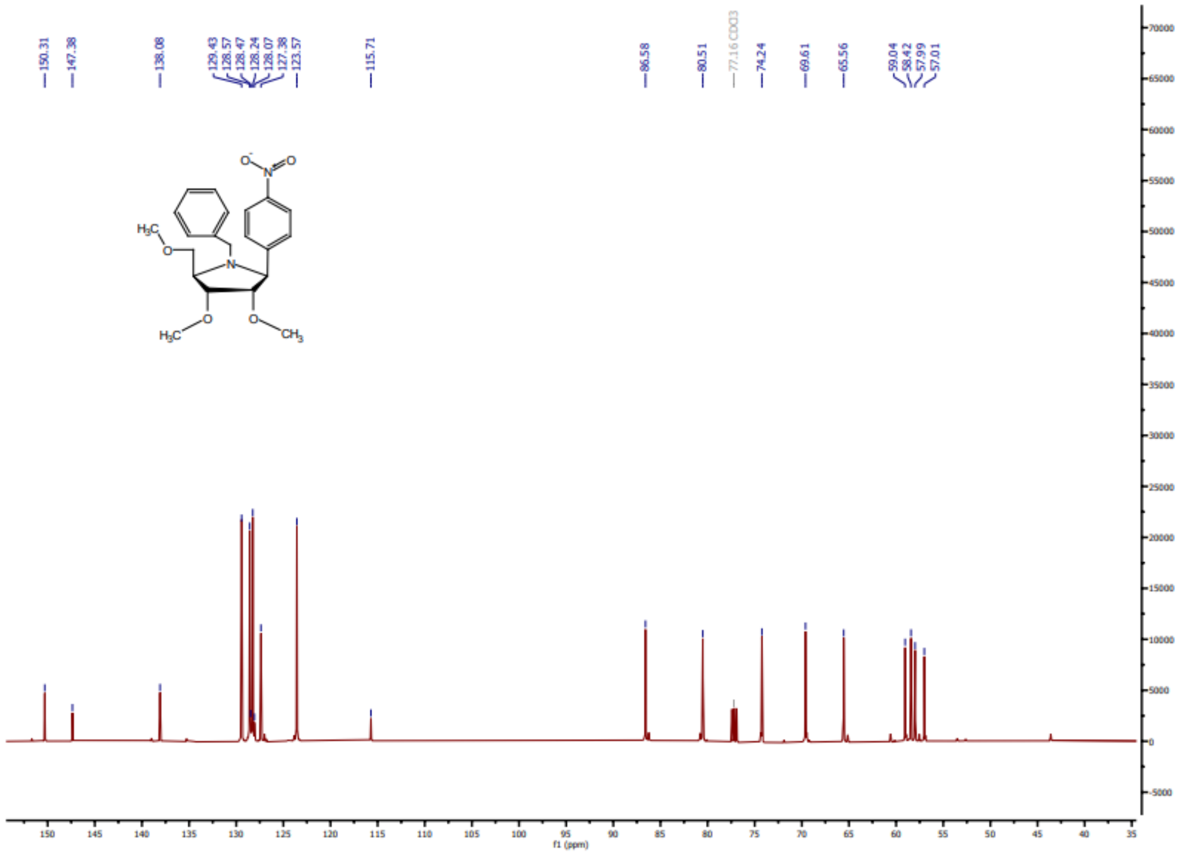

Compound **11**

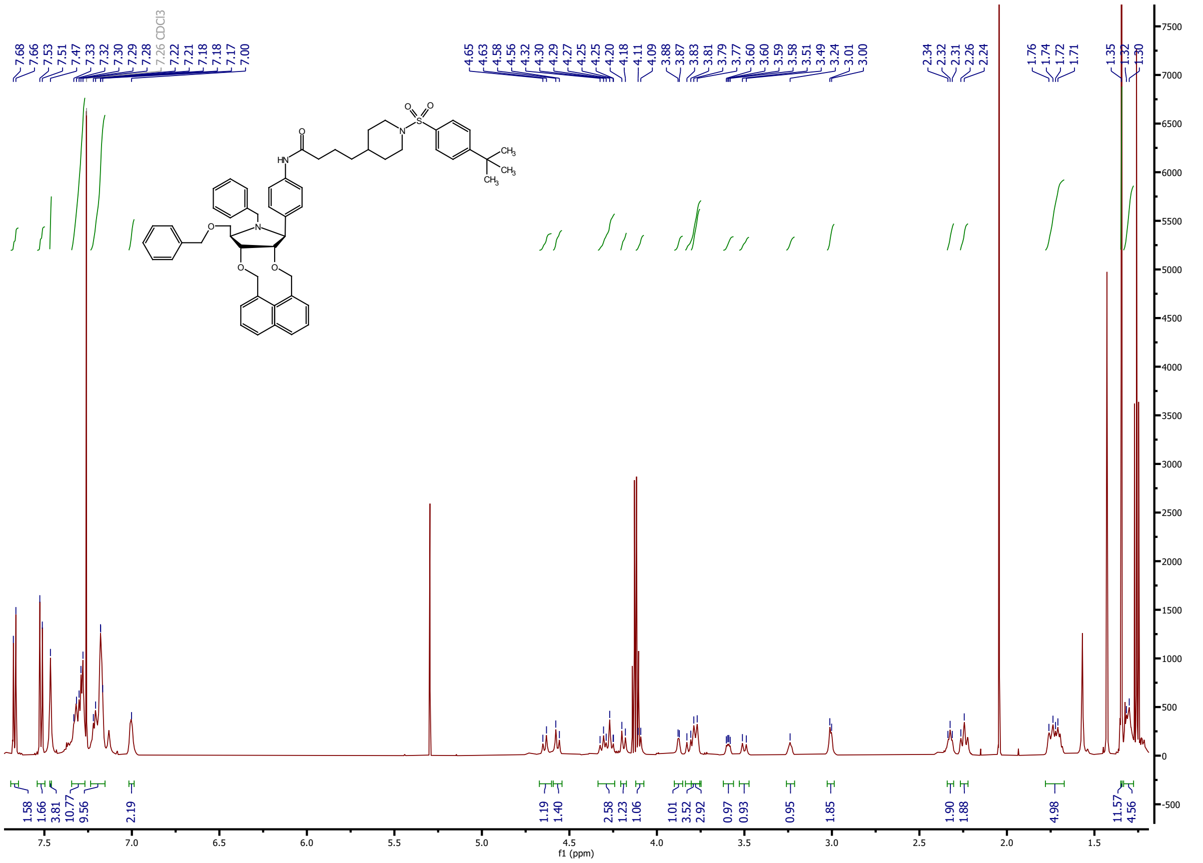

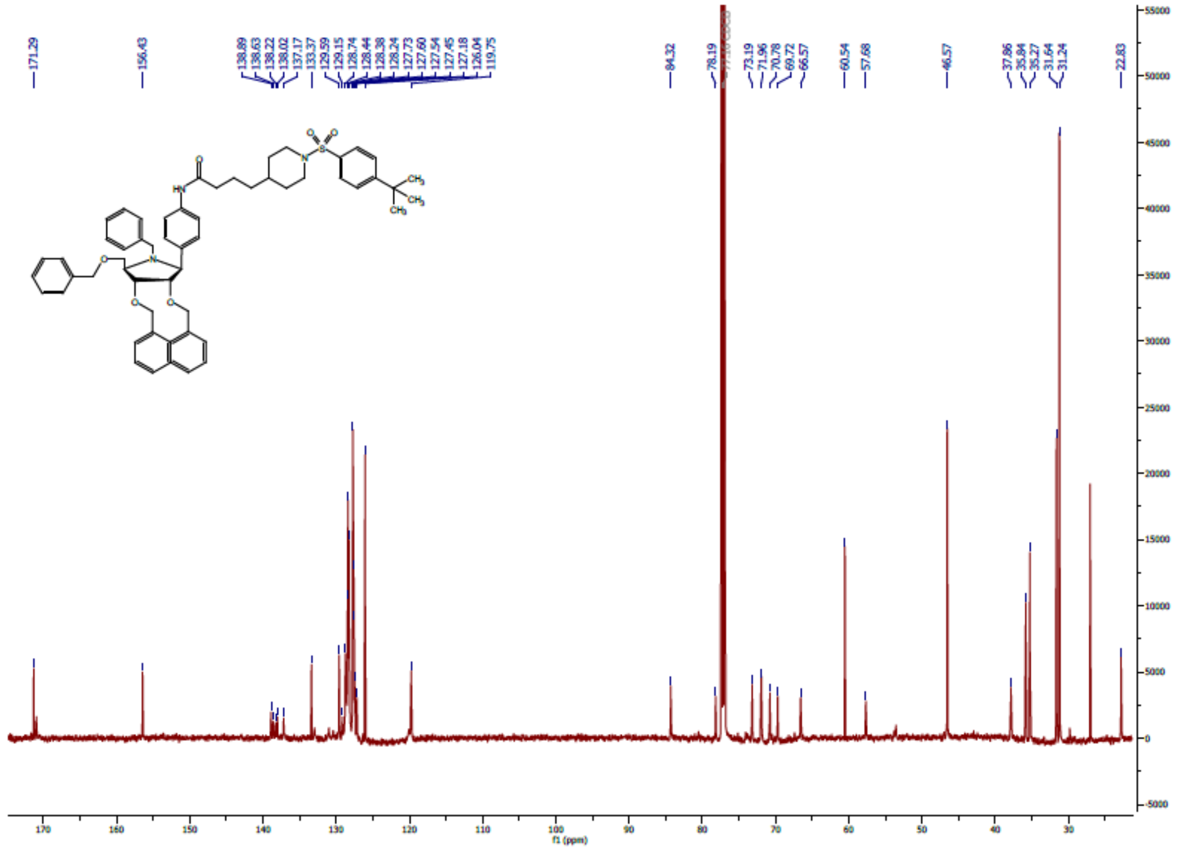

Compound **12**

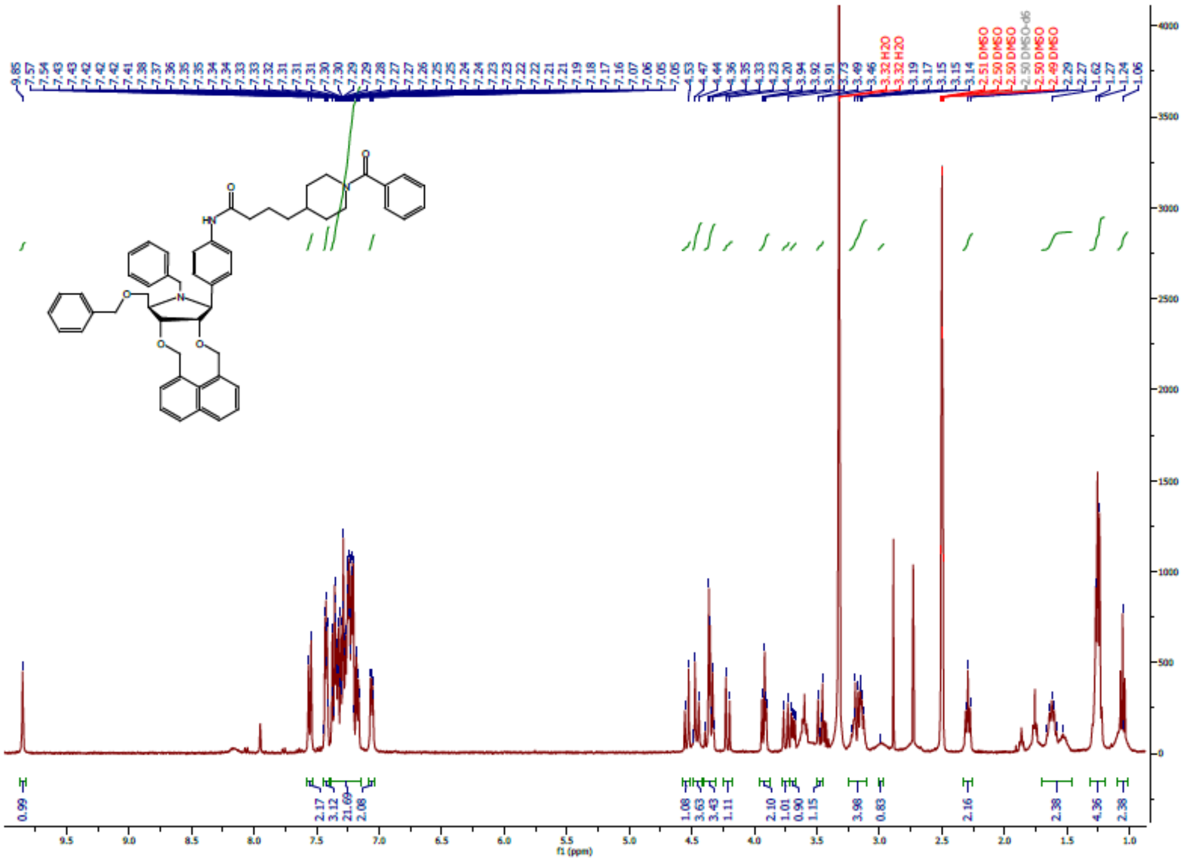

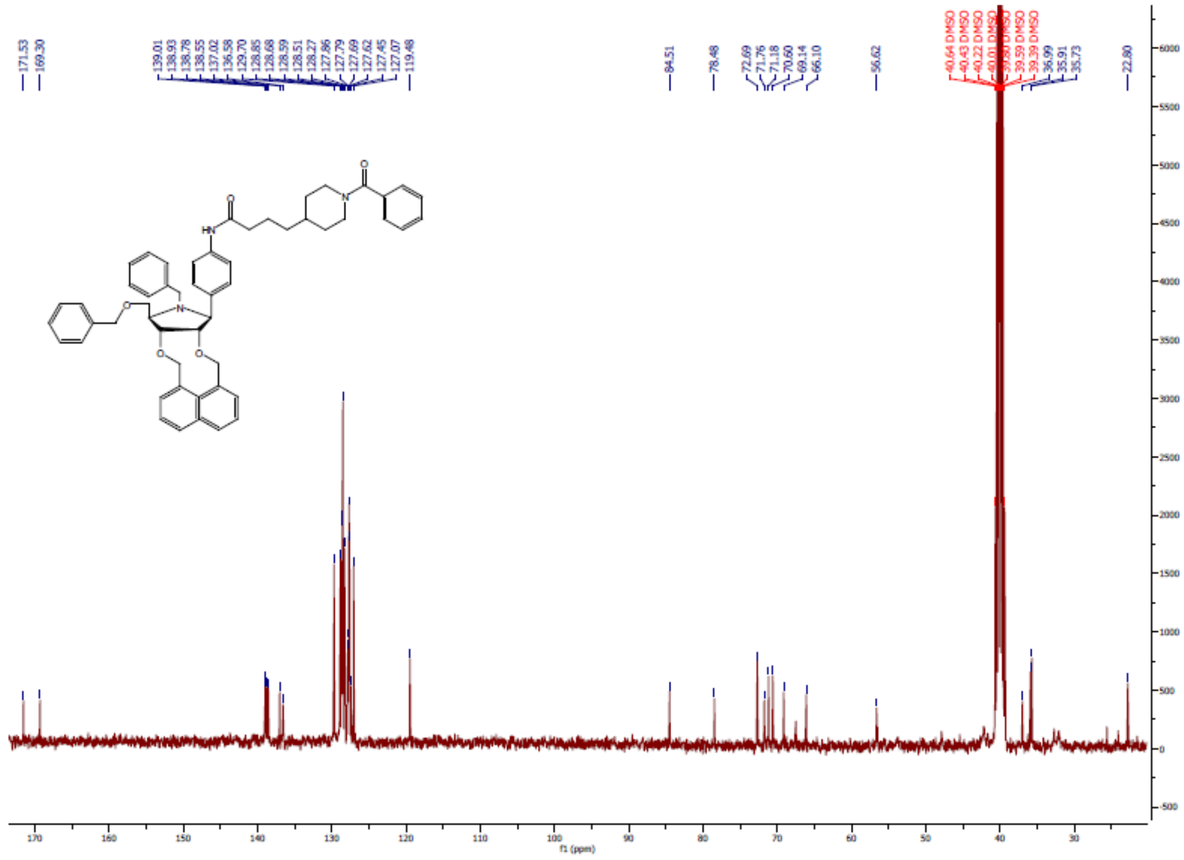

Compound **13**

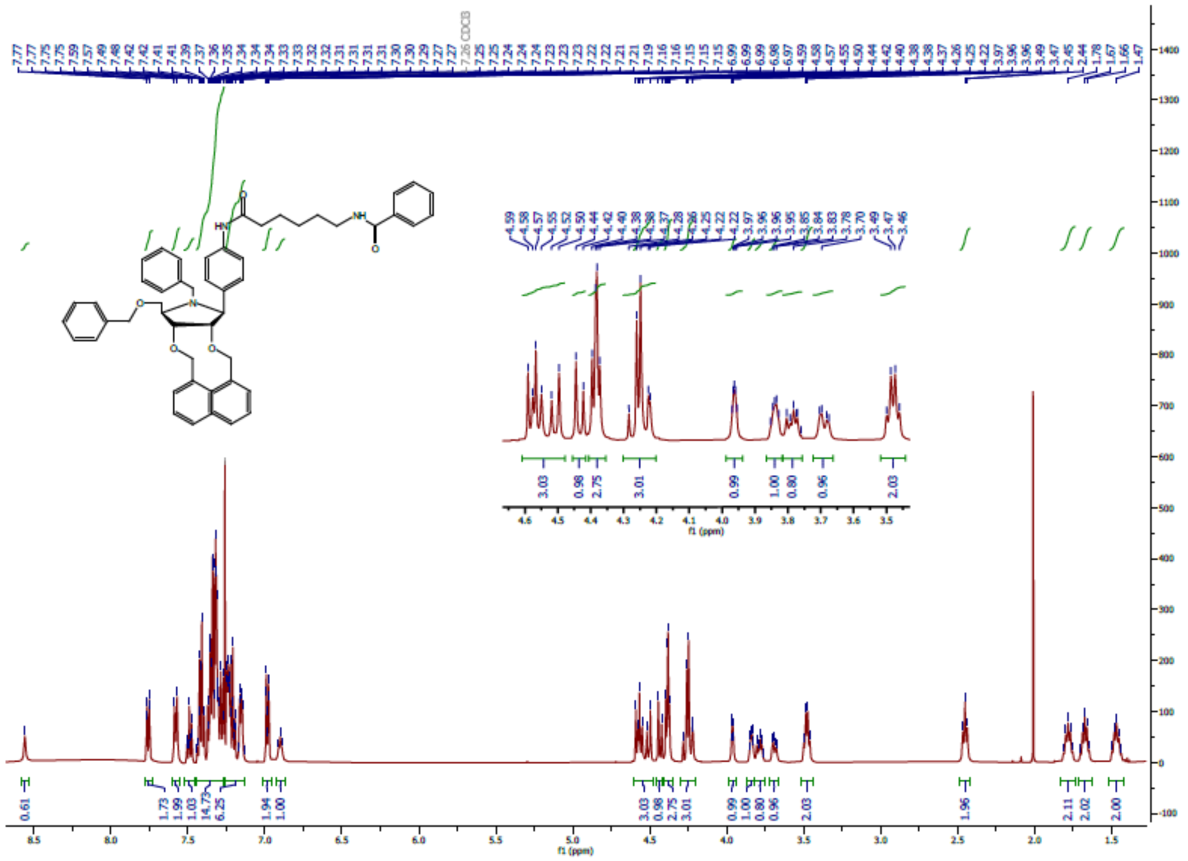

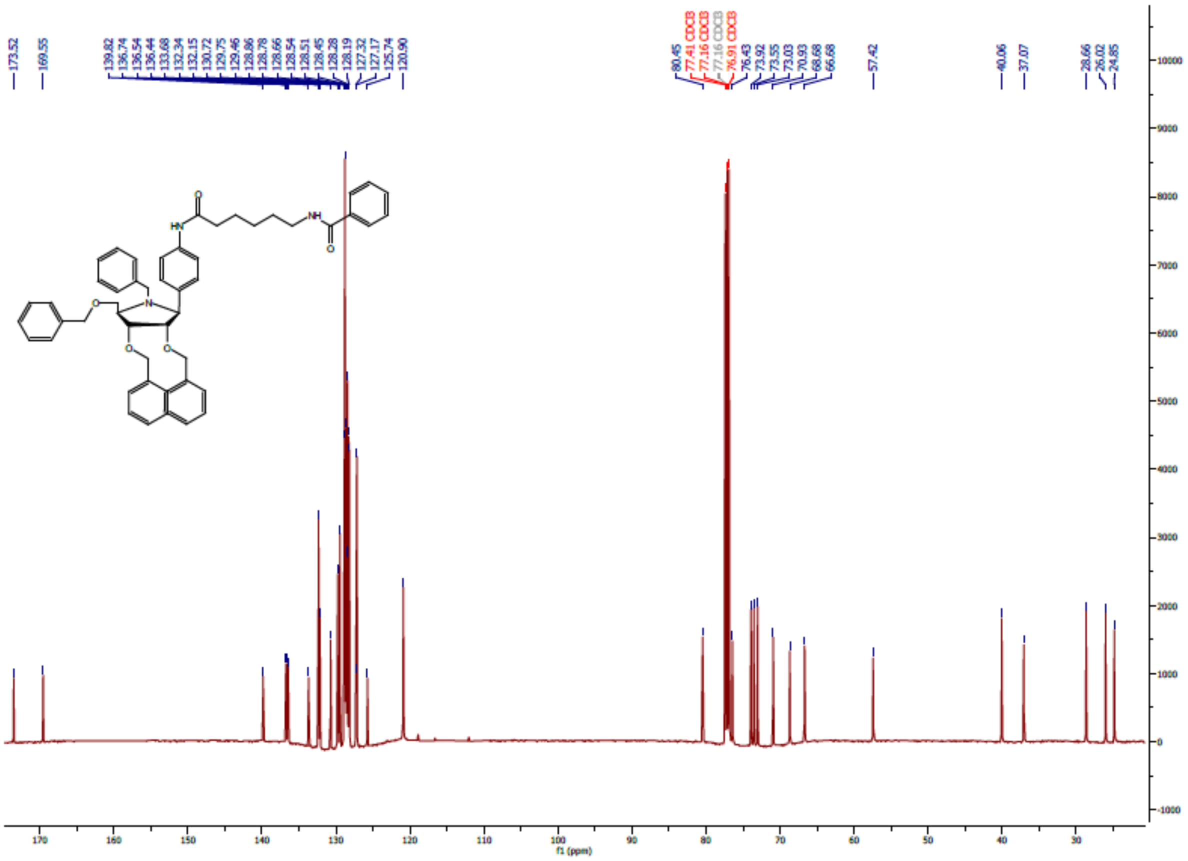

Compound **14**

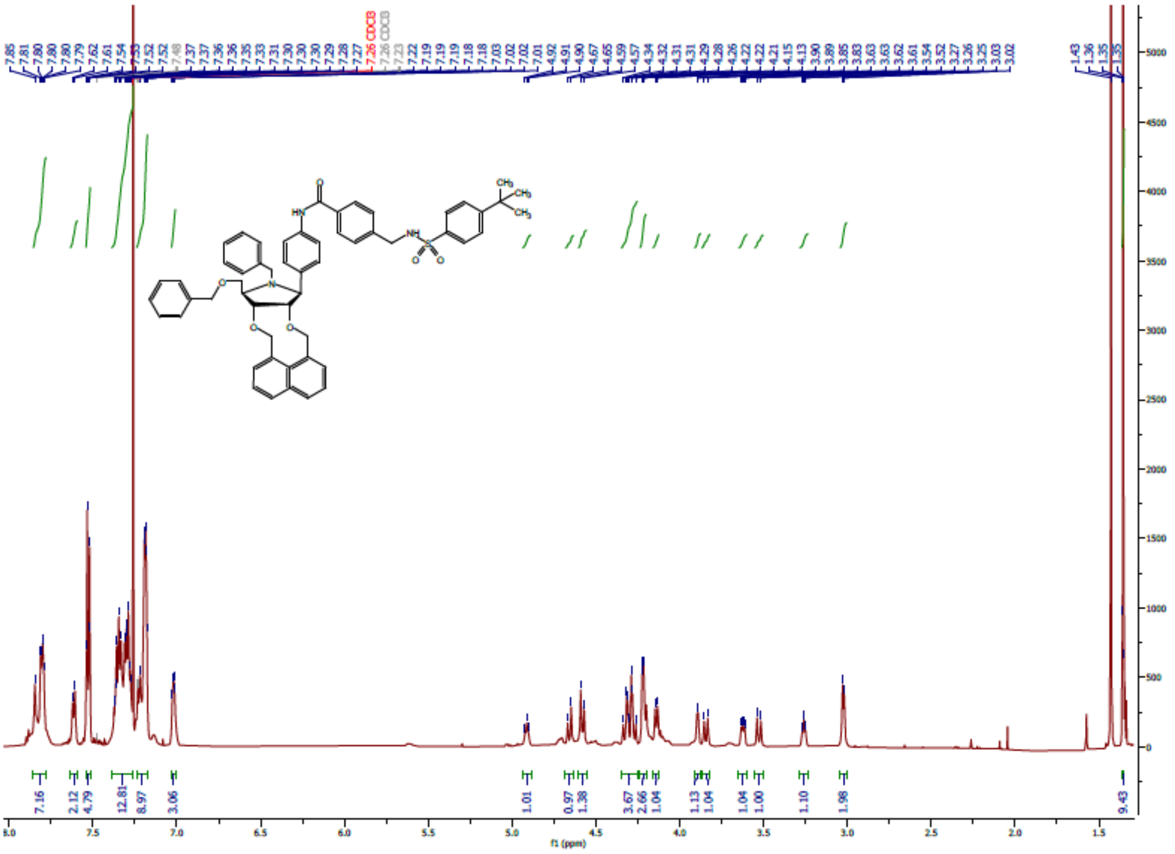
7.85 7.81 7.80 7.80 7.80 7.79 7.62 7.61 7.54 7.53 7.52 7.52 7.48 7.37 7.37 7.36 7.36 7.35 7.33 7.31 7.30 7.30 7.30 7.30 7.29 7.28 7.27 7.26 CDCl3 7.26 CDCl3 7.23 7.22 7.19 7.19 7.19 7.18 7.18 7.03 7.02 7.01 4.92 4.91 4.90 4.67 4.65 4.59 4.57 4.34 4.32 4.31 4.31 4.29 4.28 4.26 4.22 4.22 4.21 4.15 4.13 3.90 3.89 3.85 3.83 3.63 3.63 3.62 3.61 3.54 3.52 3.27 3.26 3.25 3.03 3.02
1.43 1.36 1.35 1.35
7.16 2.12 4.79 12.81 8.97 3.06 1.01 0.97 1.38 3.67 2.66 1.04 1.13 1.04 1.04 1.00 1.10 1.98 9.43
f1 (ppm)

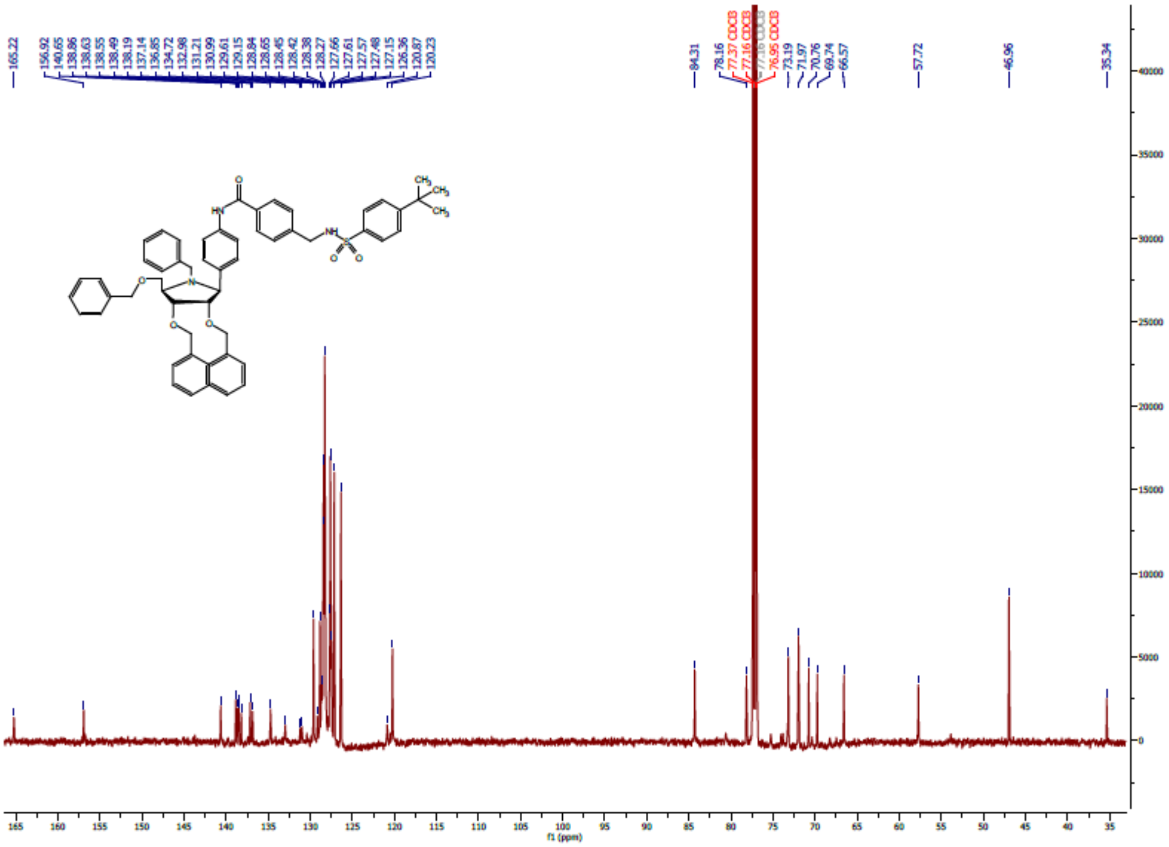
165.22
156.92 140.65 138.86 138.63 138.55 138.49 138.19 137.14 136.85 134.72 132.98 131.21 130.99 129.61 129.15 128.84 128.65 128.45 128.42 128.38 128.27 127.66 127.61 127.57 127.48 127.15 126.36 120.87 120.23
84.31 78.16 77.37 CDCl3 77.16 CDCl3 77.16 CDCl3 76.95 CDCl3 73.19 71.97 70.76 69.74 66.57
57.72
46.96
35.34
f1 (ppm)

Compound **15**

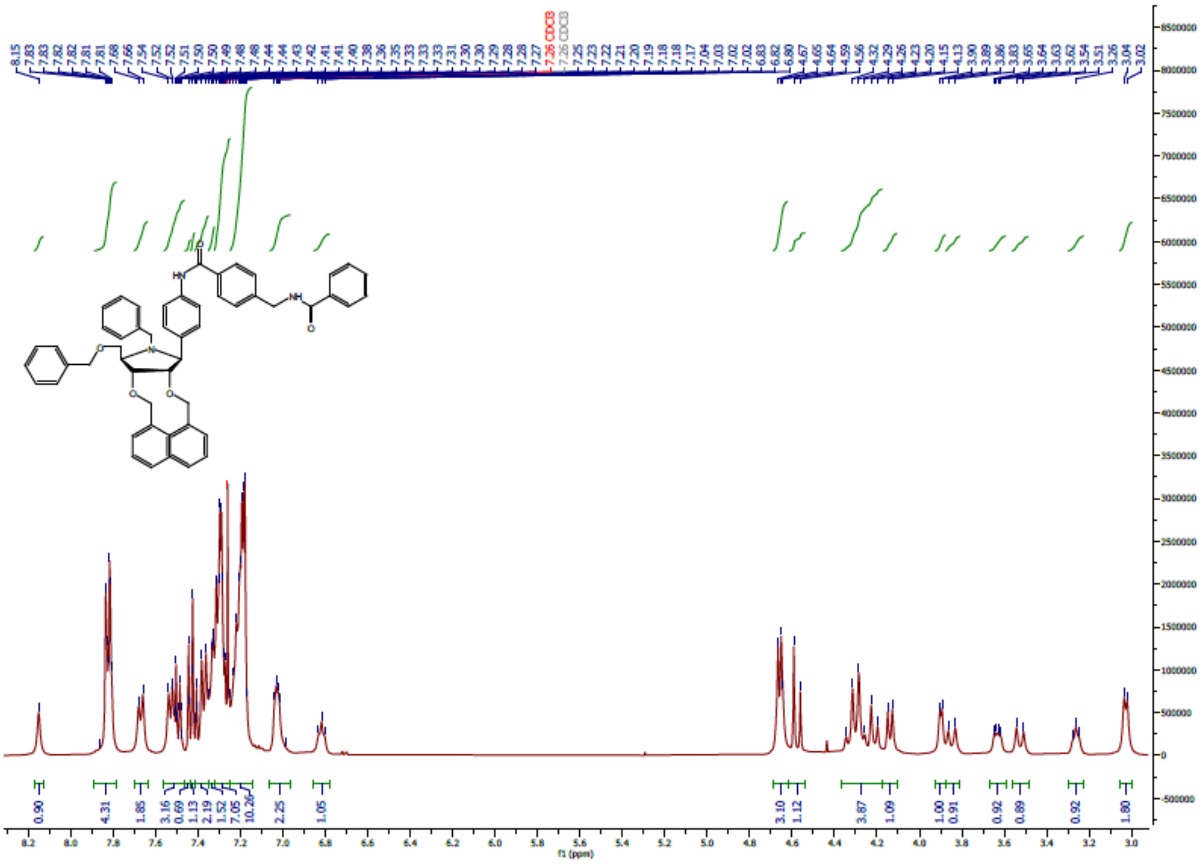

Compound **16**

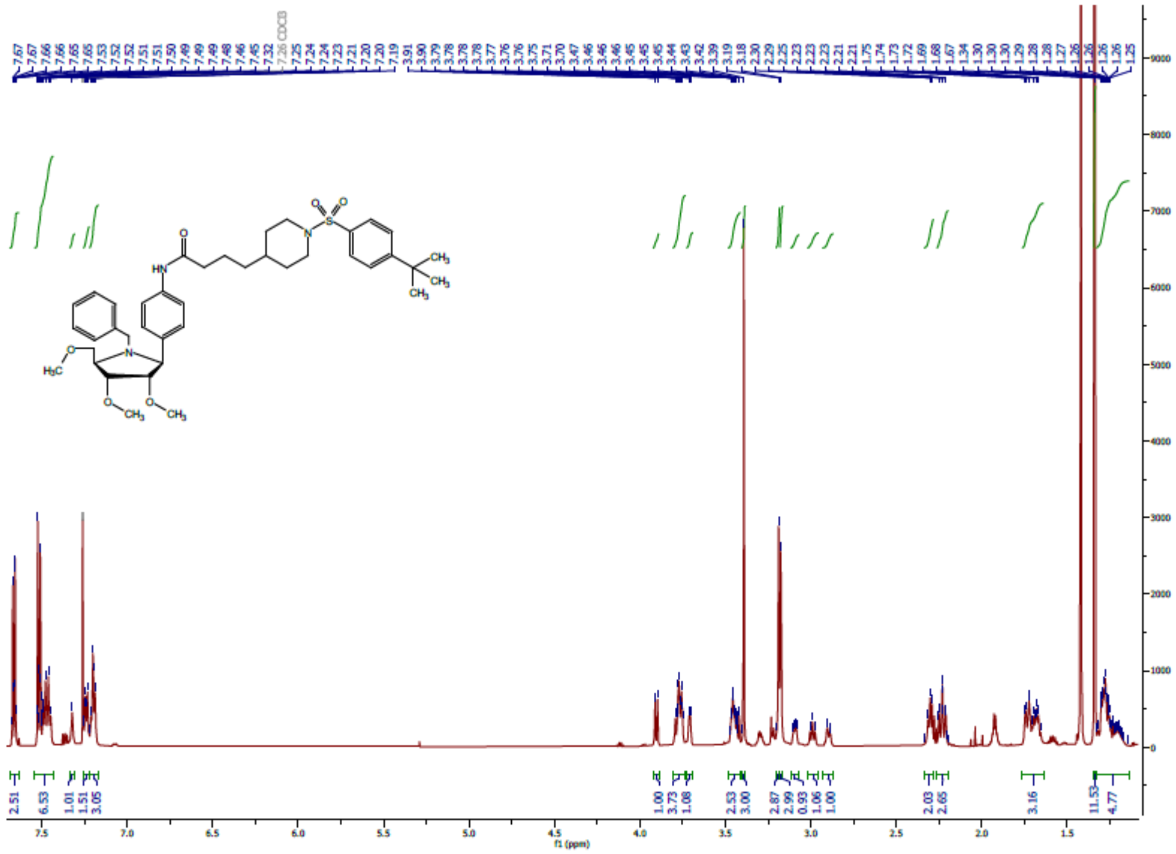

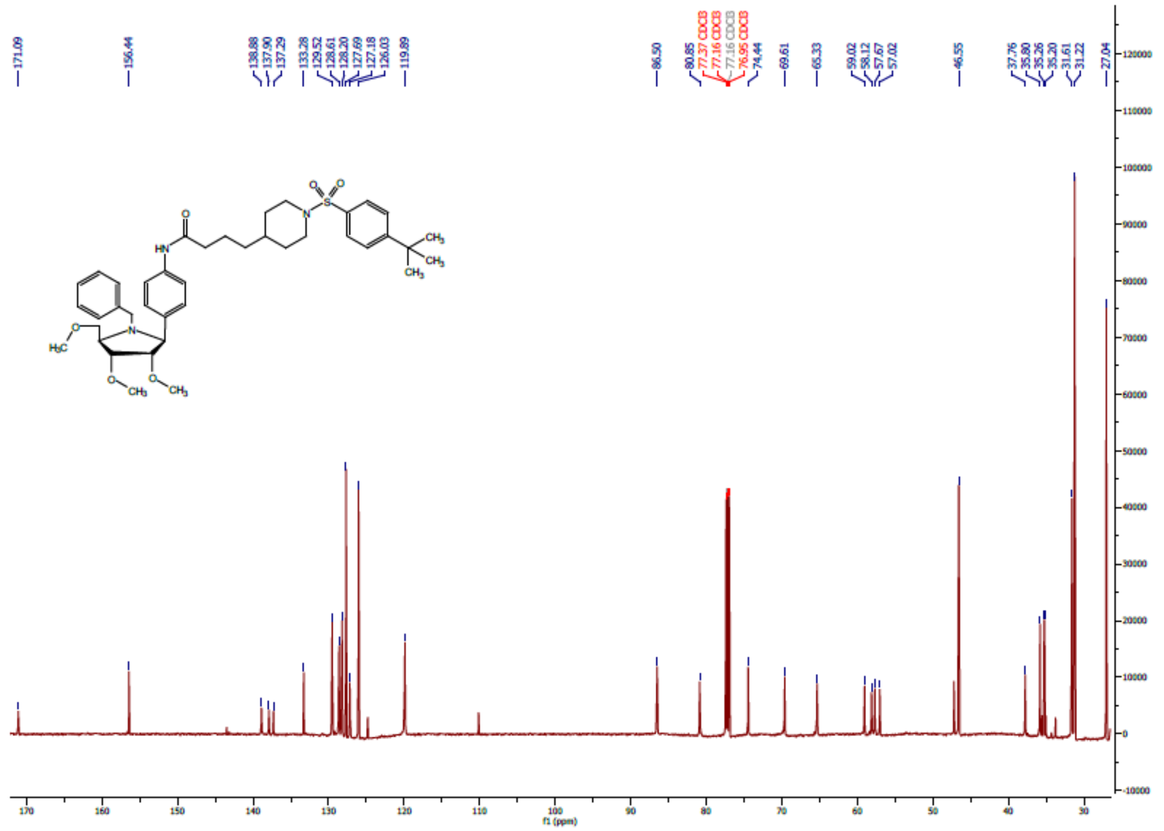

Compound **17**

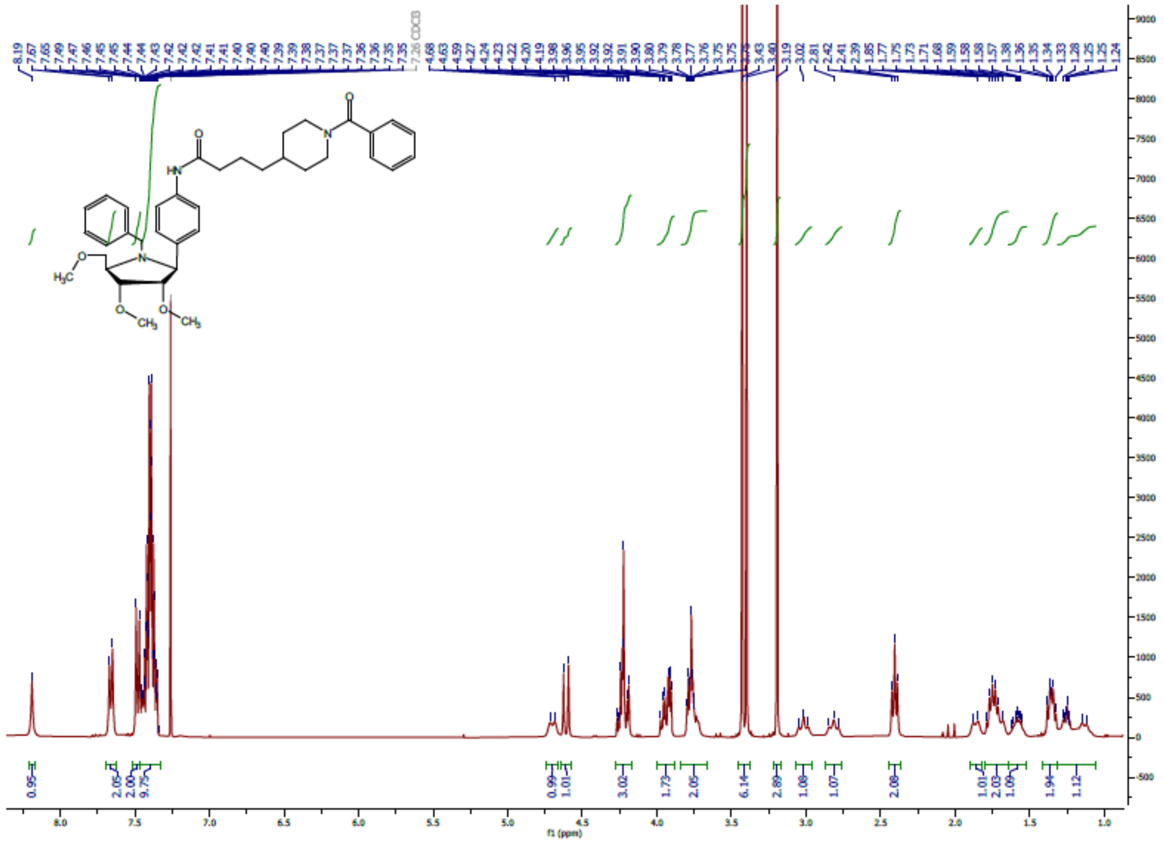

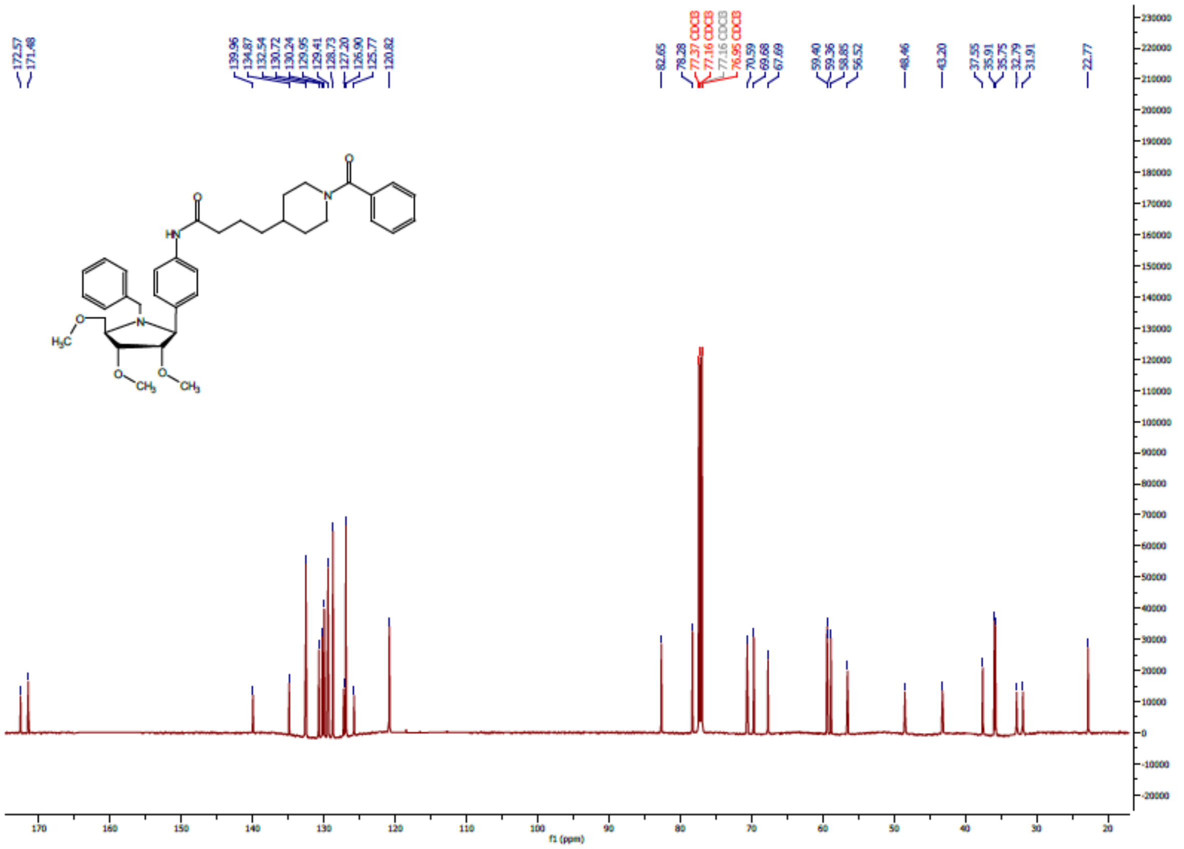

Compound **18**

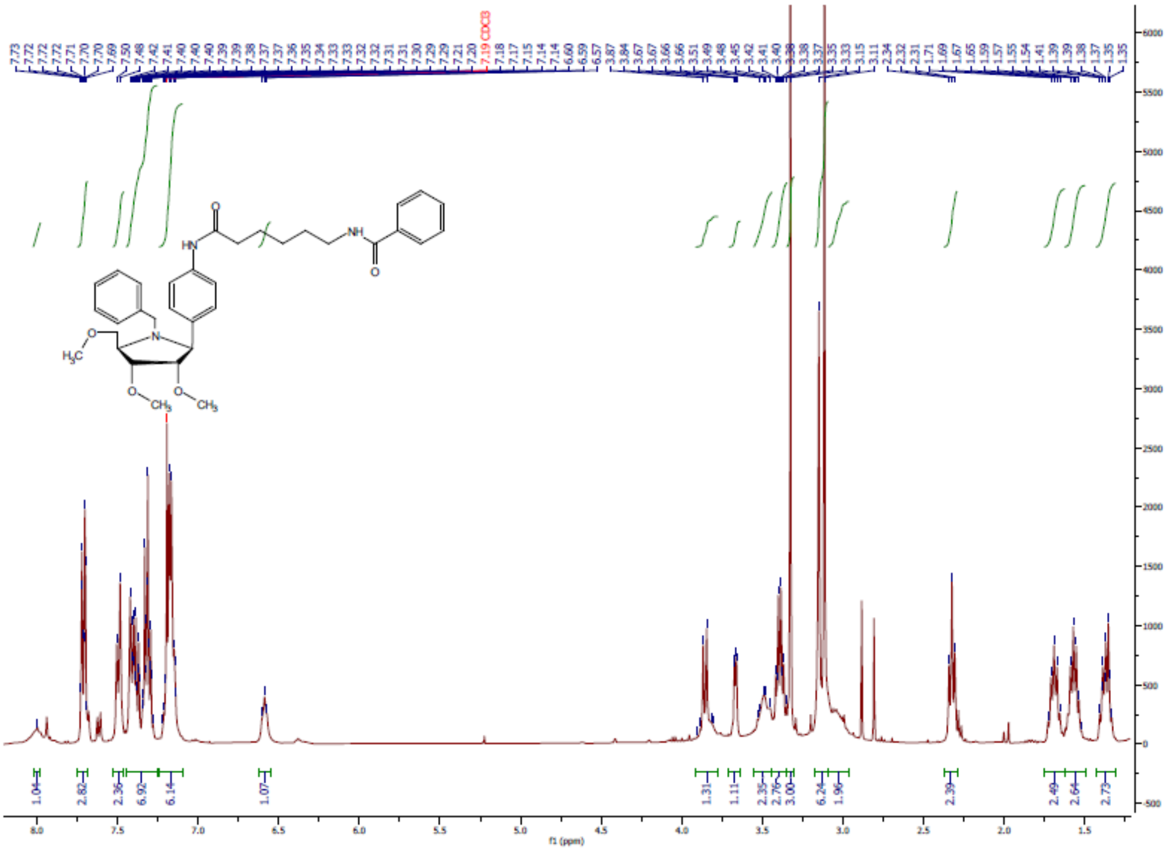
7.73 7.72 7.72 7.72 7.71 7.70 7.70 7.69 7.50 7.48 7.42 7.41 7.40 7.40 7.40 7.39 7.39 7.38 7.37 7.37 7.36 7.35 7.34 7.33 7.33 7.32 7.32 7.31 7.31 7.30 7.29 7.29 7.21 7.20 7.19 CDCl3 7.18 7.17 7.15 7.14 7.14 6.60 6.59 6.57 3.87 3.84 3.67 3.66 3.66 3.51 3.49 3.48 3.45 3.42 3.41 3.40 3.38 3.38 3.37 3.35 3.33 3.15 3.11 2.34 2.32 2.31 1.71 1.69 1.67 1.65 1.59 1.57 1.55 1.54 1.41 1.39 1.39 1.38 1.37 1.35 1.35
1.04 2.82 2.36 6.92 6.14 1.07 1.31 1.11 2.35 2.76 3.00 6.24 1.96 2.39 2.49 2.64 2.73
HN
NH
O
N
O
H3C
O
CH3
O
CH3
f1 (ppm)

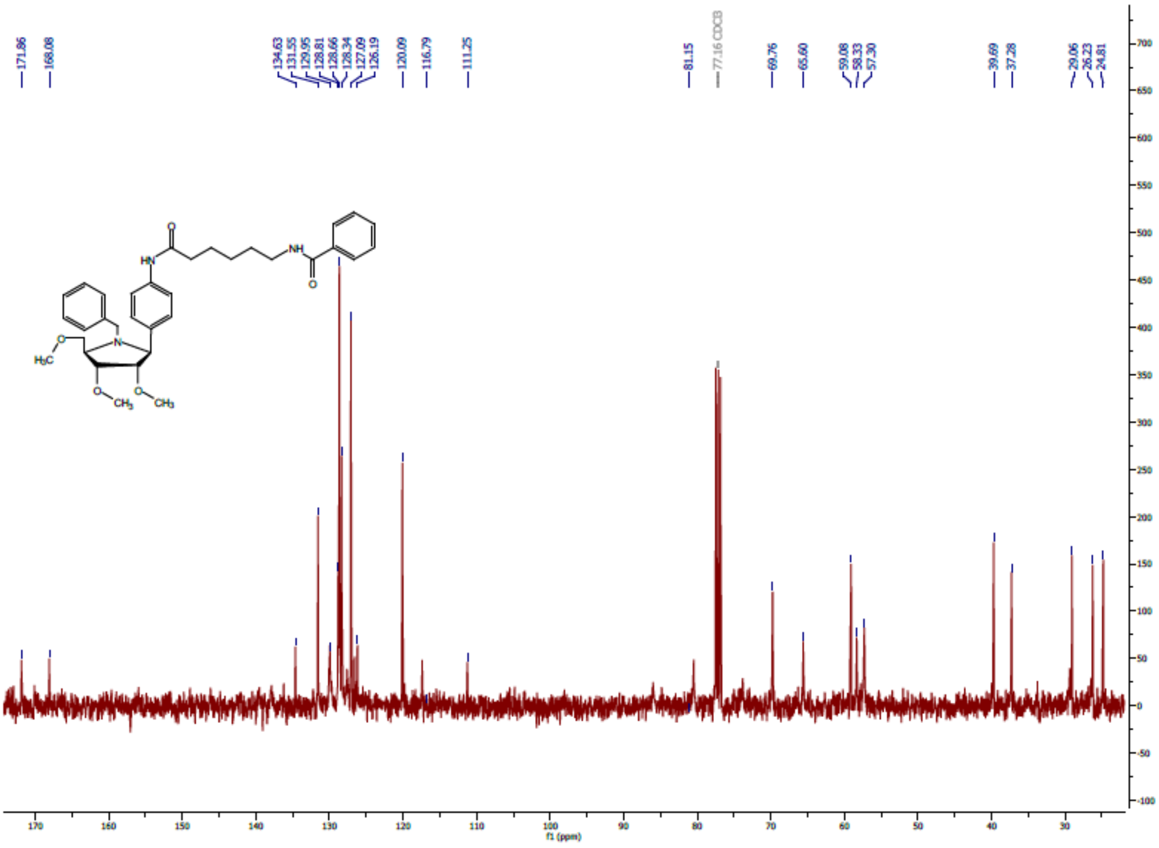
171.86 168.08 134.63 131.55 129.95 128.81 128.66 128.34 127.09 126.19 120.09 116.79 111.25 81.15 77.16 CDCl3 69.76 65.60 59.08 58.33 57.30 39.69 37.28 29.06 26.23 24.81
HN
NH
O
N
O
H3C
O
CH3
O
CH3
f1 (ppm)

Compound **19**

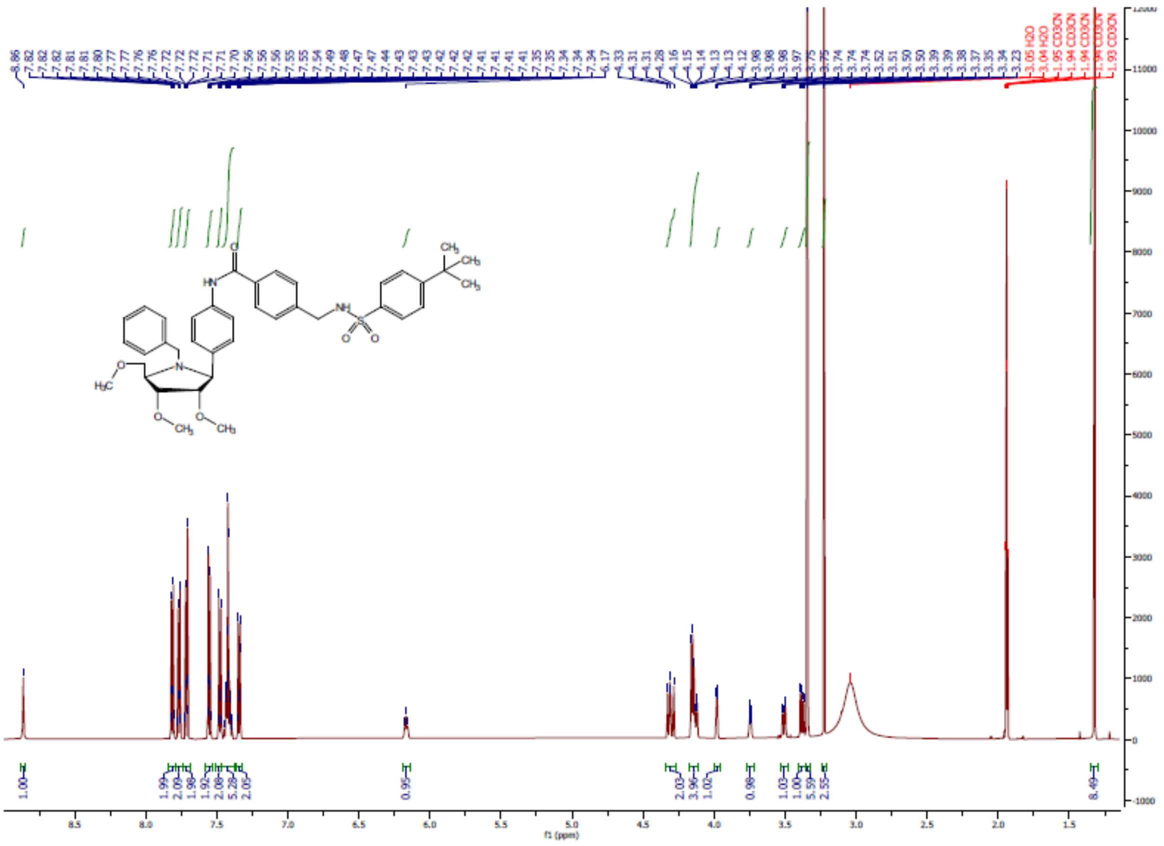

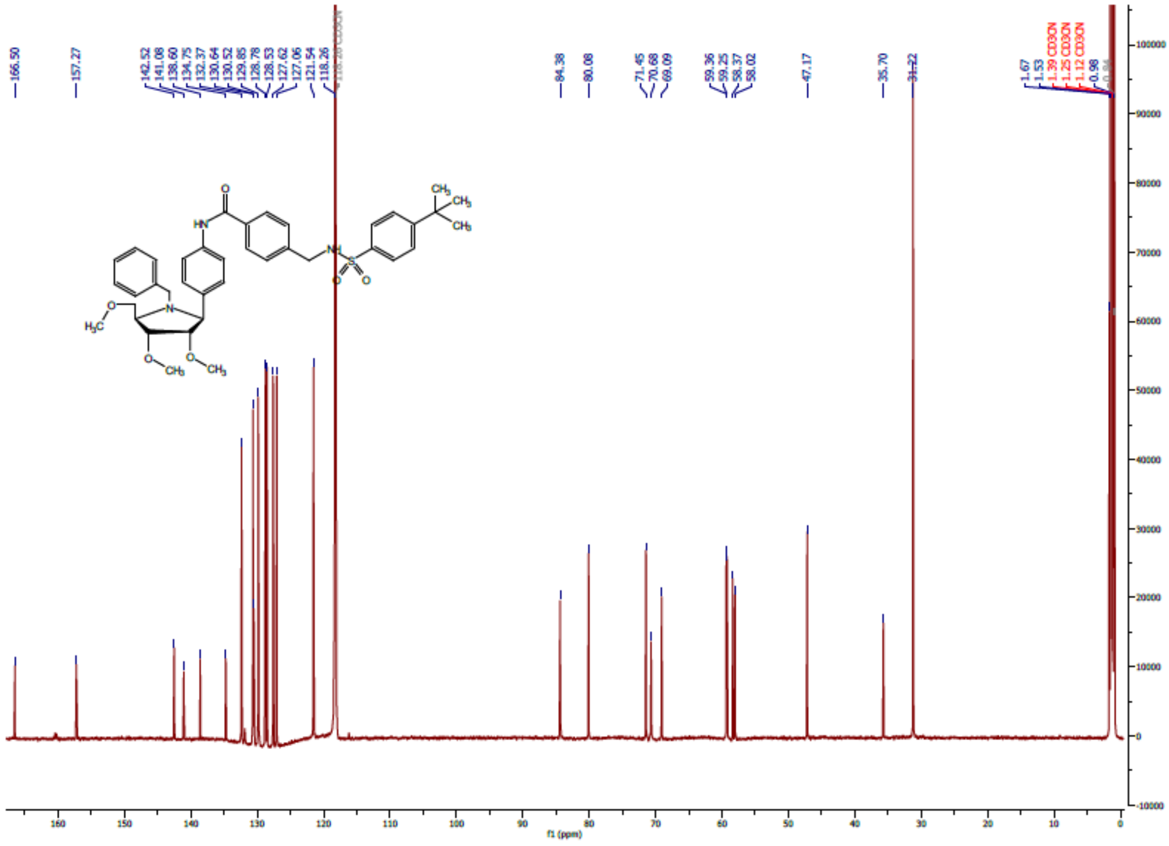

Compound **20**

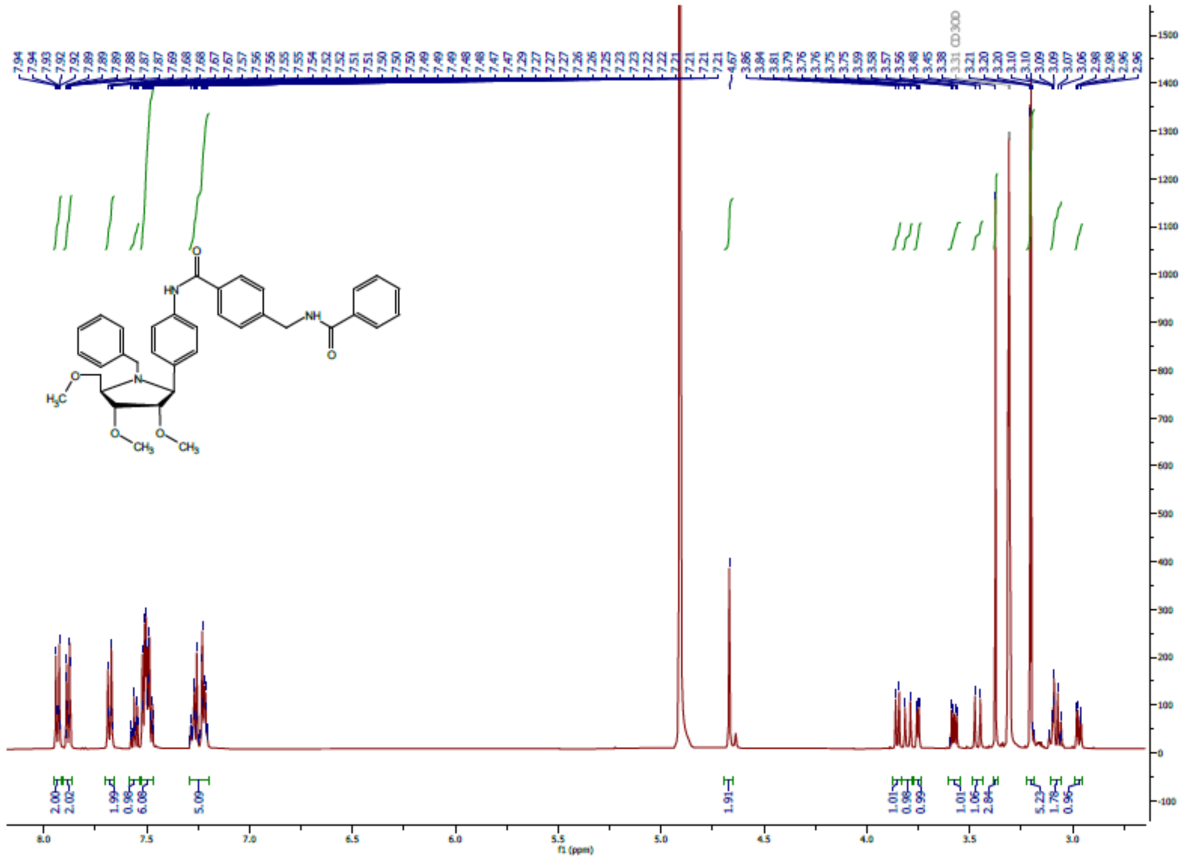

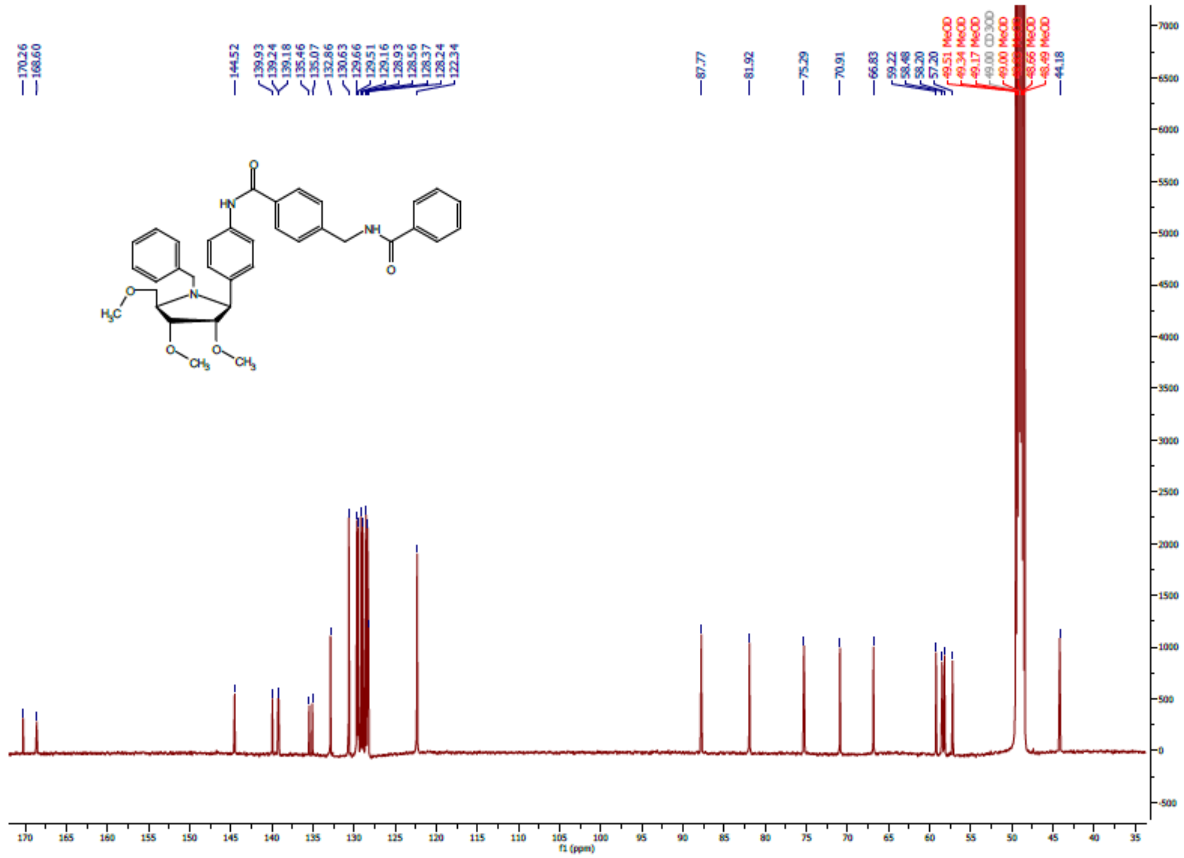

Compound **21**

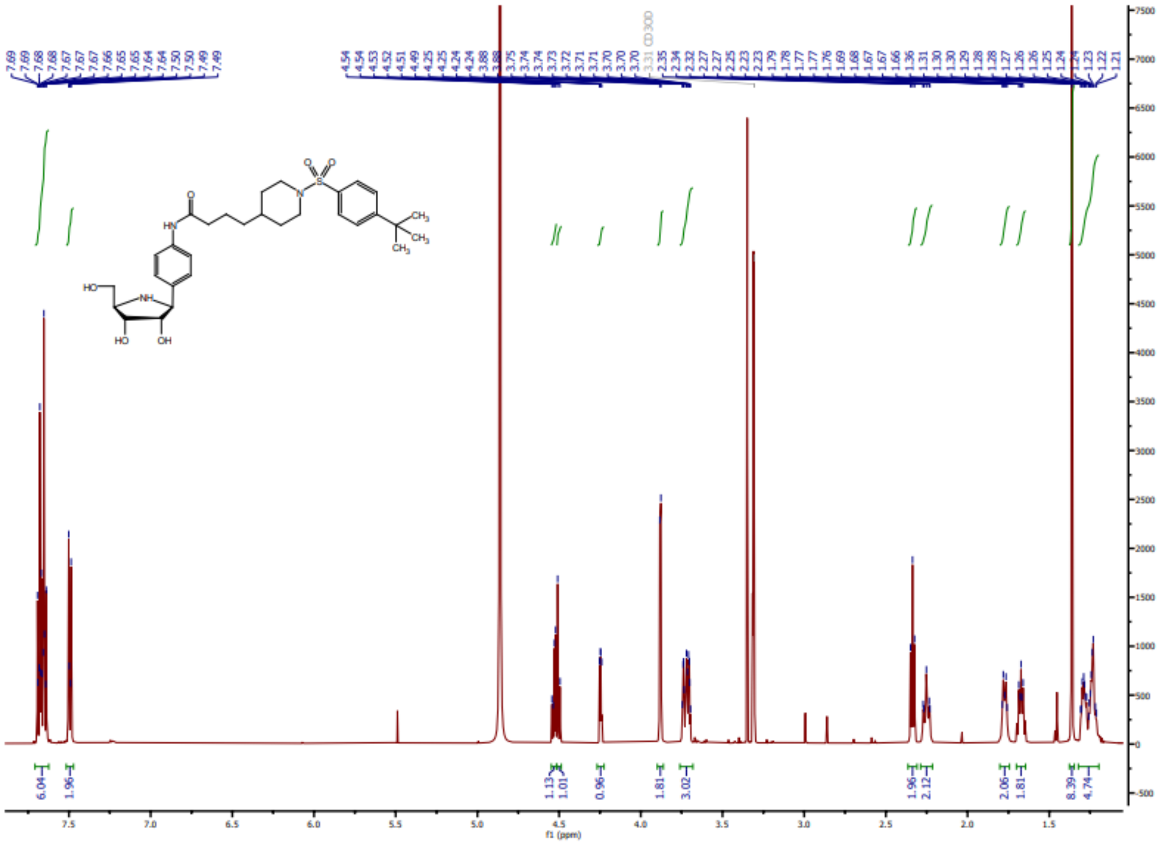

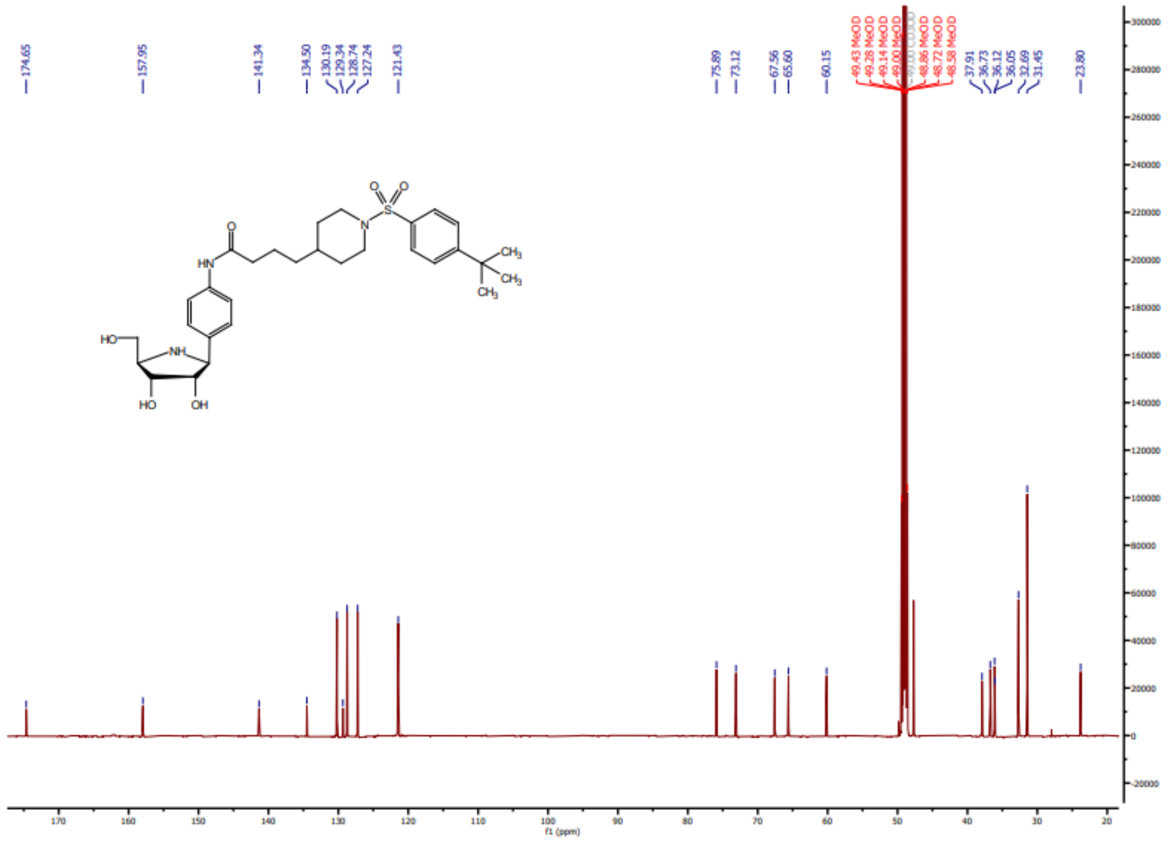

Compound **22**

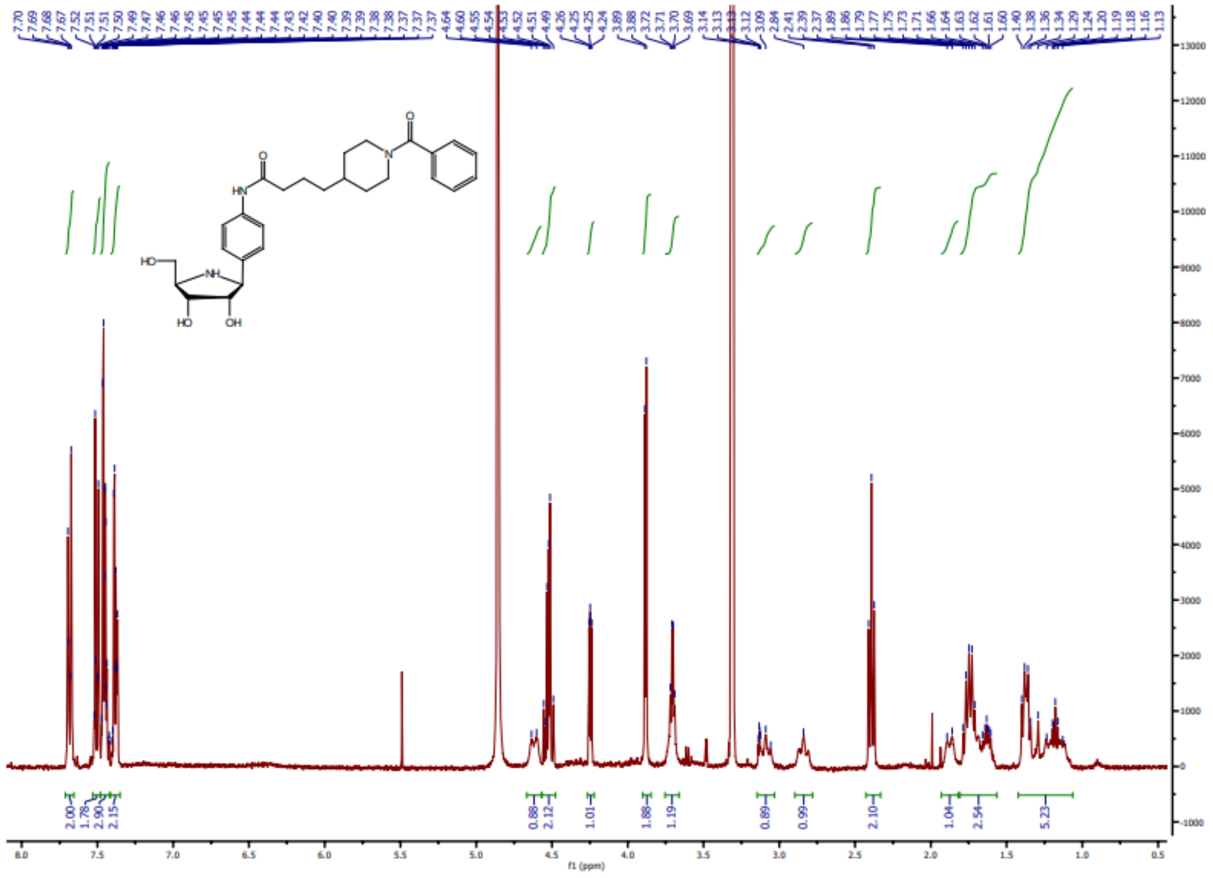

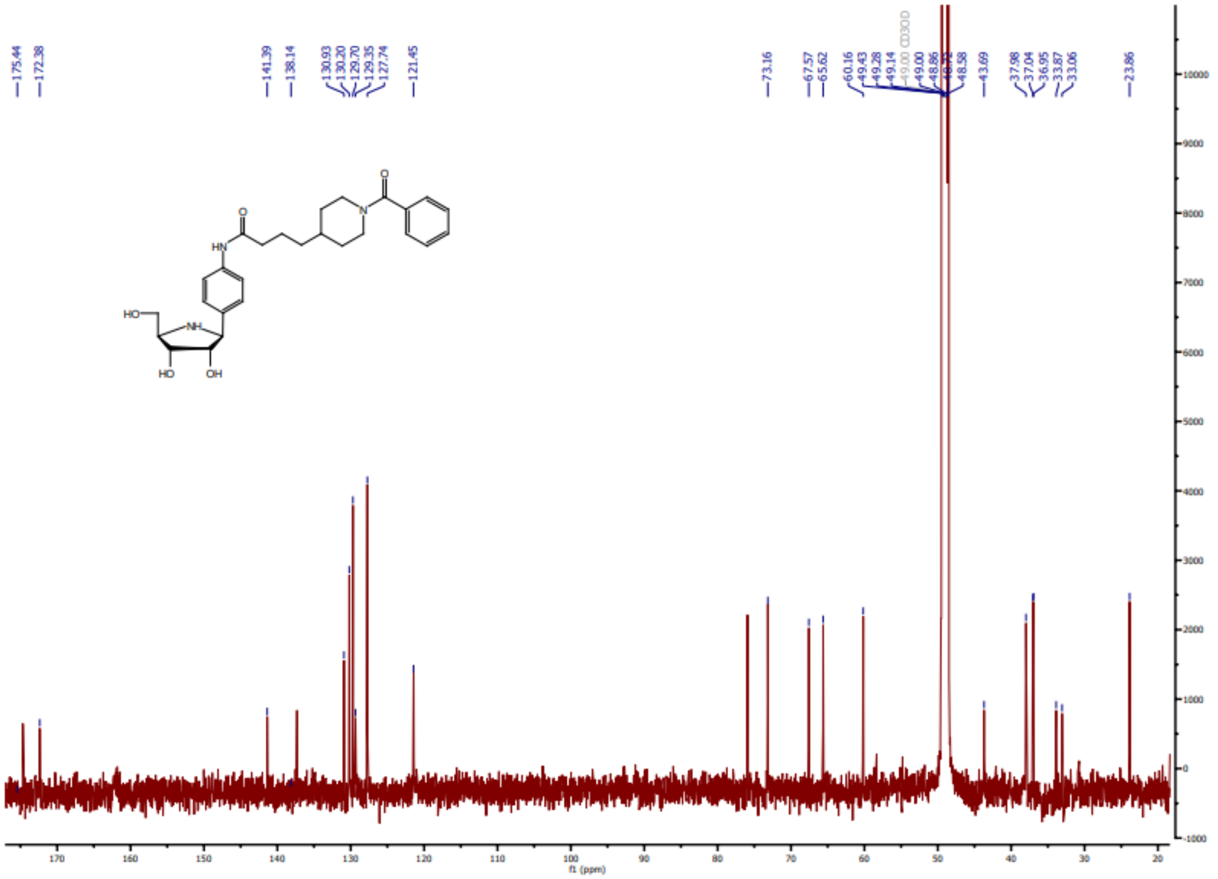

Compound **23**

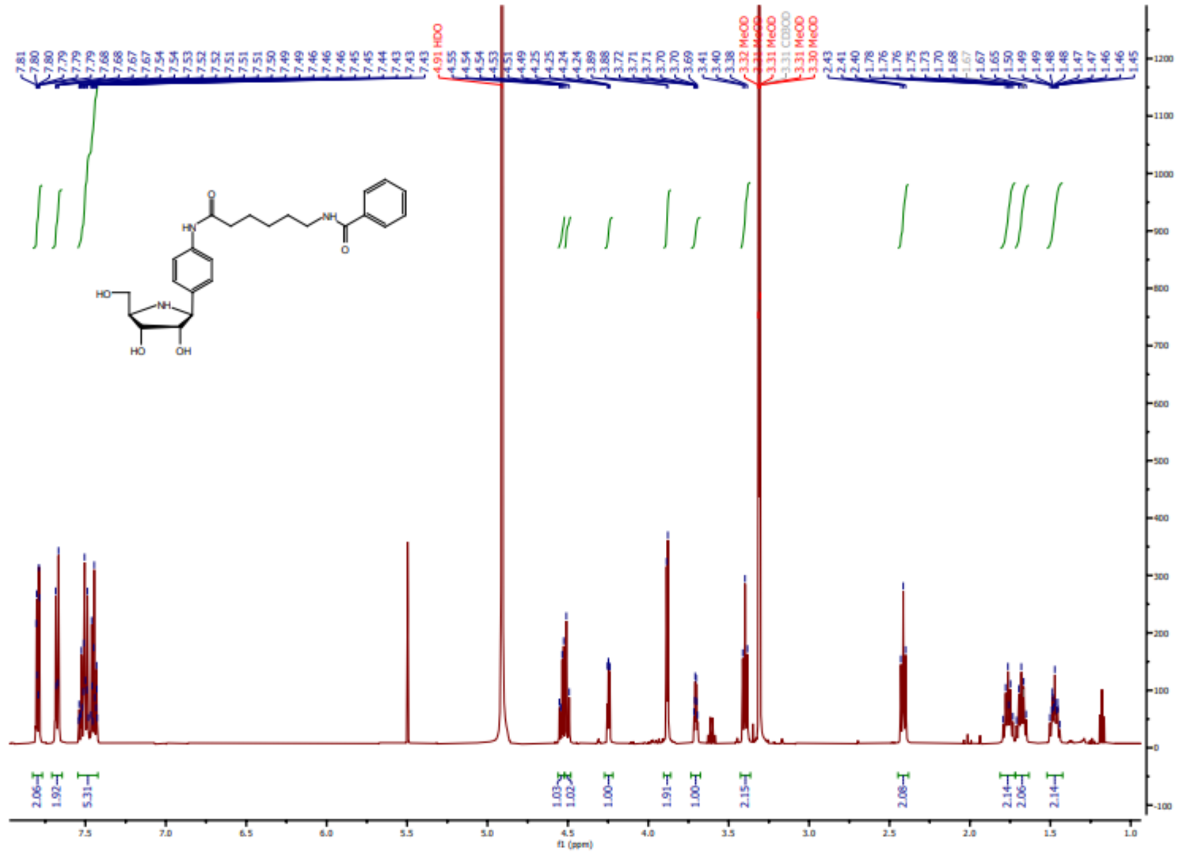

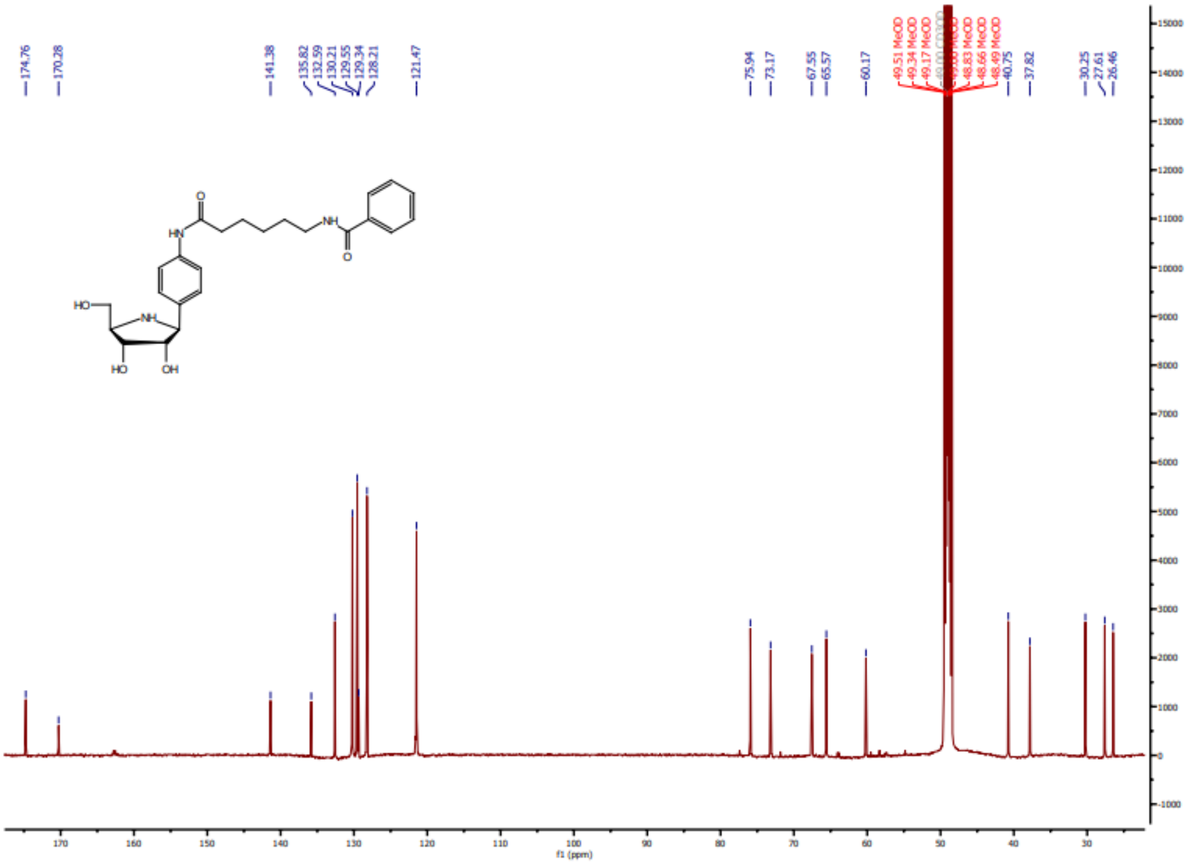

Compound **24**

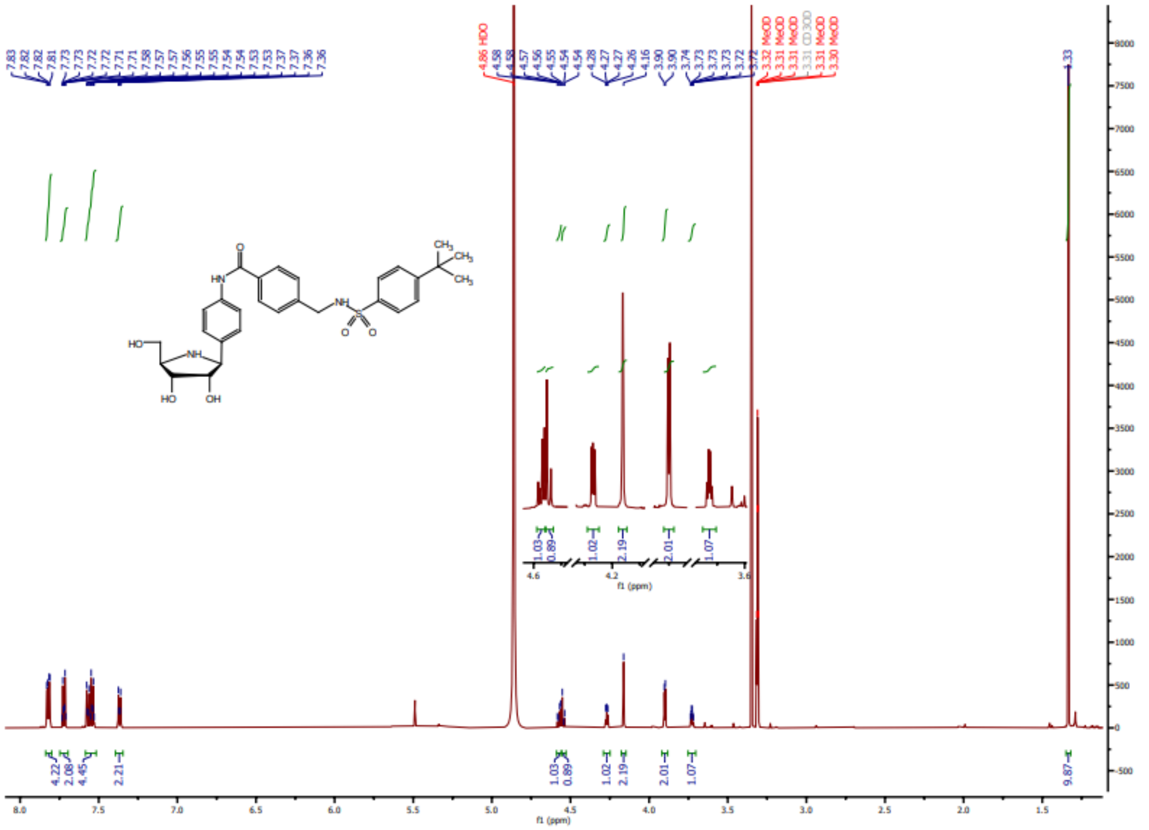

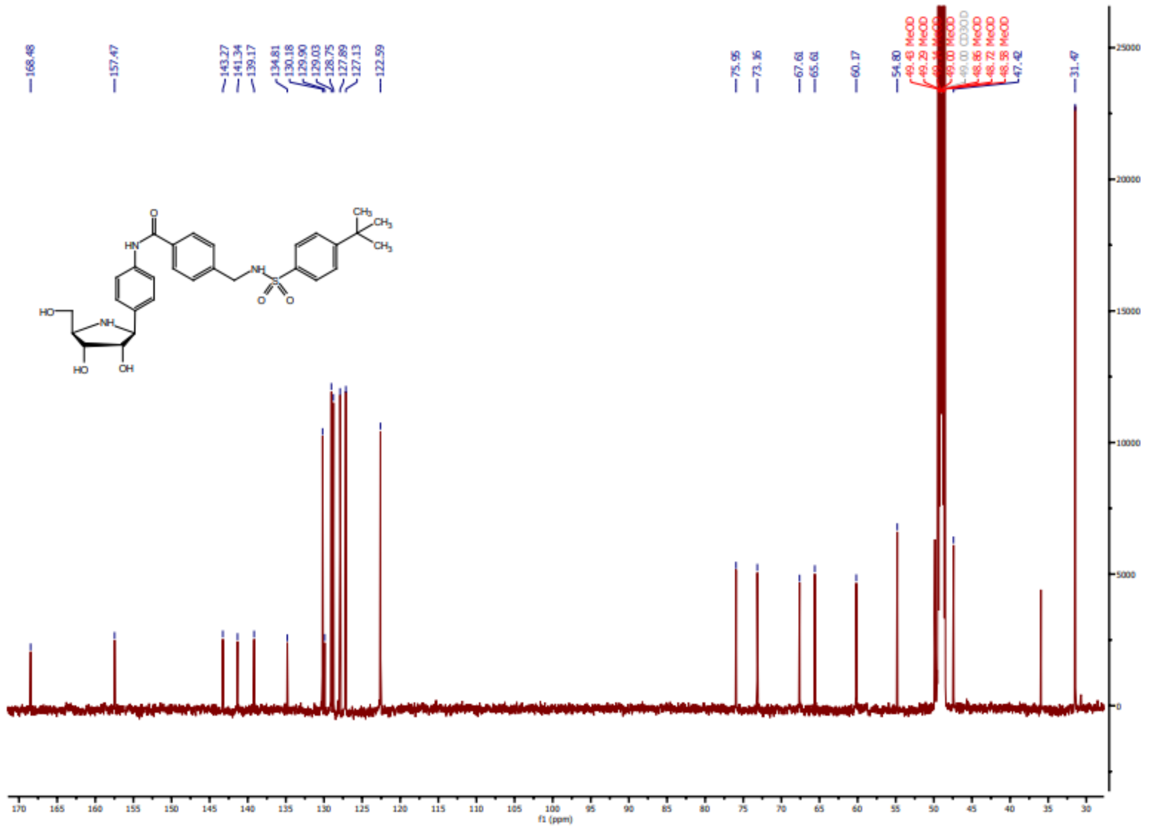

Compound **25**

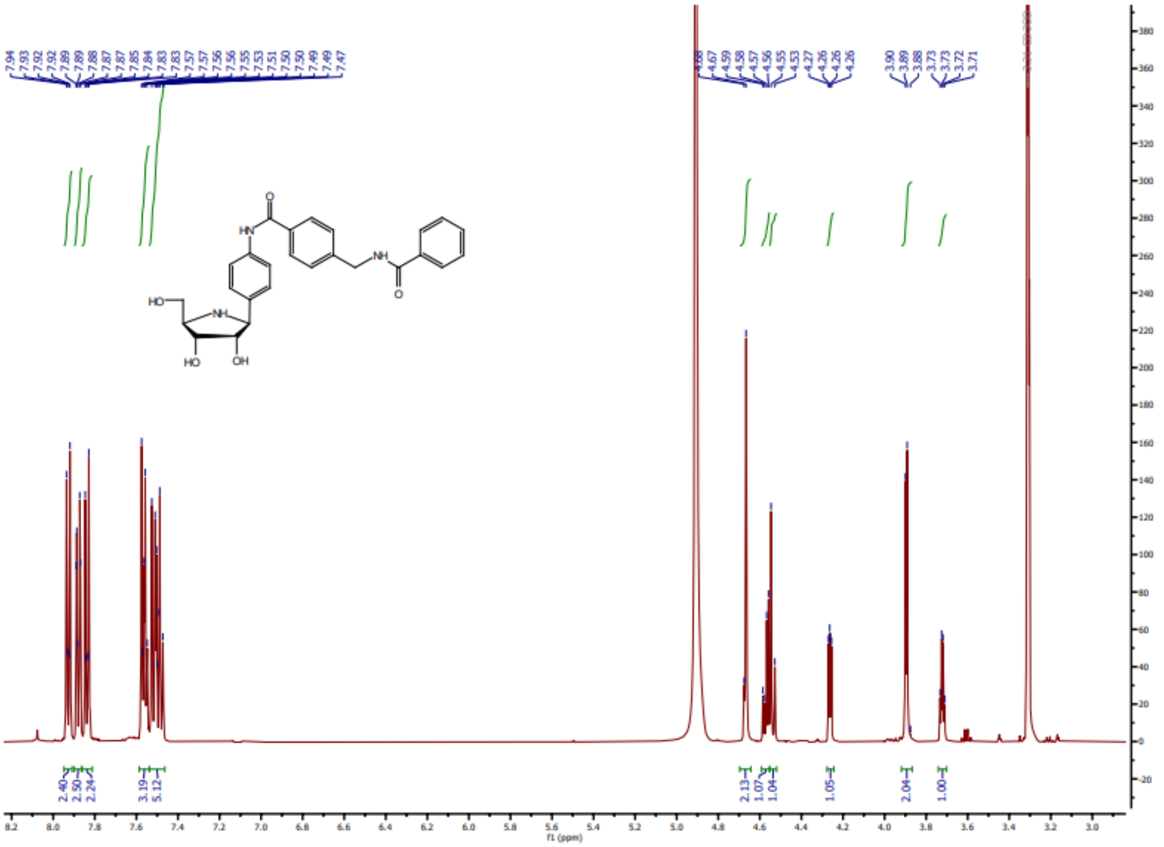

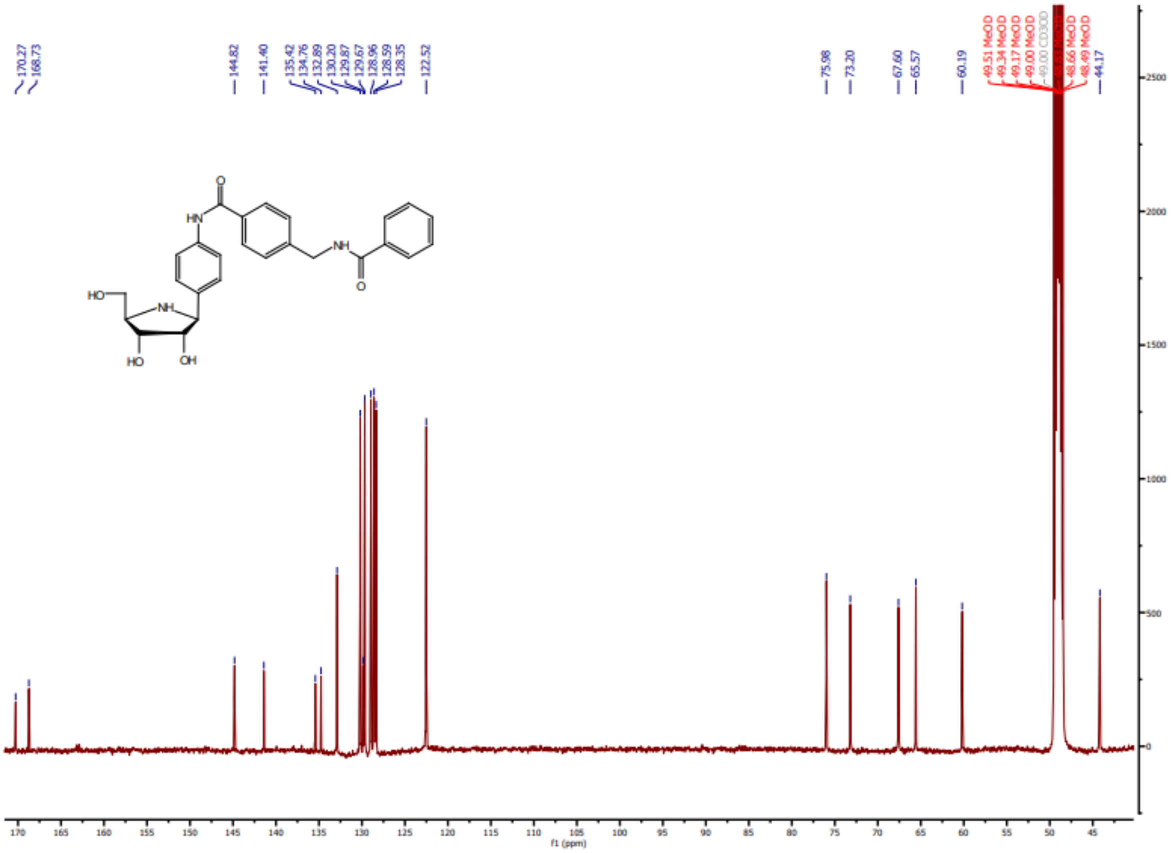

Compound **26**

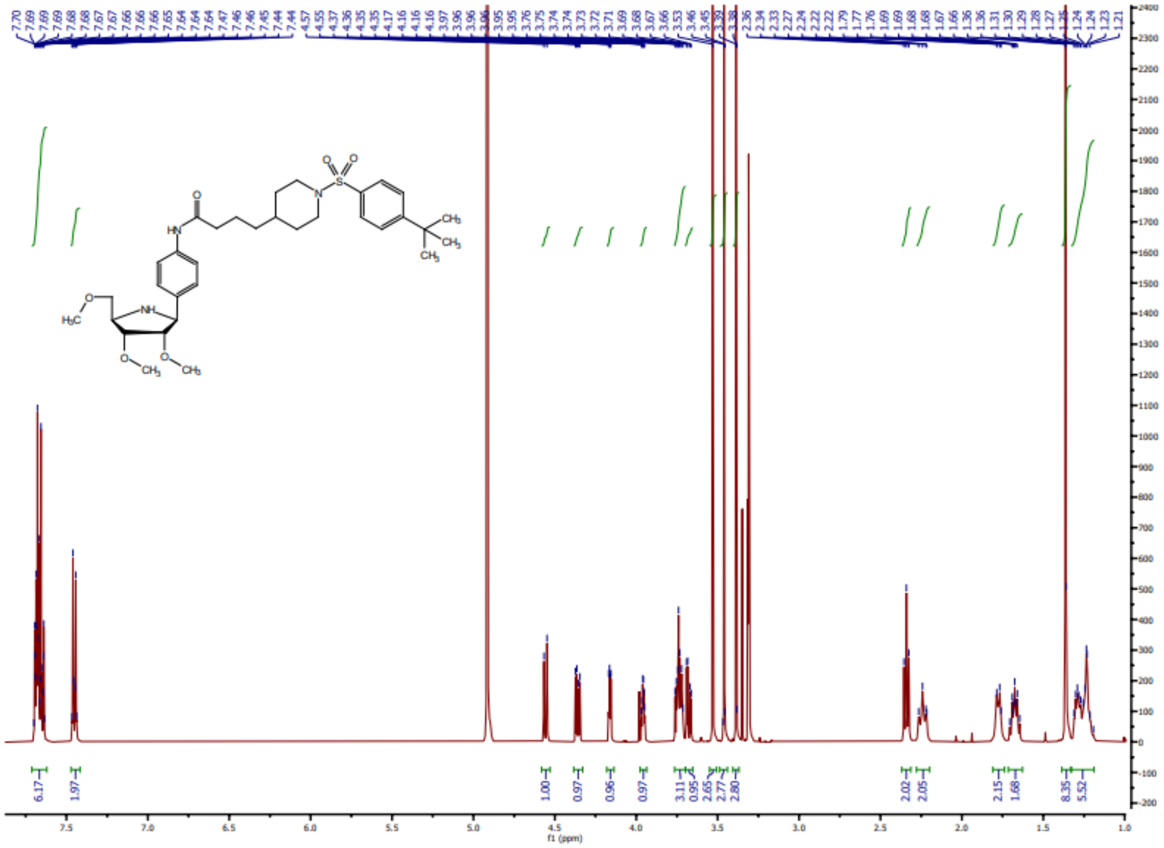

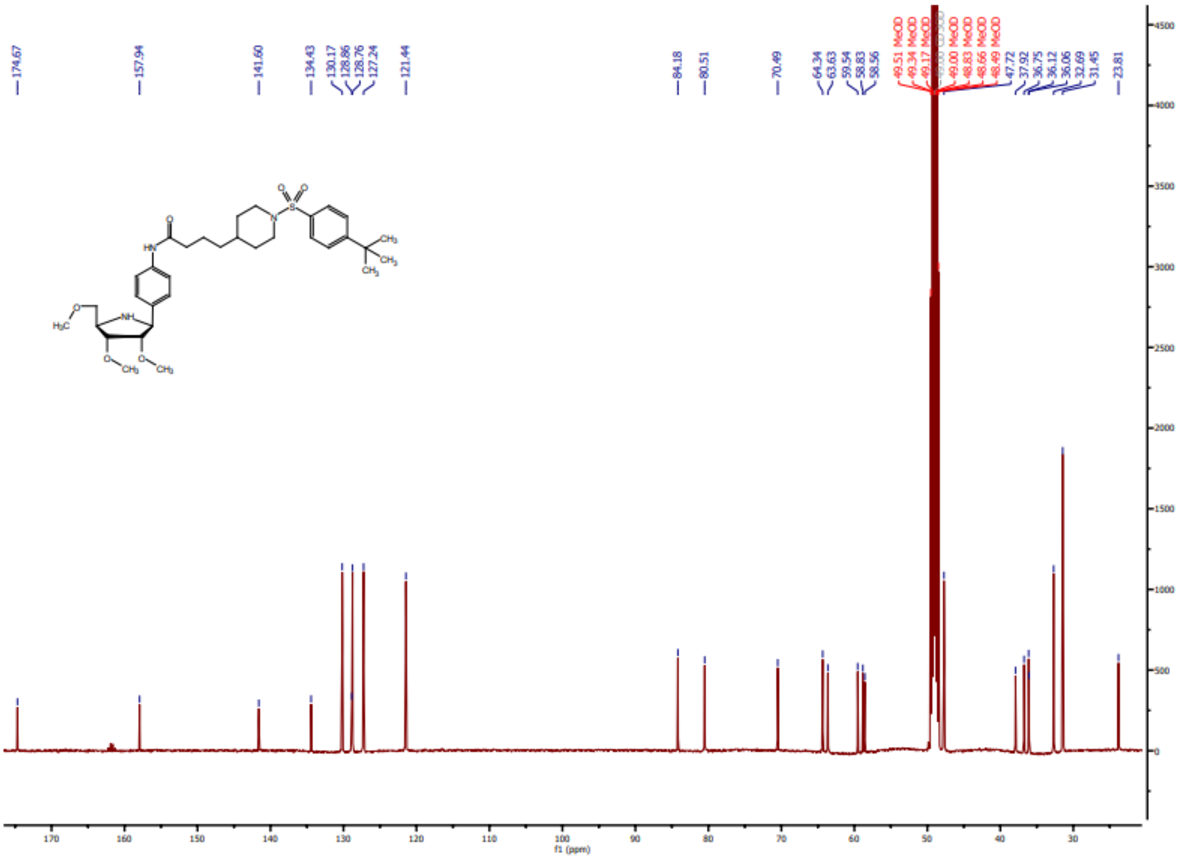

Compound **27**

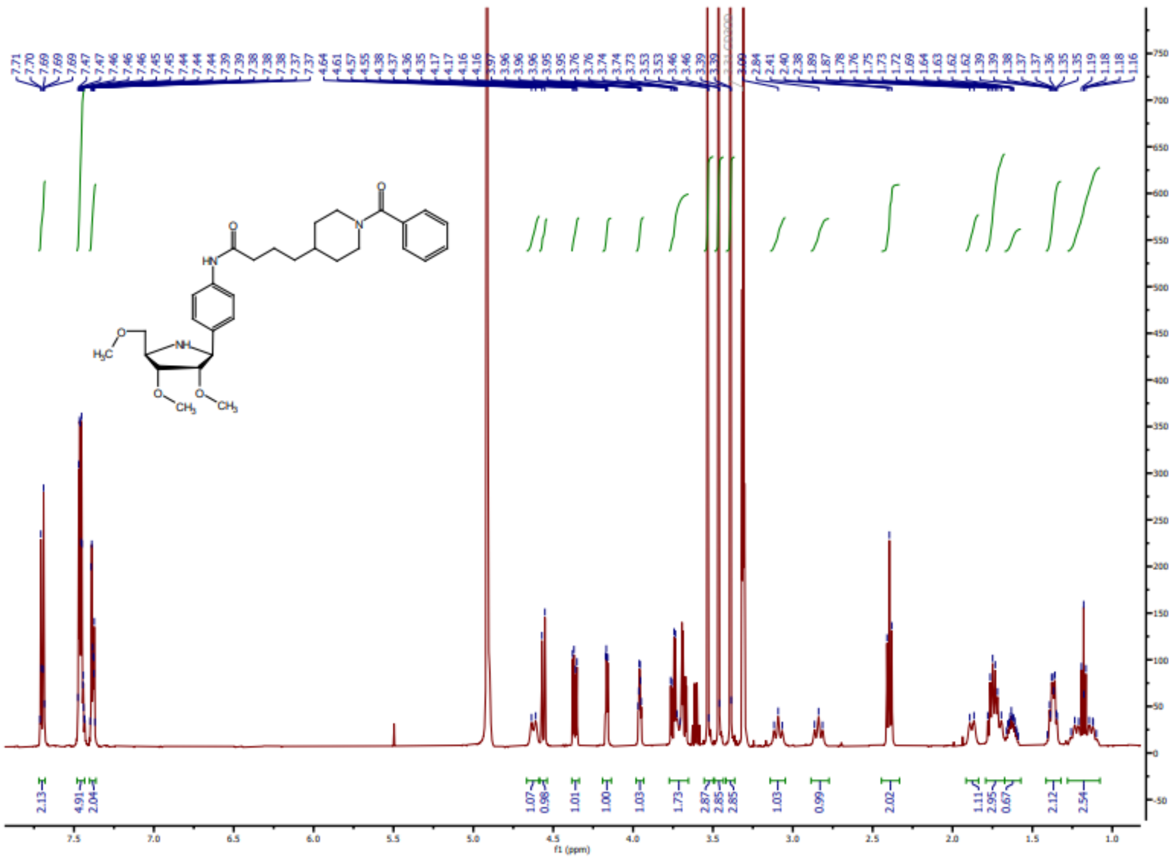

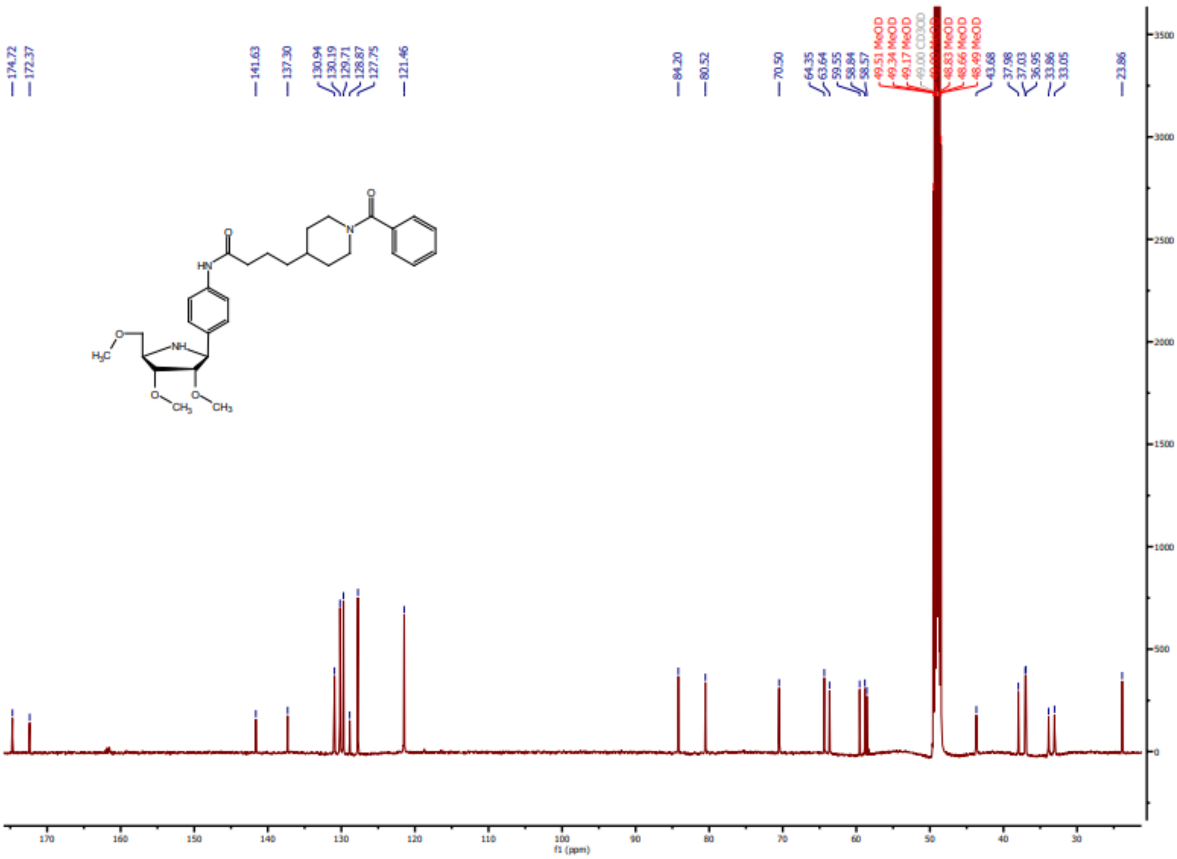

Compound **28**

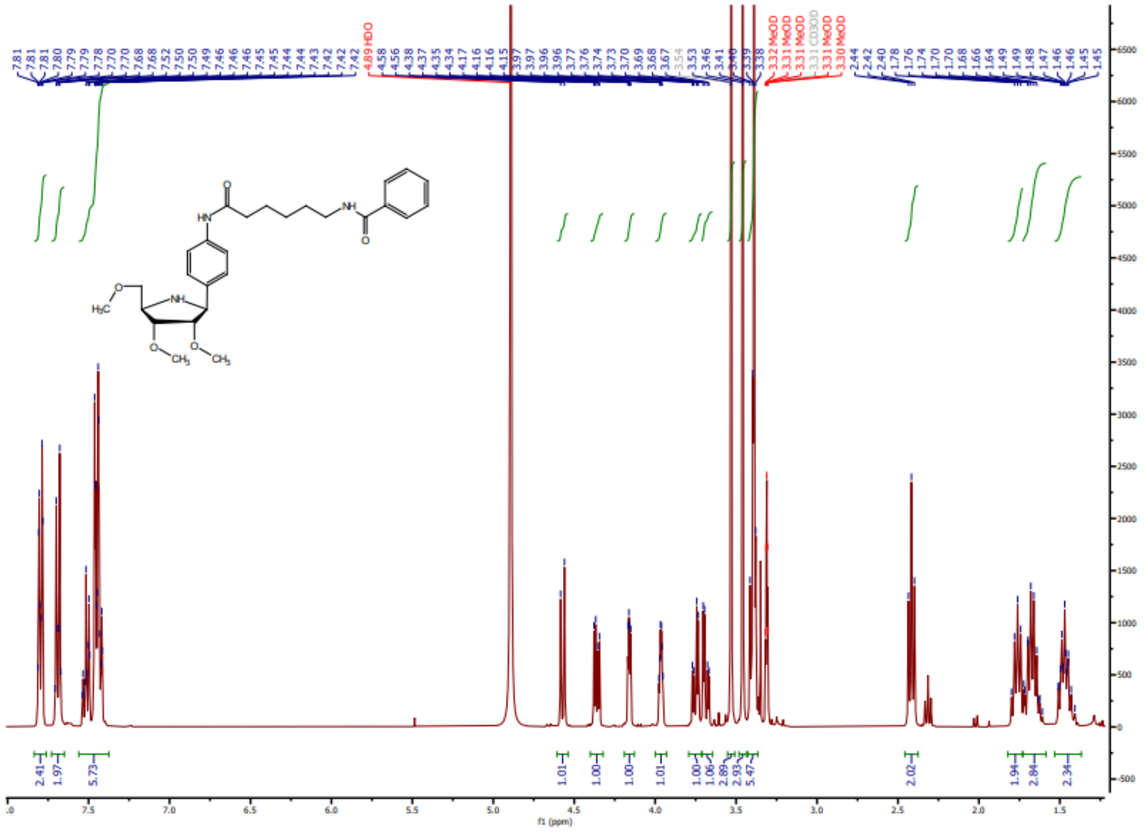

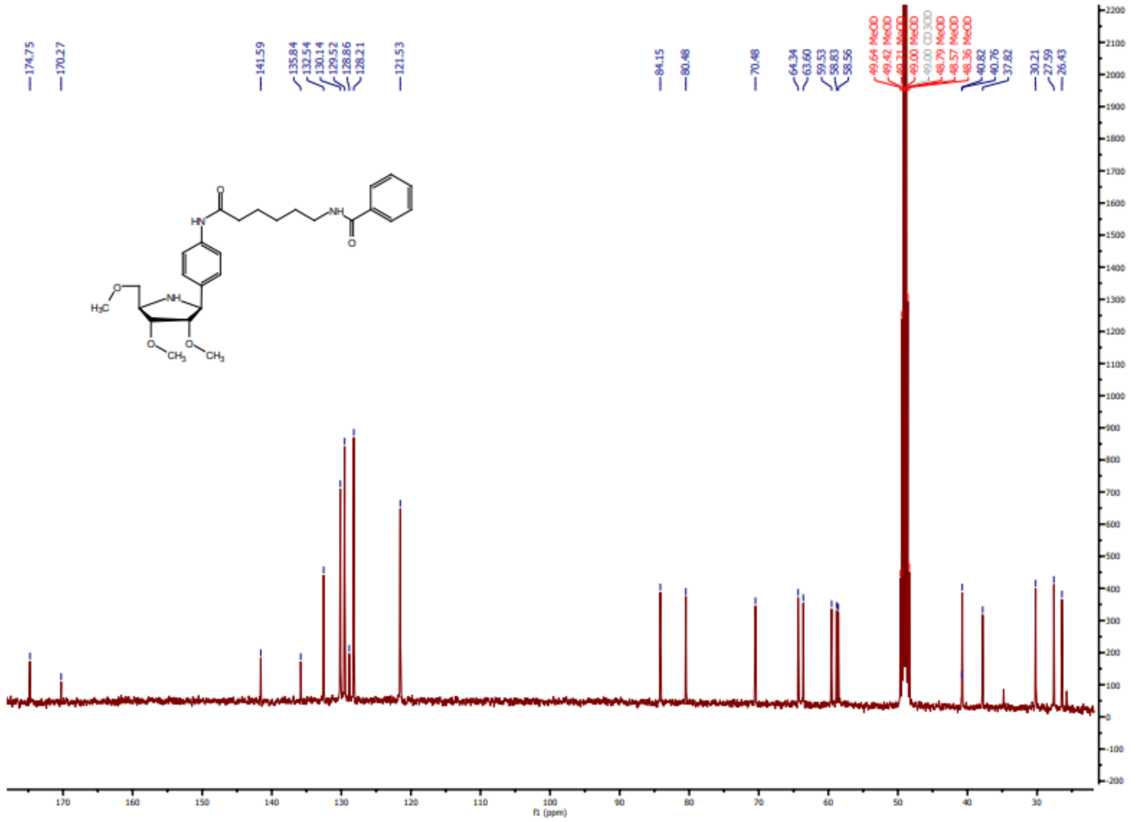

Compound **29**

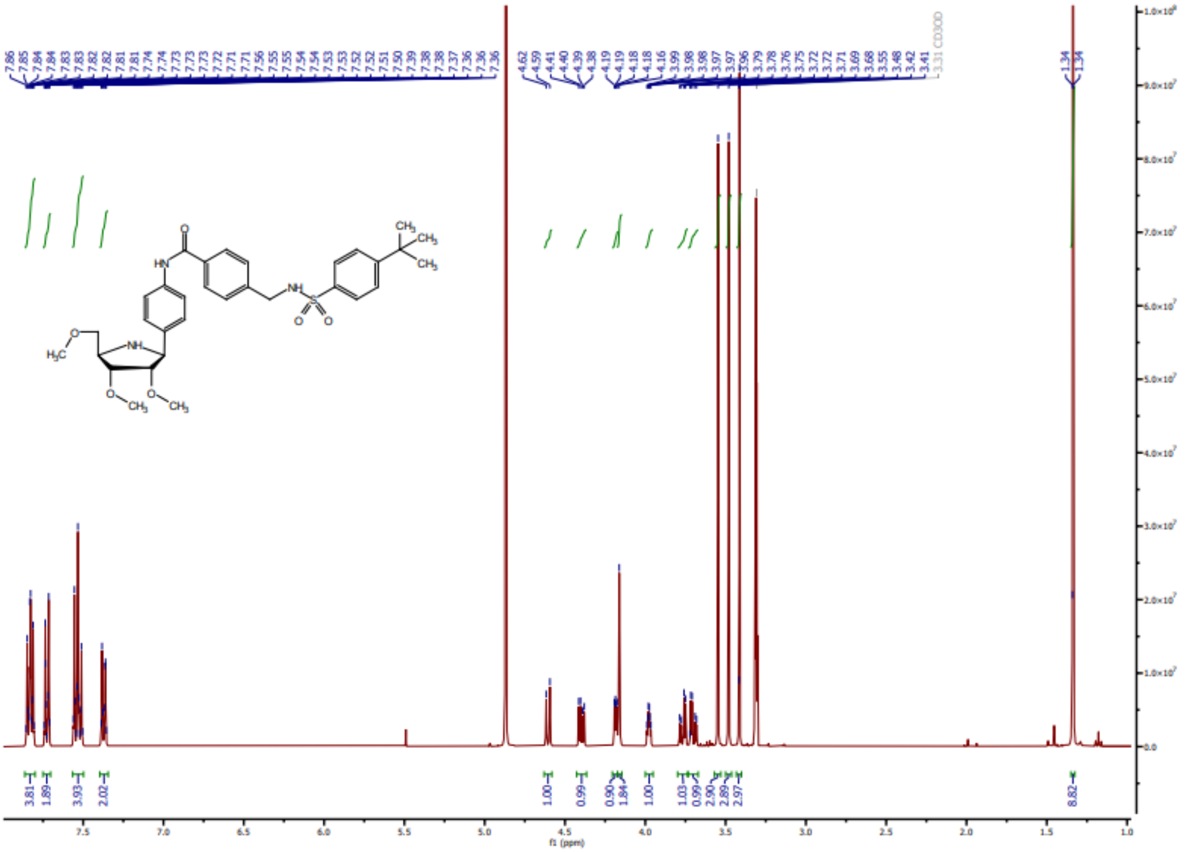

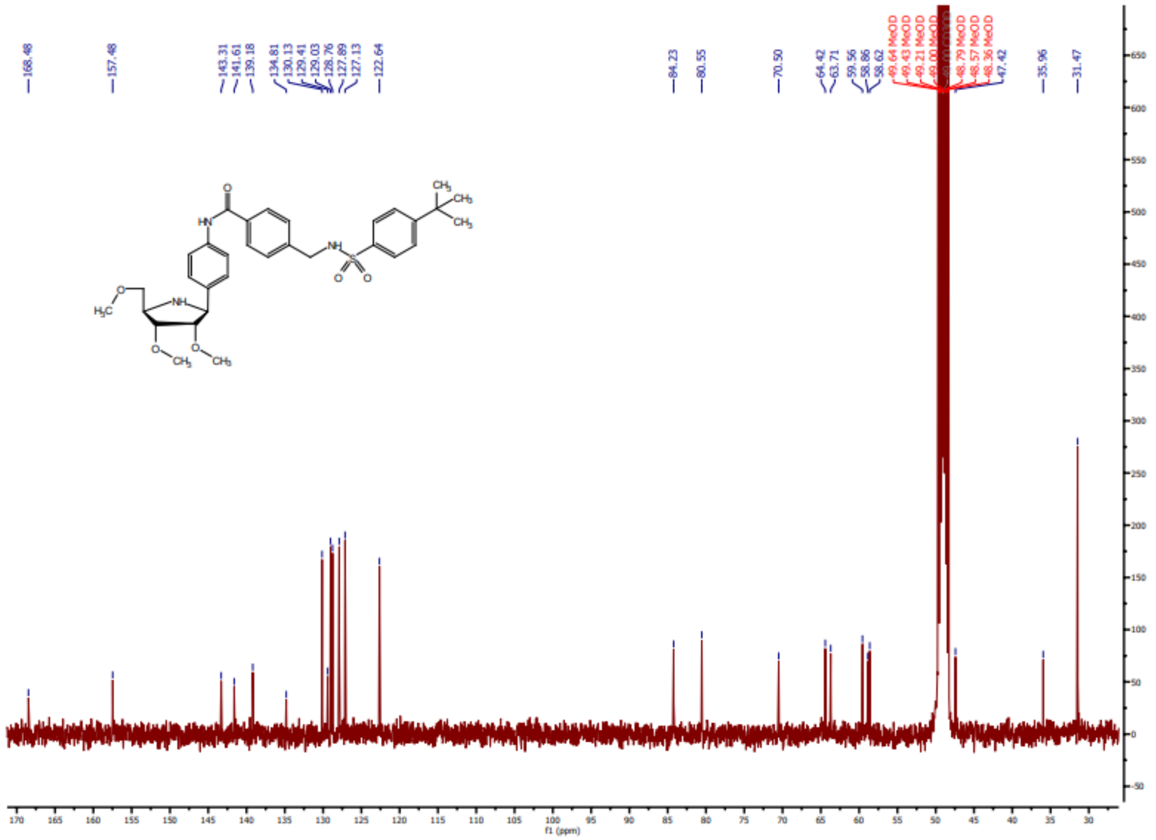

Compound **30**

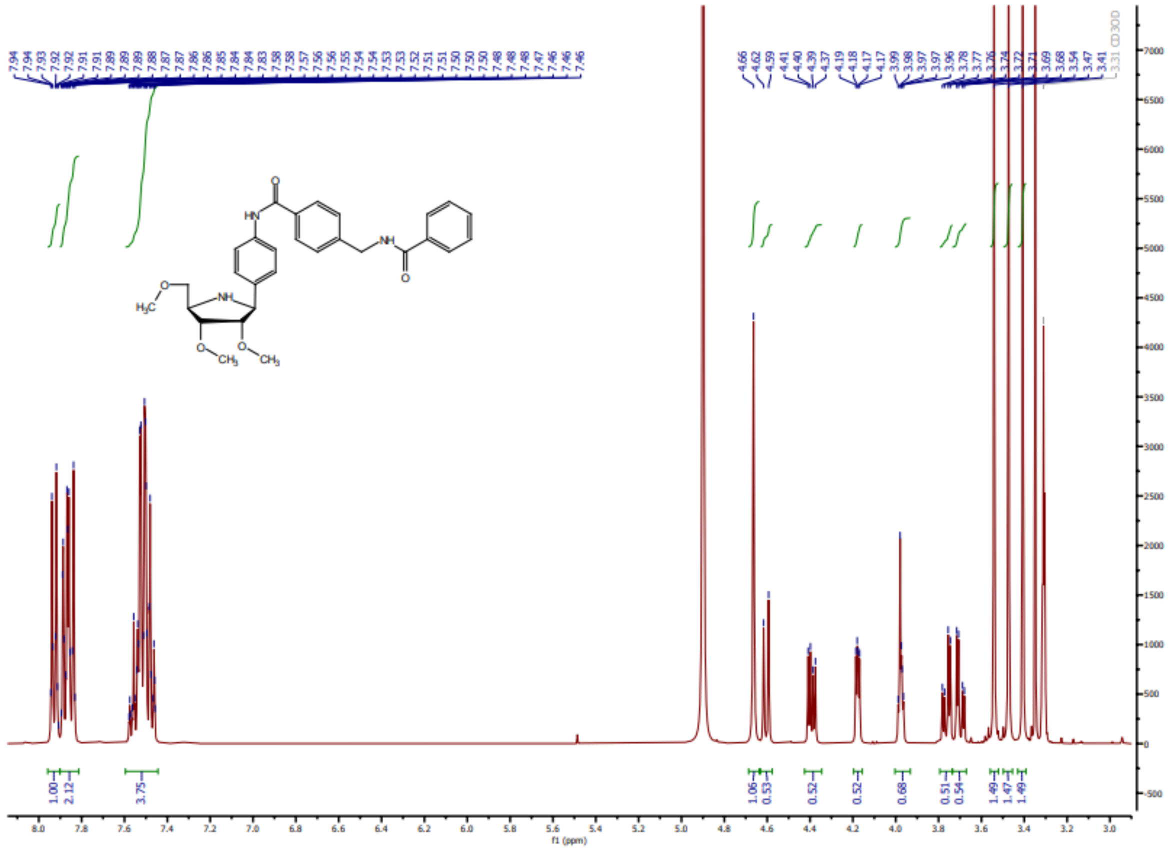
7.94 7.94 7.93 7.92 7.92 7.91 7.91 7.89 7.89 7.89 7.88 7.87 7.87 7.86 7.86 7.85 7.84 7.84 7.83 7.58 7.58 7.57 7.56 7.56 7.55 7.54 7.54 7.53 7.53 7.52 7.51 7.51 7.50 7.50 7.50 7.48 7.48 7.48 7.47 7.46 7.46 7.46
4.66 4.62 4.59 4.41 4.40 4.39 4.37 4.19 4.18 4.17 4.17 3.99 3.98 3.97 3.97 3.96 3.78 3.77 3.76 3.74 3.72 3.71 3.69 3.68 3.54 3.47 3.41 3.31 CD3OD
HN
NH
O
H3C
O
CH3
CH3
1.00
2.12
3.75
1.06
0.53
0.52
0.52
0.68
0.51
0.54
1.49
1.47
1.49
f1 (ppm)

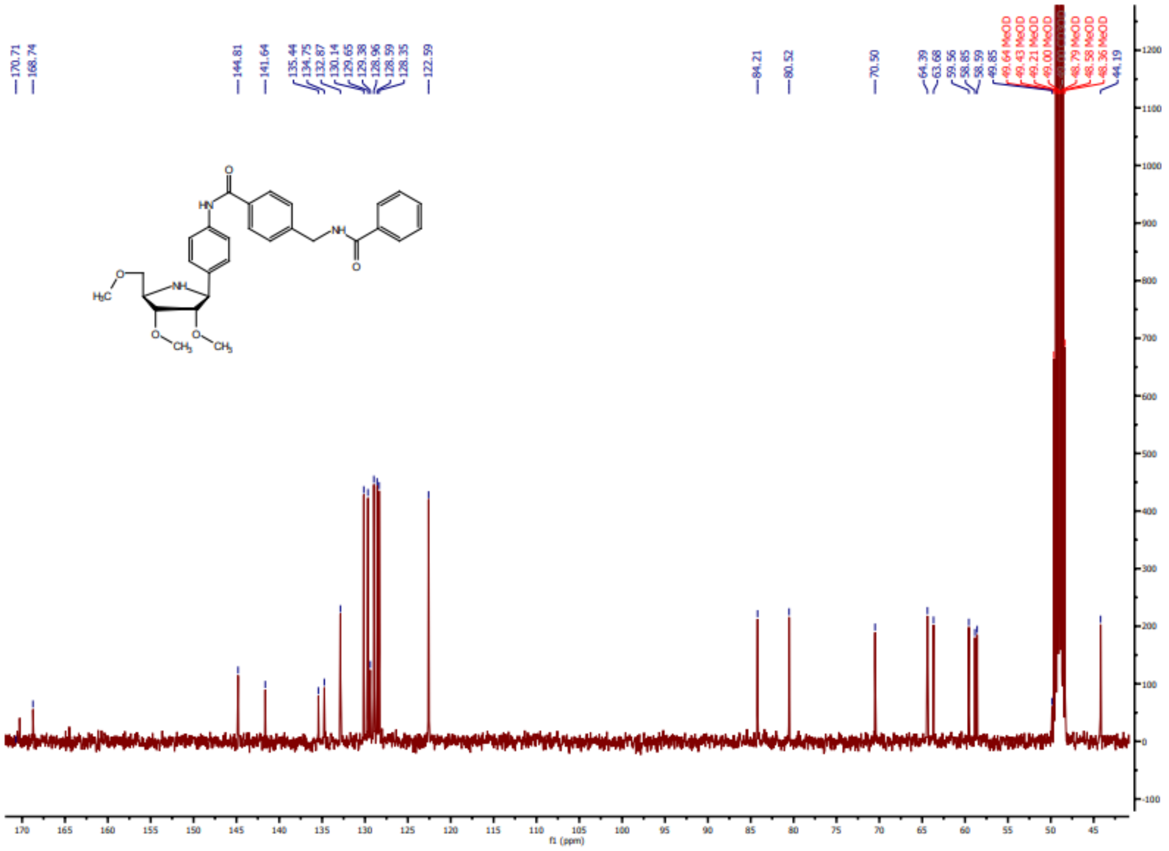
170.71
168.74
144.81
141.64
135.44 134.75 132.87 130.14 129.65 129.38 128.96 128.59 128.35
122.59
84.21
80.52
70.50
64.39 63.68 59.56 58.85 58.59 49.85
49.64 MeOD 49.43 MeOD 49.21 MeOD 49.00 MeOD 48.79 MeOD 48.58 MeOD 48.36 MeOD
44.19
HN
NH
O
H3C
CH3
CH3
f1 (ppm)

Compound **32**

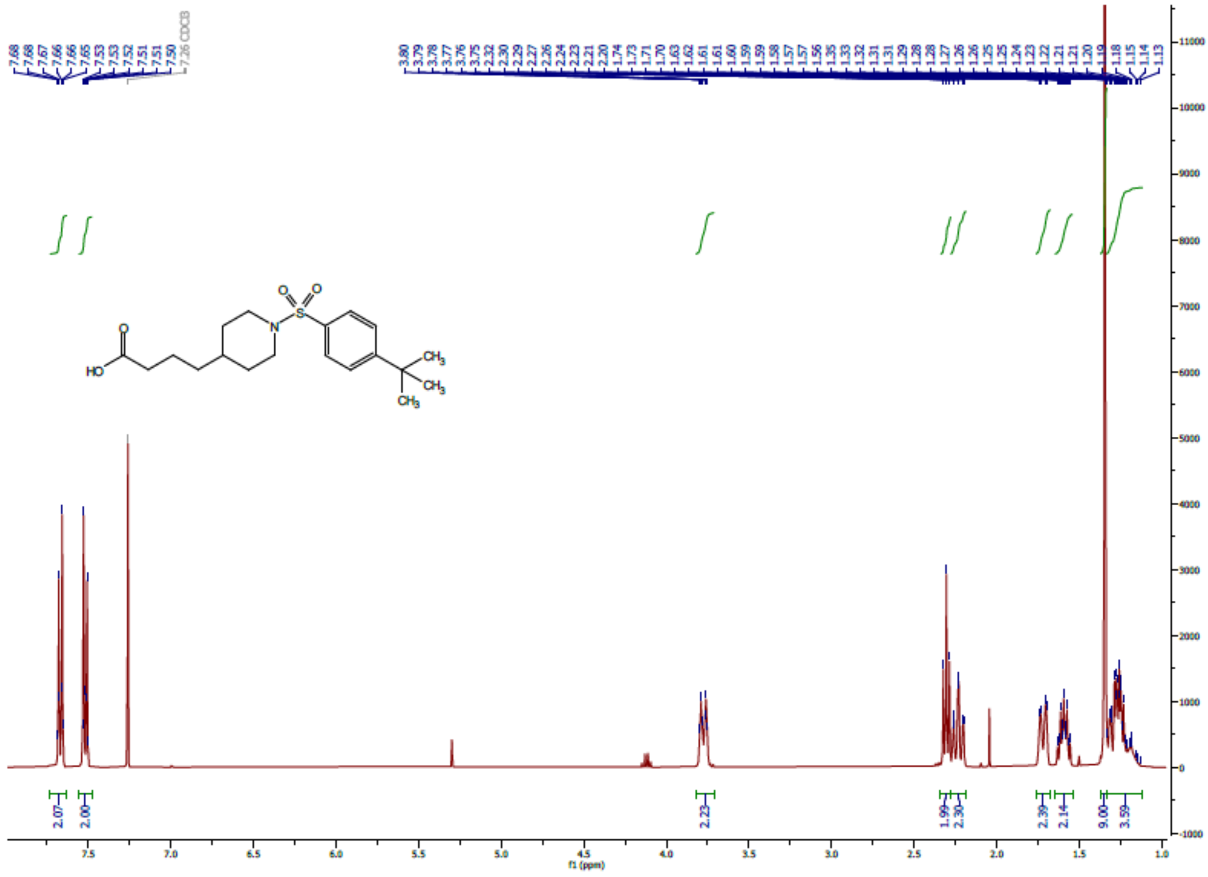

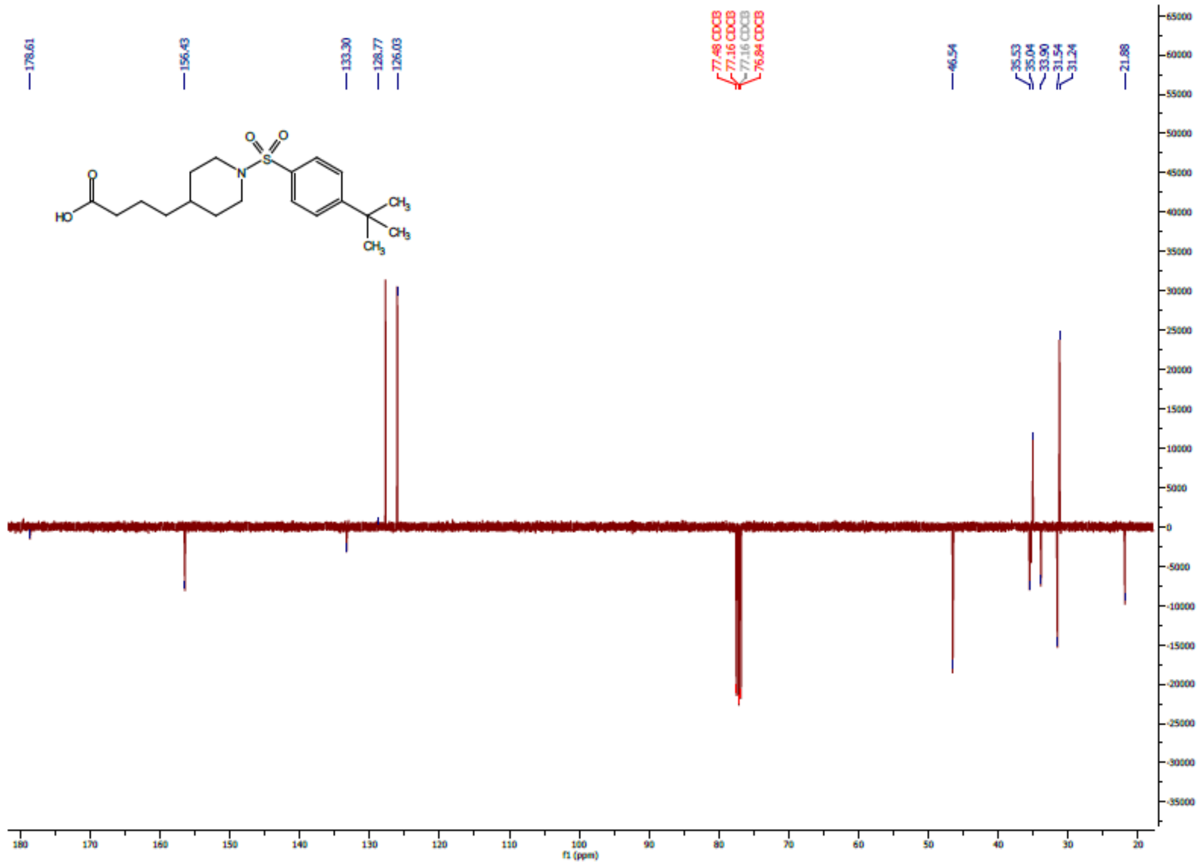

Compound **33**

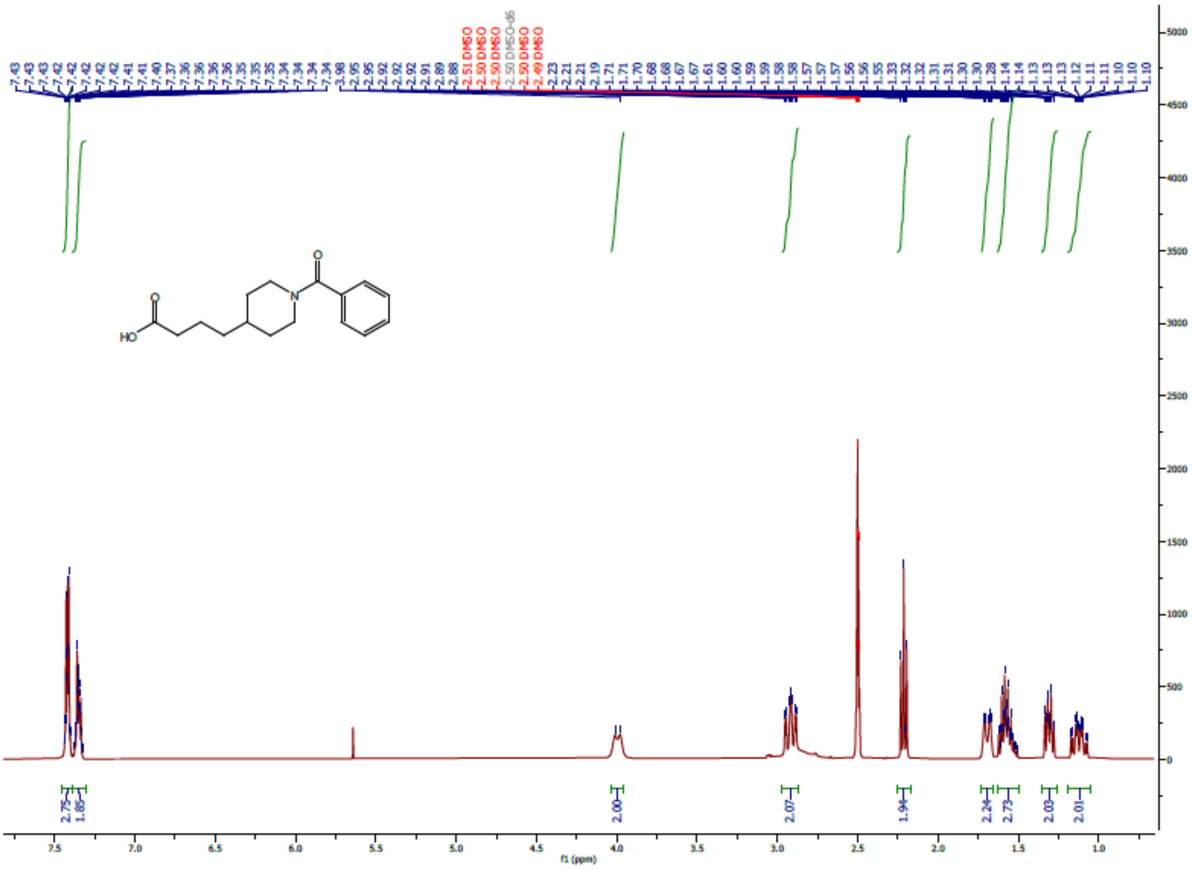

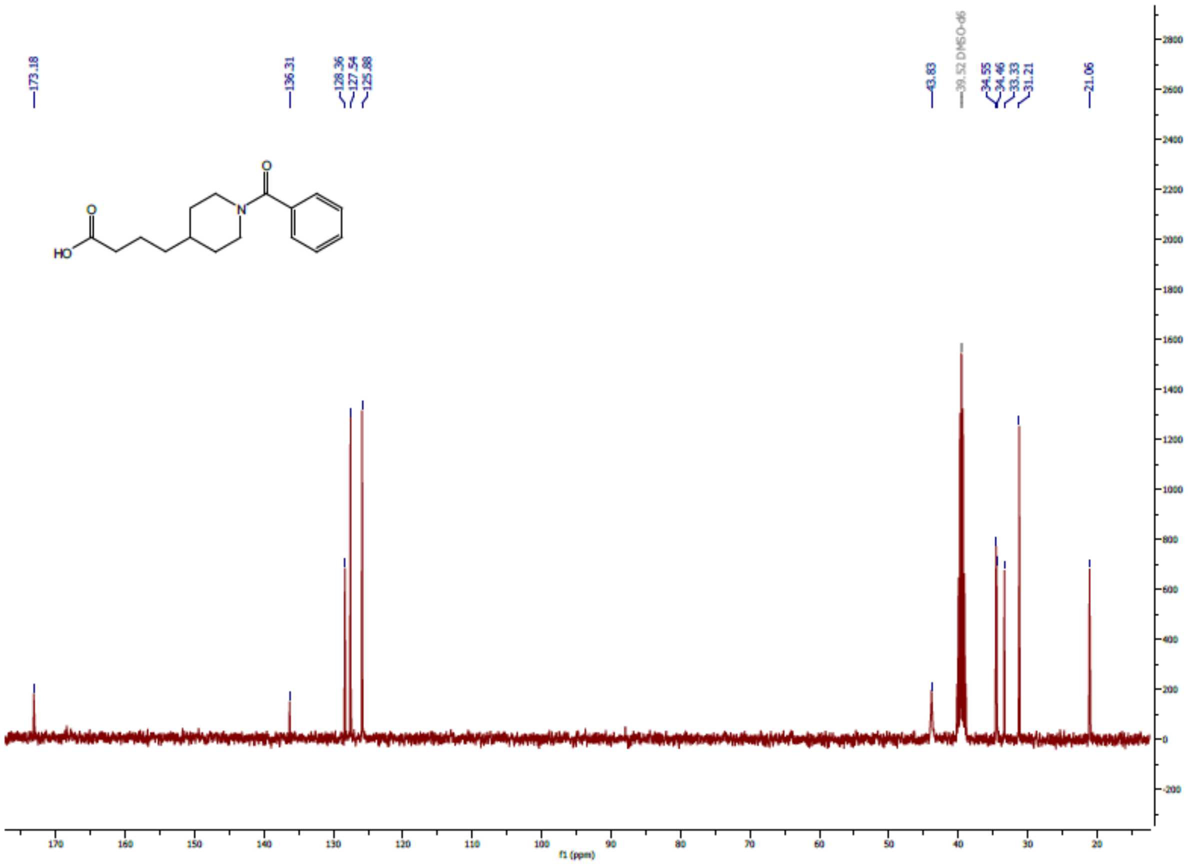

Compound **34**

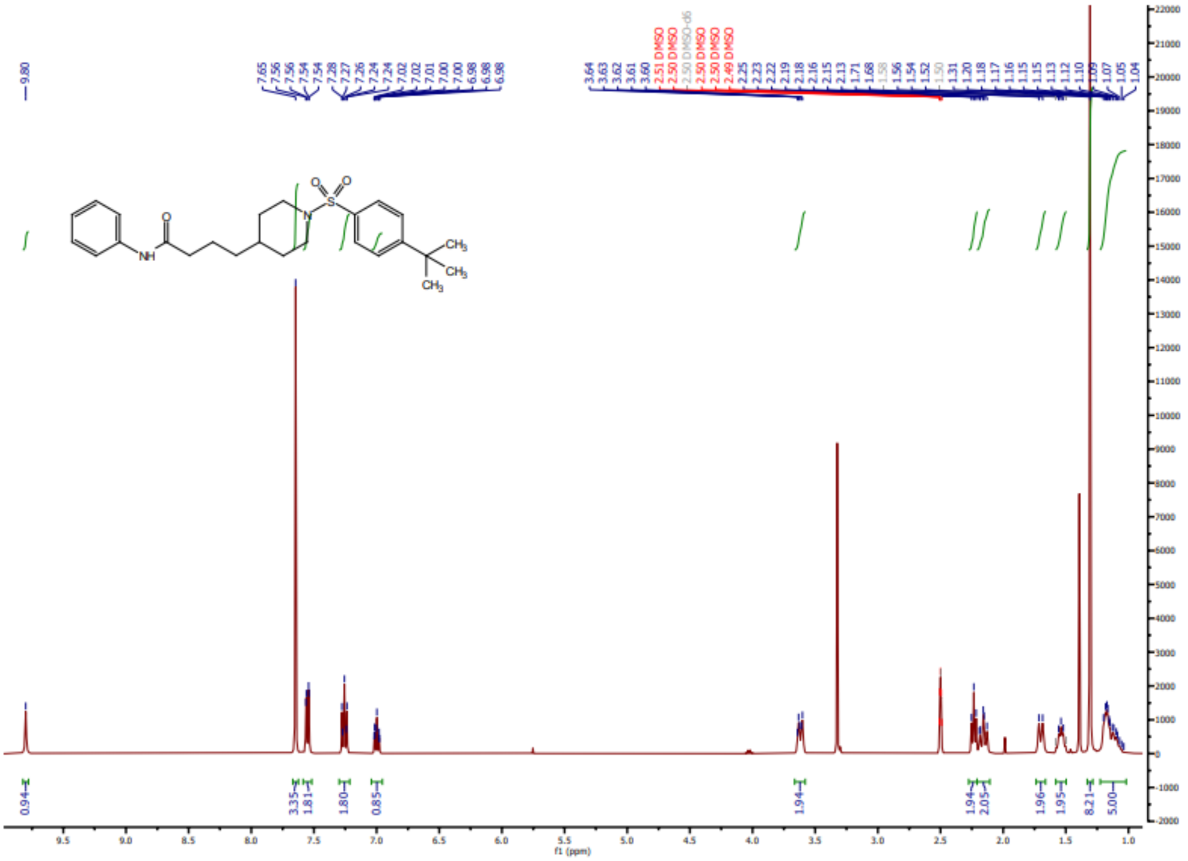

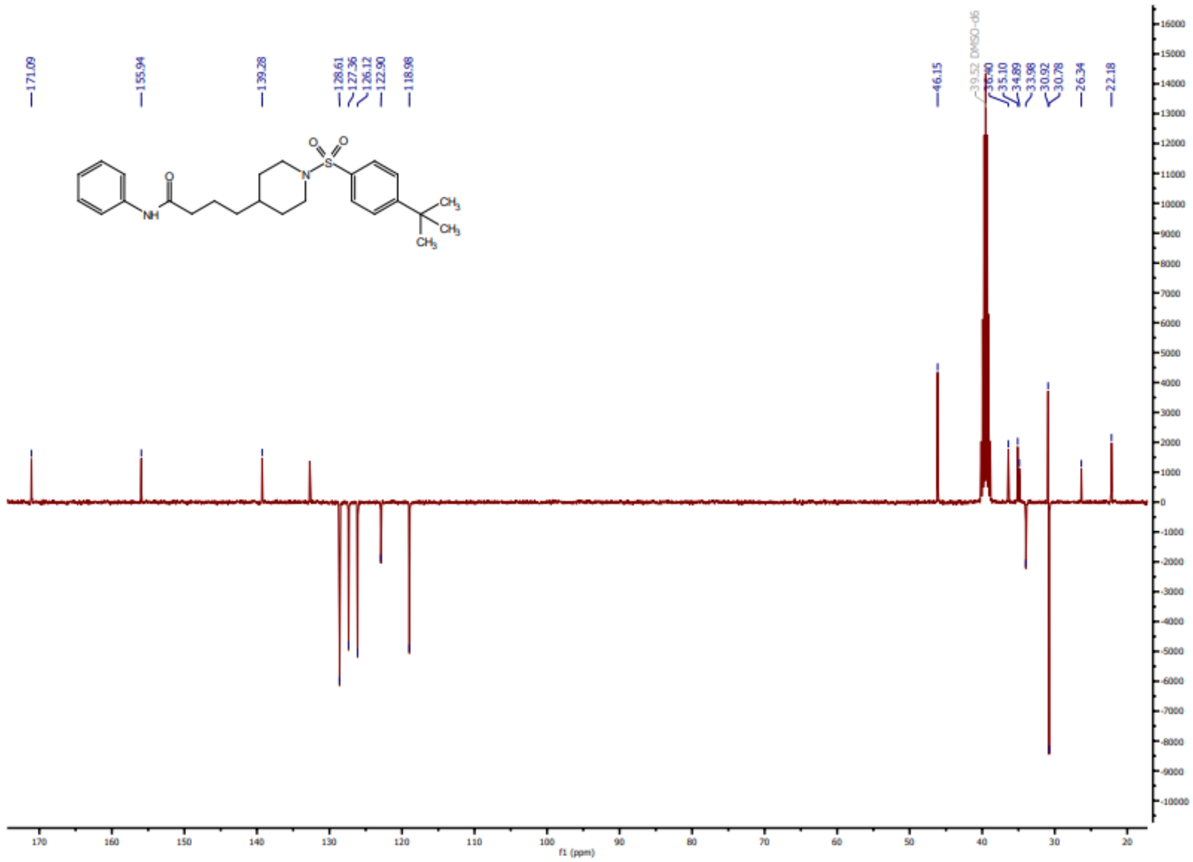

Compound **35**

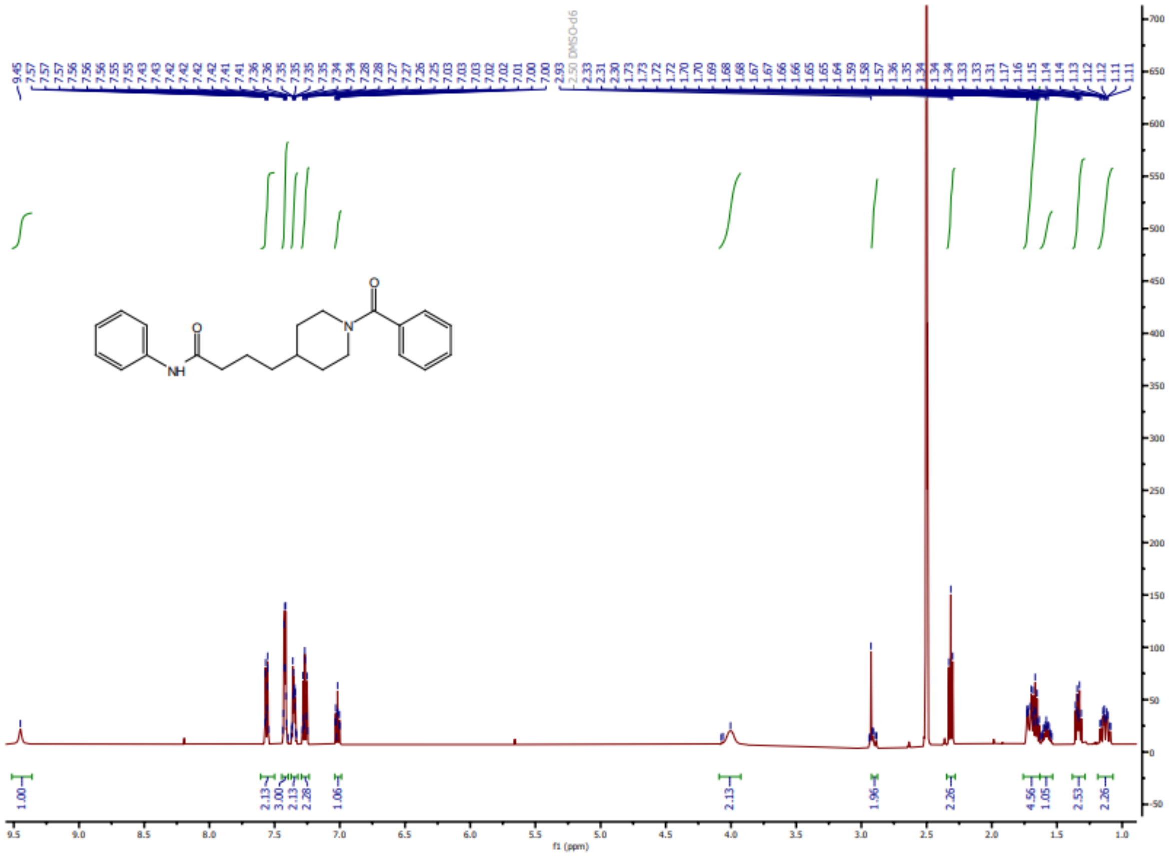

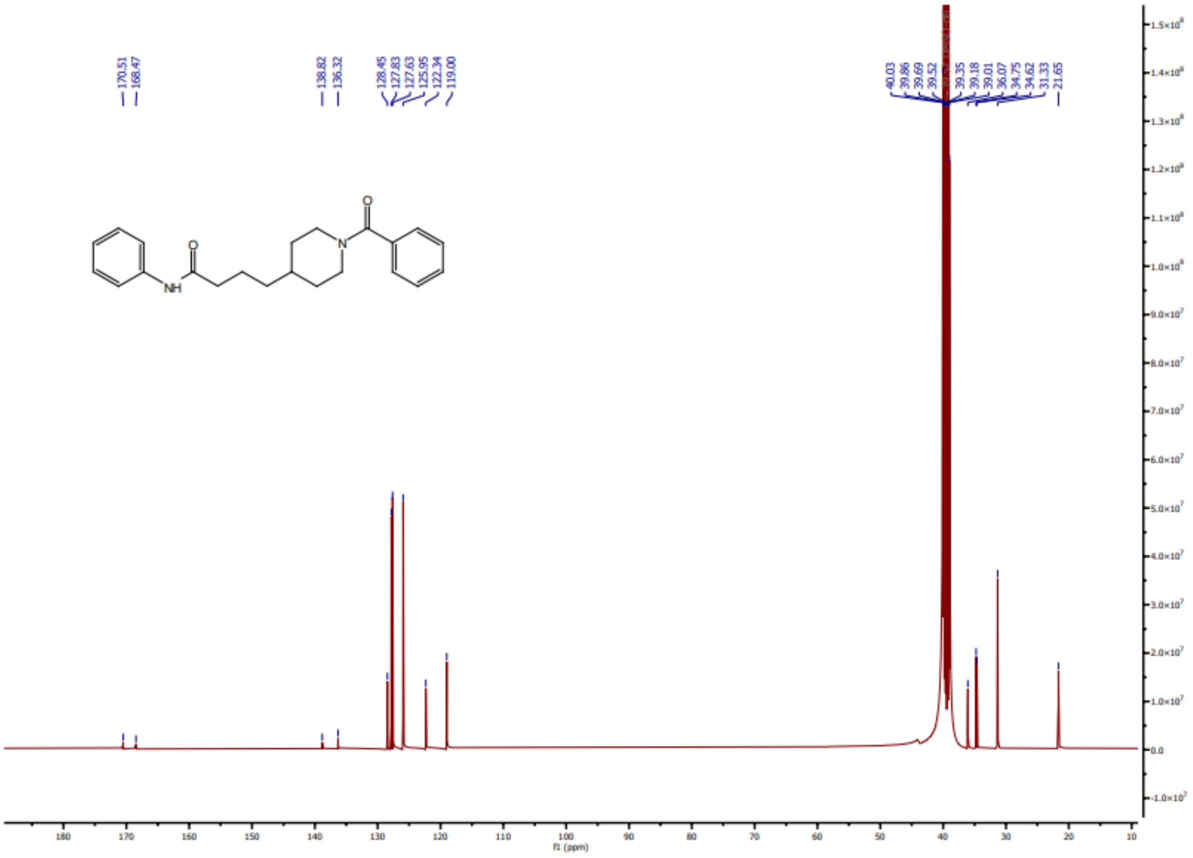

Compound **38**

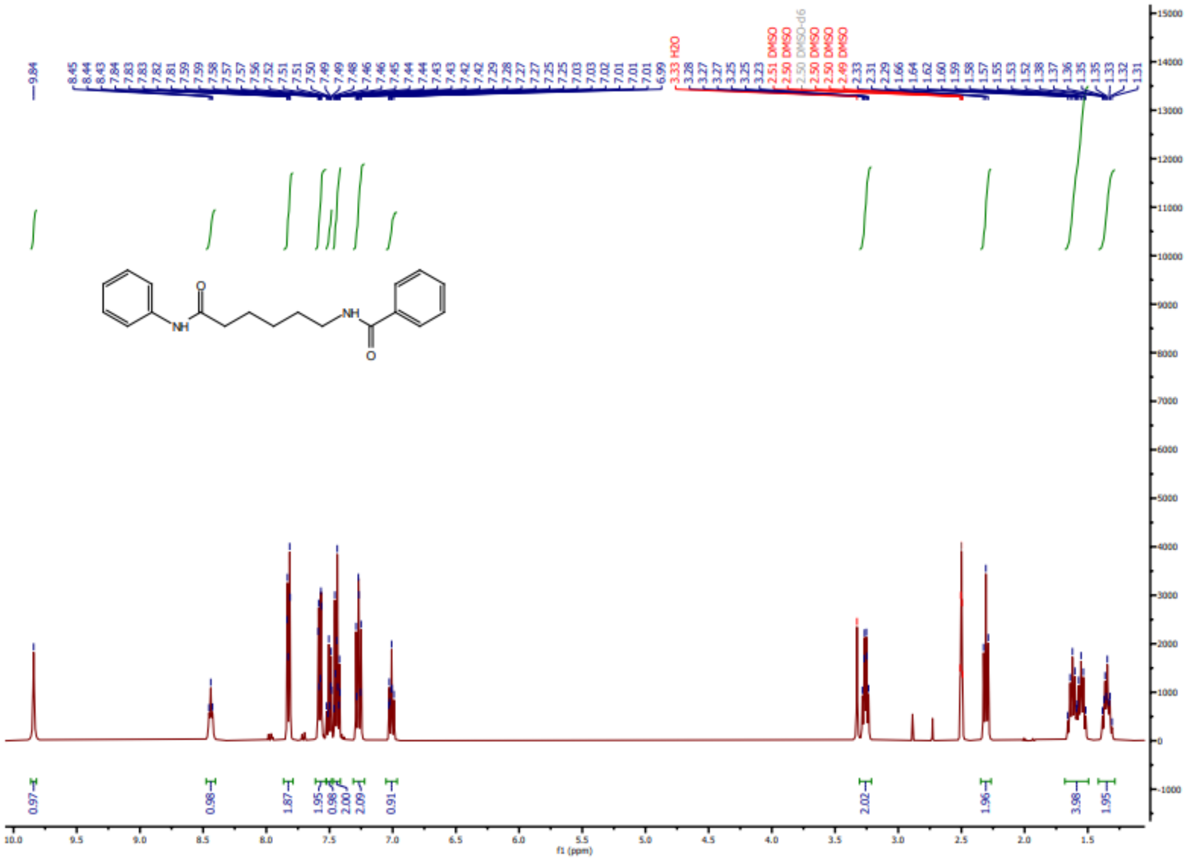

9.84
8.45 8.44 8.43 7.84 7.83 7.83 7.82 7.81 7.59 7.58 7.57 7.57 7.56 7.52 7.51 7.51 7.50 7.49 7.49 7.48 7.46 7.46 7.45 7.44 7.44 7.43 7.43 7.42 7.42 7.29 7.28 7.27 7.27 7.25 7.25 7.03 7.02 7.01 7.01 6.99
3.33 H2O
3.28 3.27 3.25 3.25 3.23
2.51 DMSO
2.50 DMSO
2.50 DMSO-d6
2.50 DMSO
2.50 DMSO
2.49 DMSO
2.33 2.31 2.29 1.66 1.64 1.62 1.60 1.59 1.58 1.57 1.55 1.53 1.52 1.38 1.37 1.36 1.35 1.35 1.33 1.32 1.31
NH
O
NH
O
0.97 0.98 1.87 1.95 0.98 2.00 2.09 0.91 2.02 1.96 3.98 1.95
f1 (ppm)

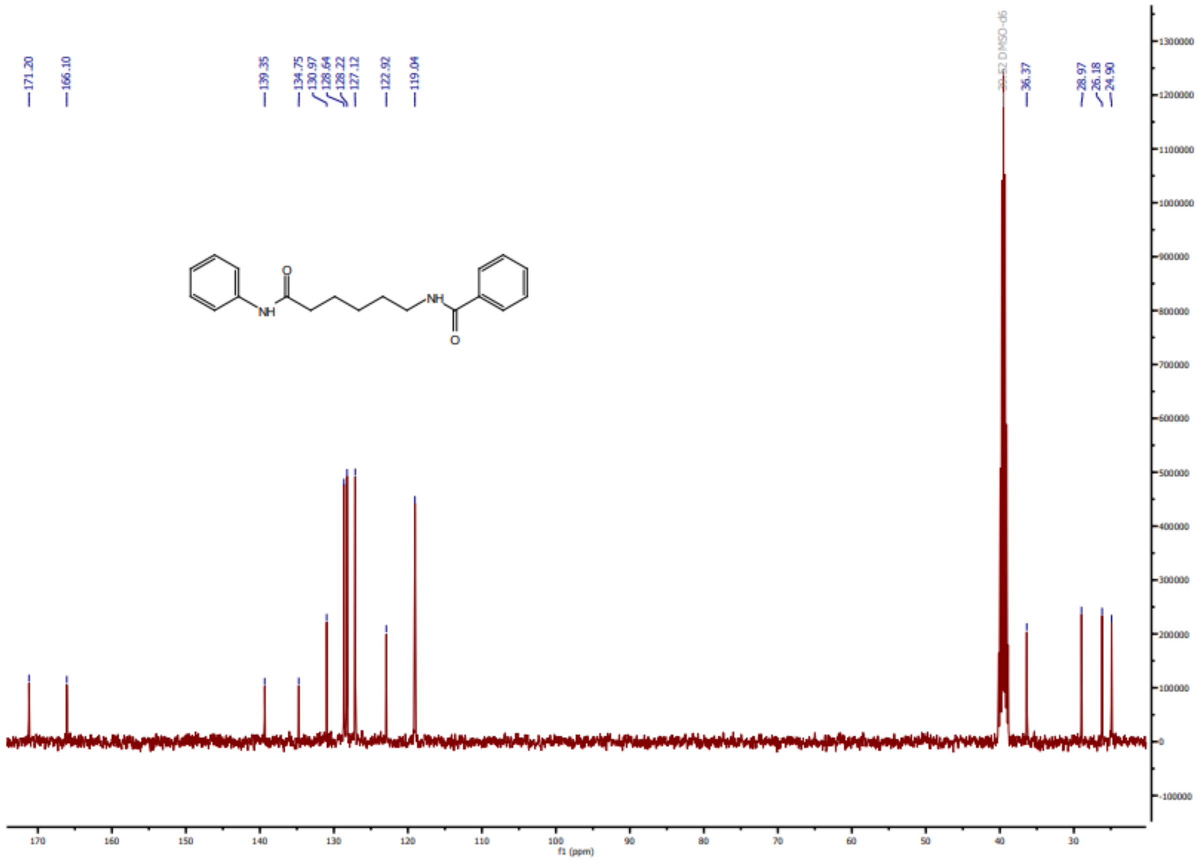

171.20
166.10
139.35
134.75
130.97
128.64
128.22
127.12
122.92
119.04
39.52 DMSO-d6
36.37
28.97
26.18
24.90
NH
O
NH
O
f1 (ppm)

Compound **42**

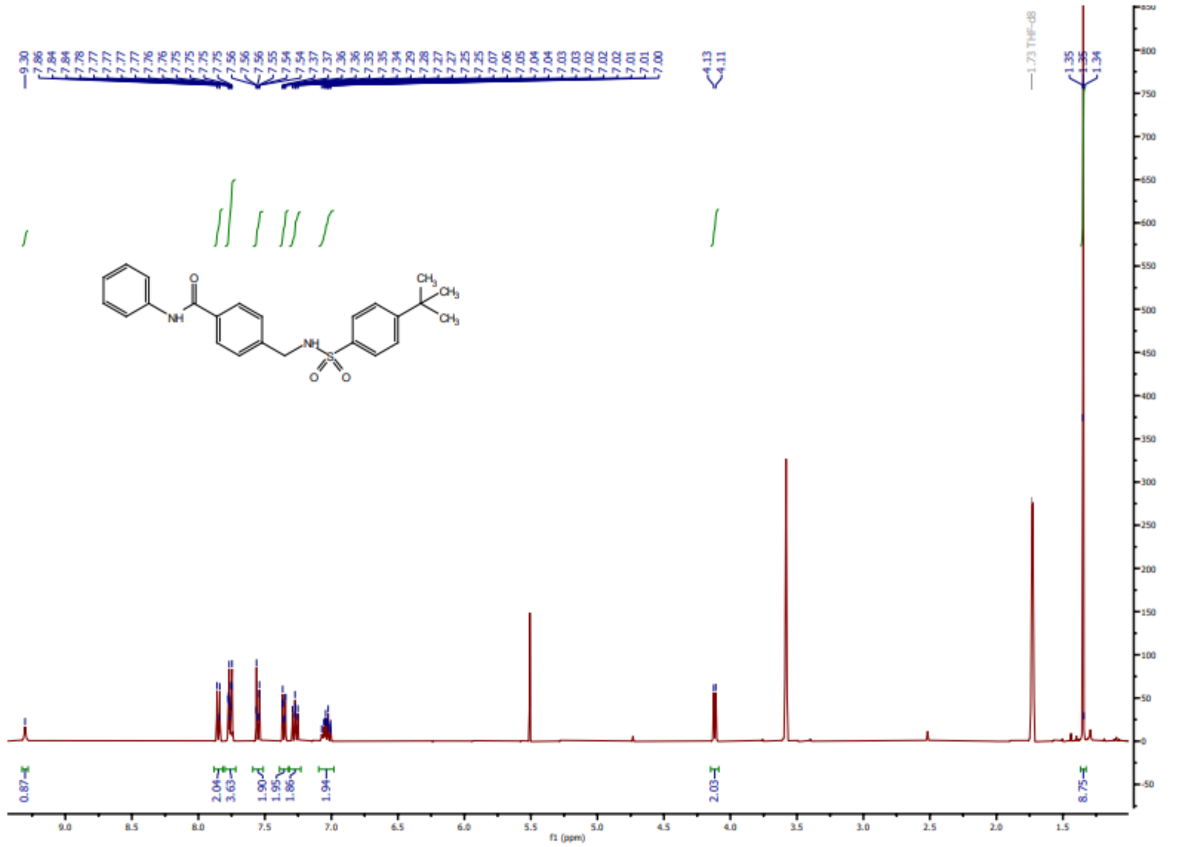

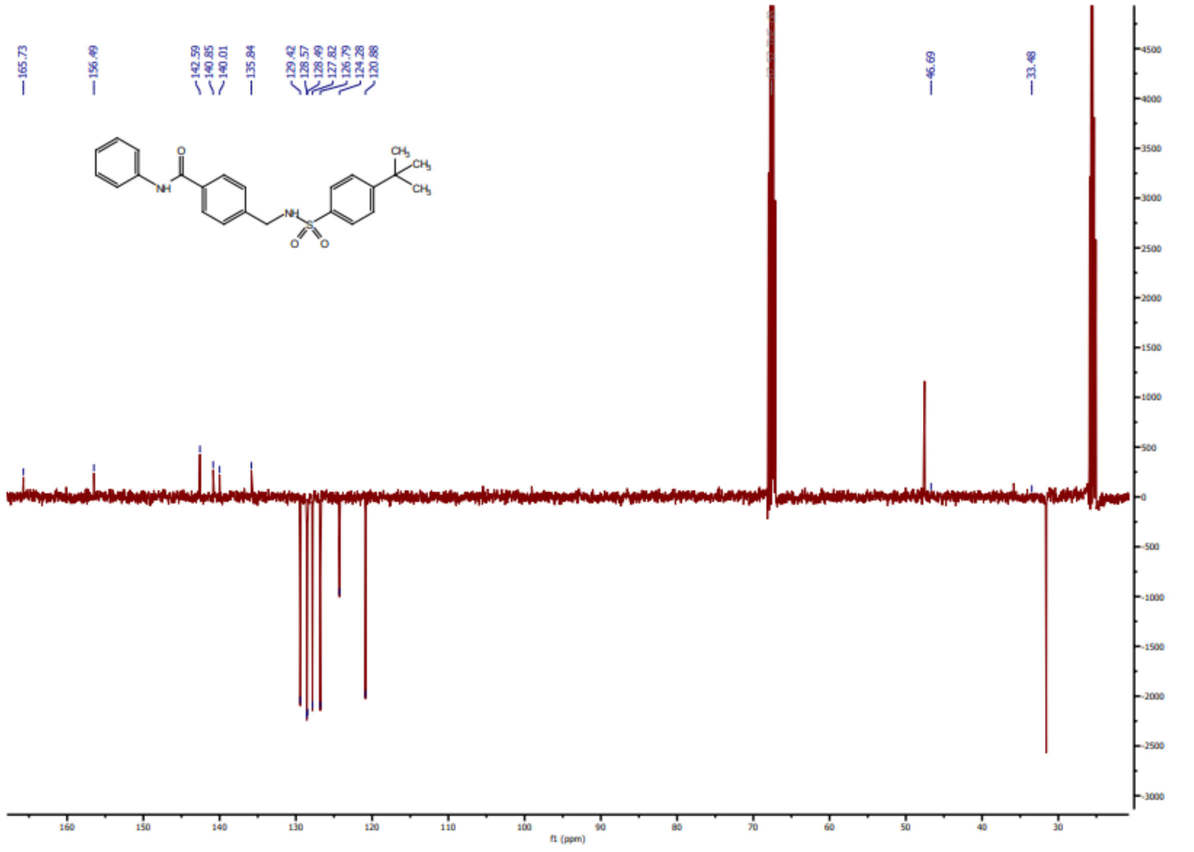

Compound **43**

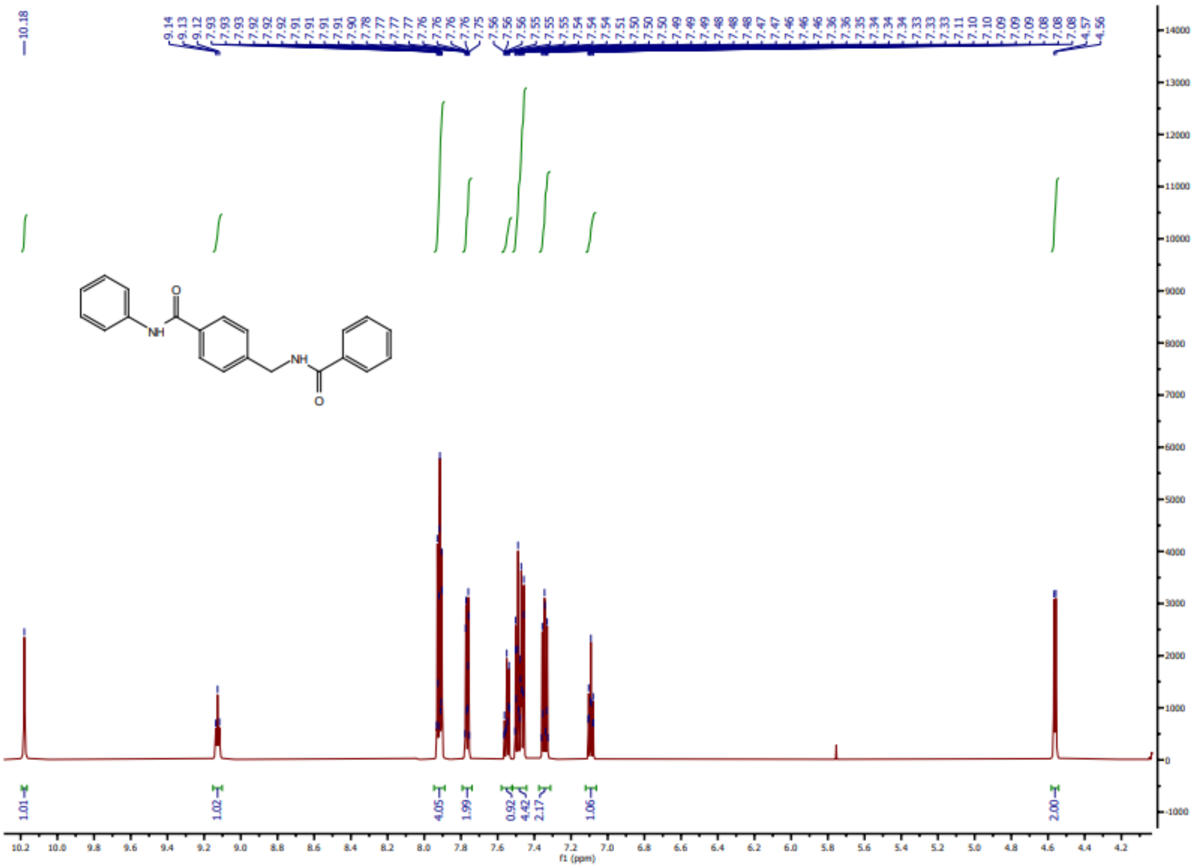
10.18
9.14
9.13
9.12
7.93
7.92
7.91
7.90
7.78
7.77
7.76
7.75
7.56
7.55
7.54
7.51
7.50
7.49
7.48
7.47
7.46
7.36
7.35
7.34
7.33
7.11
7.10
7.09
7.08
4.57
4.56
1.01
1.02
4.05
1.99
0.92
4.42
2.17
1.06
2.00
f1 (ppm)

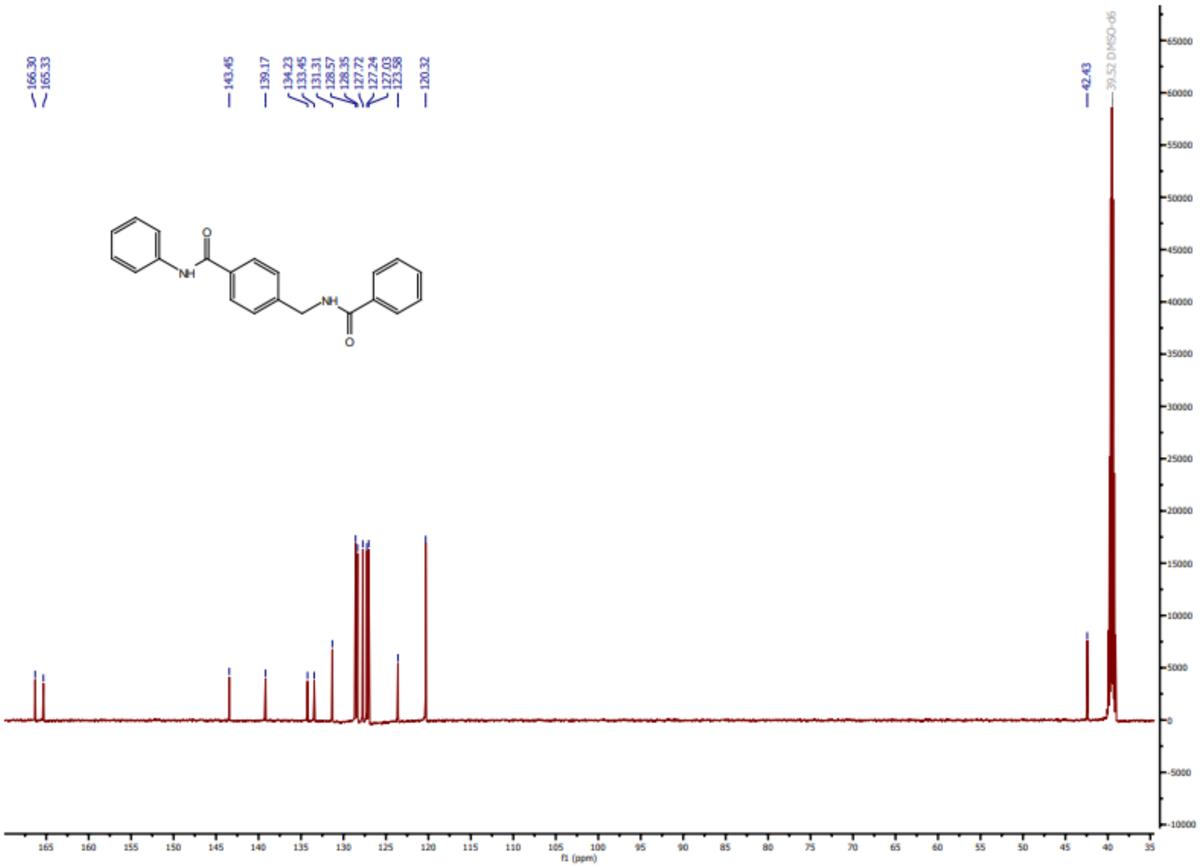
166.30
165.33
143.45
139.17
134.23
133.45
131.31
128.57
128.35
127.72
127.24
127.03
123.58
120.32
42.43
39.52 DMSO-d6
f1 (ppm)

DOCKING ANALYSIS

Compound 21

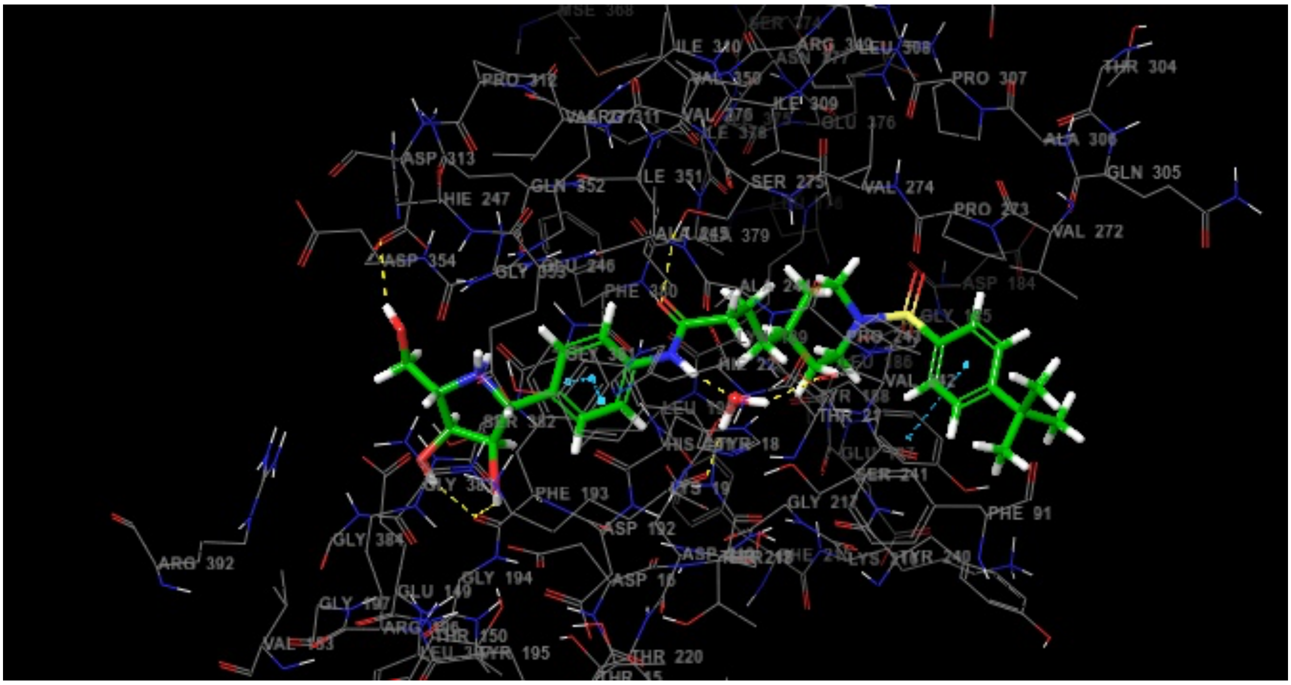

Compound 22

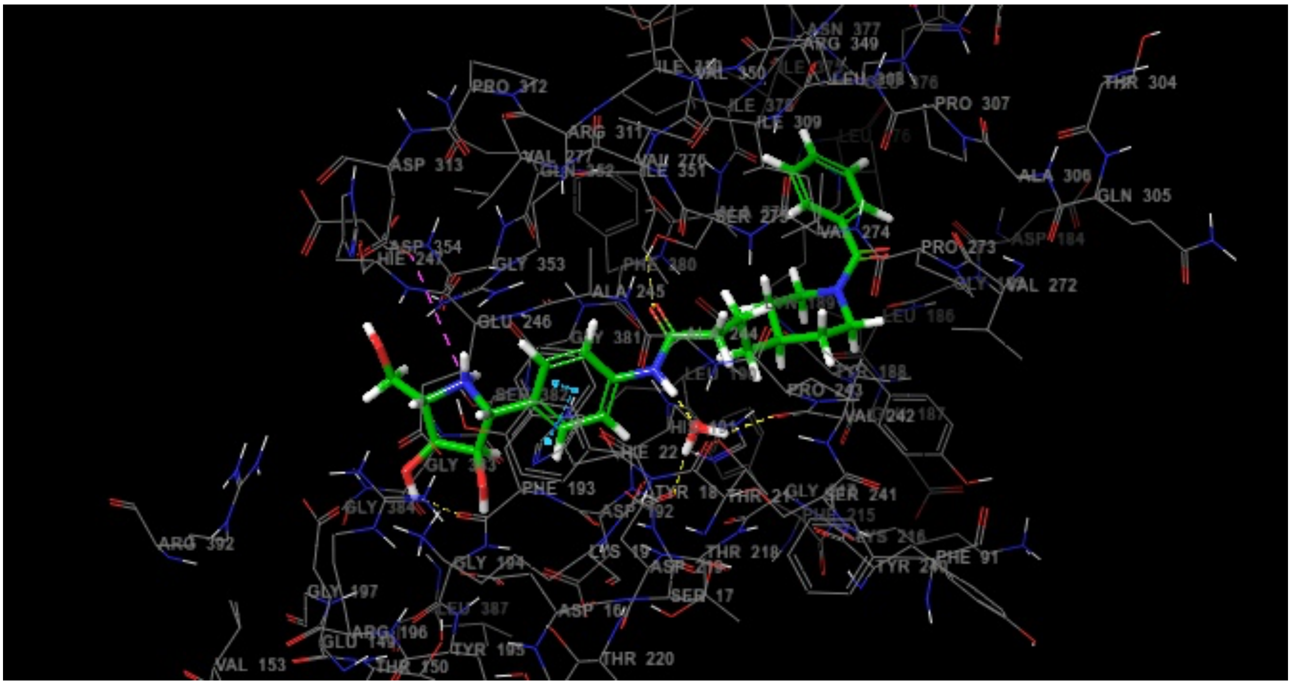

Compound 23

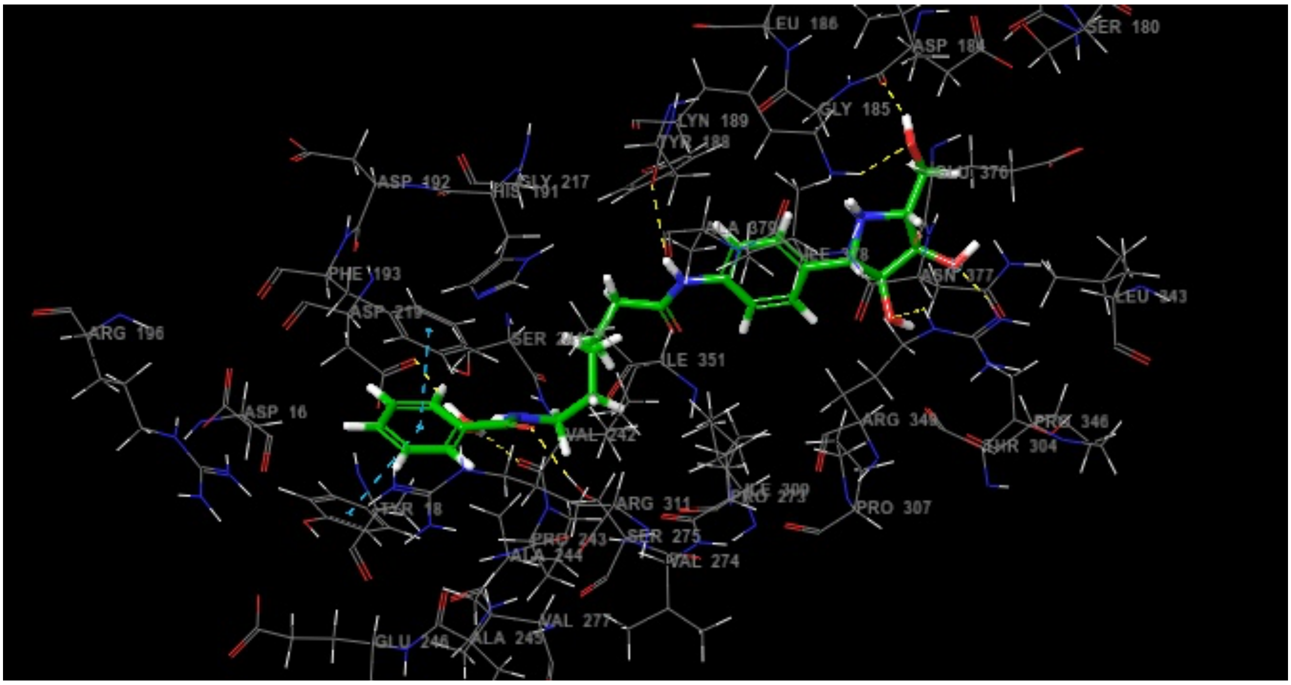

Compound 24

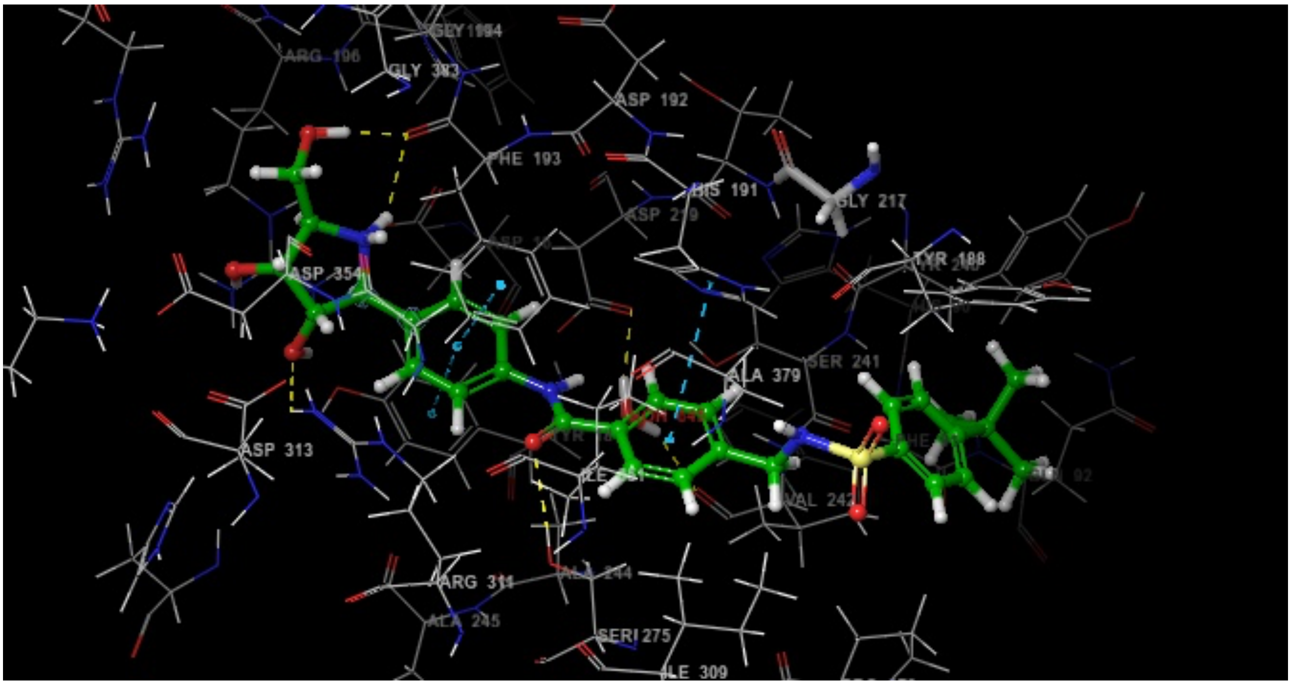

Compound 25

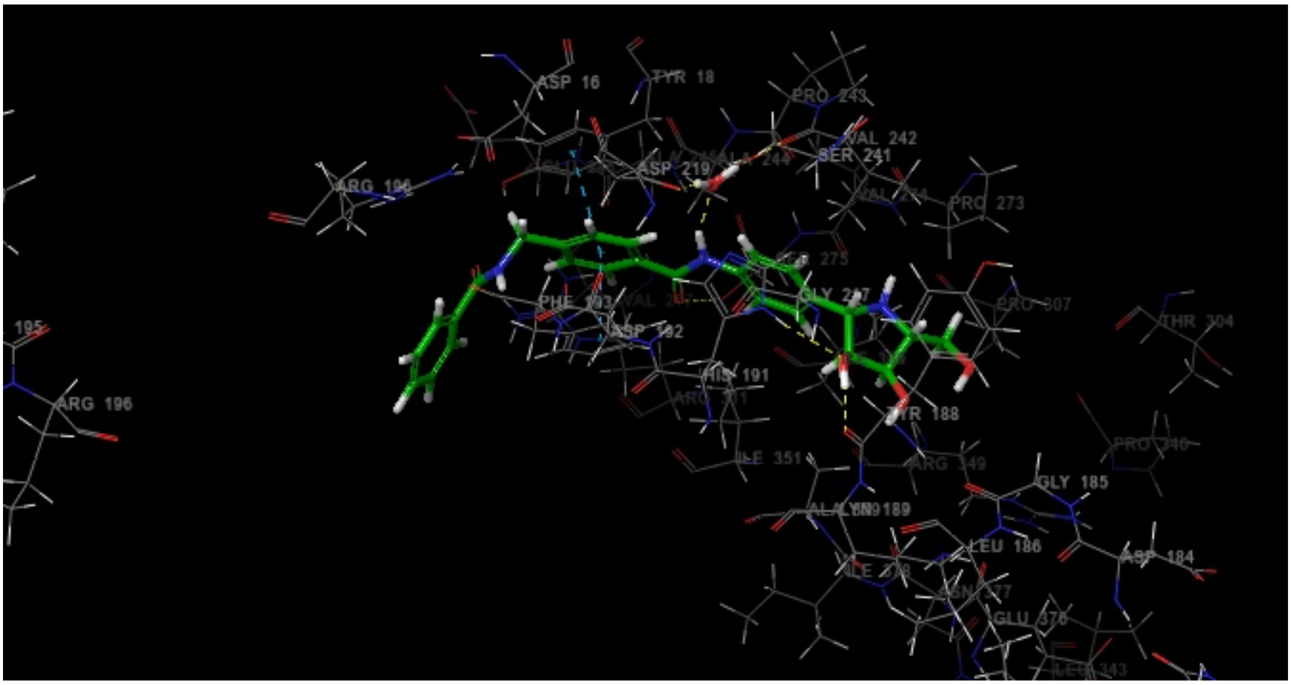

Compound 26

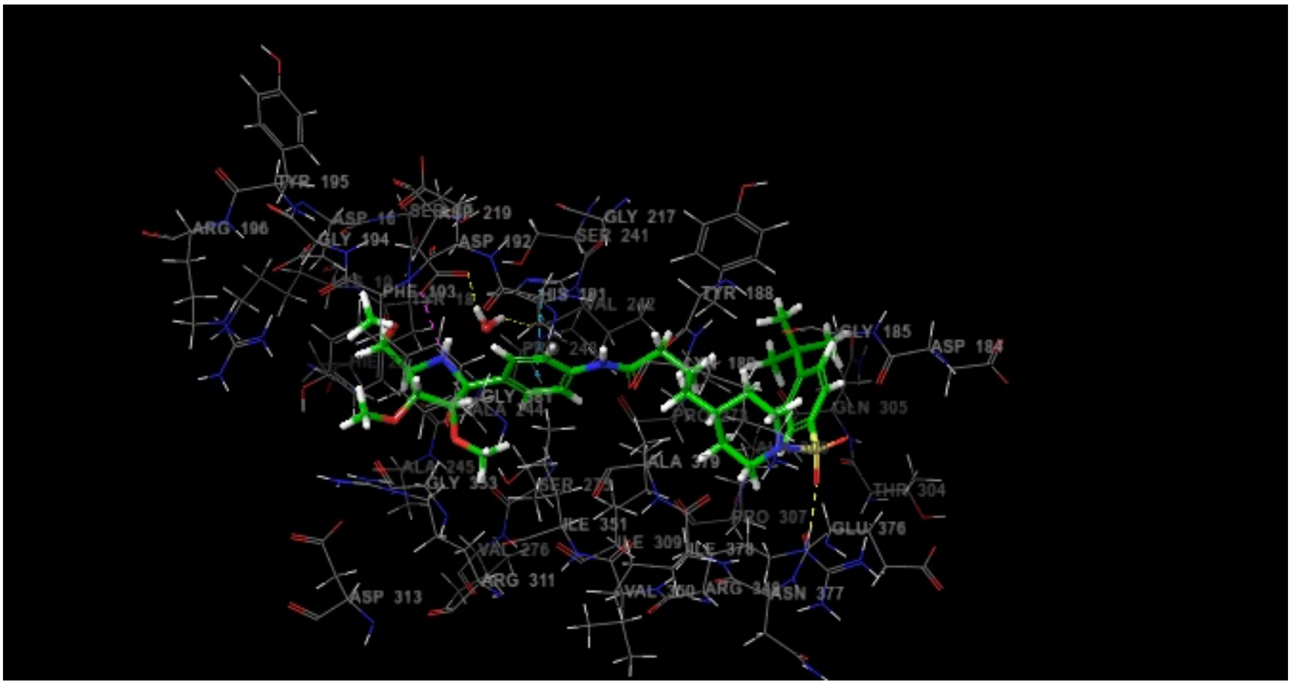

Compound 27

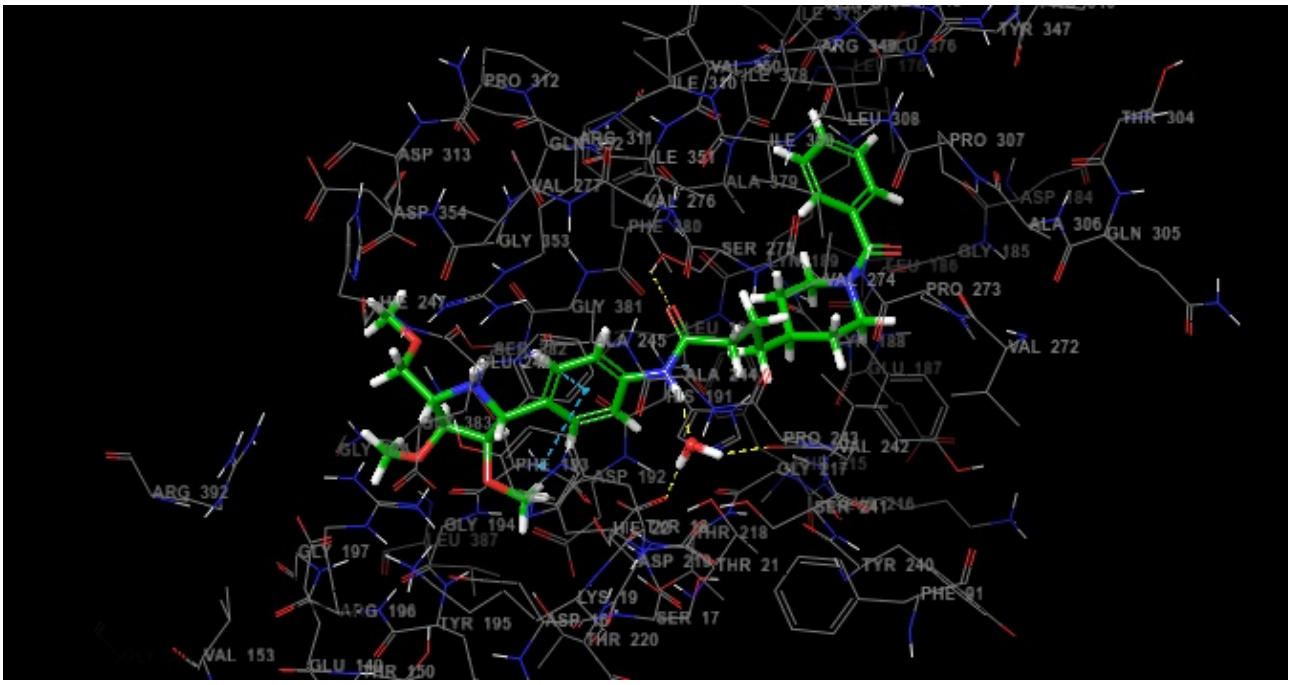

Compound 28

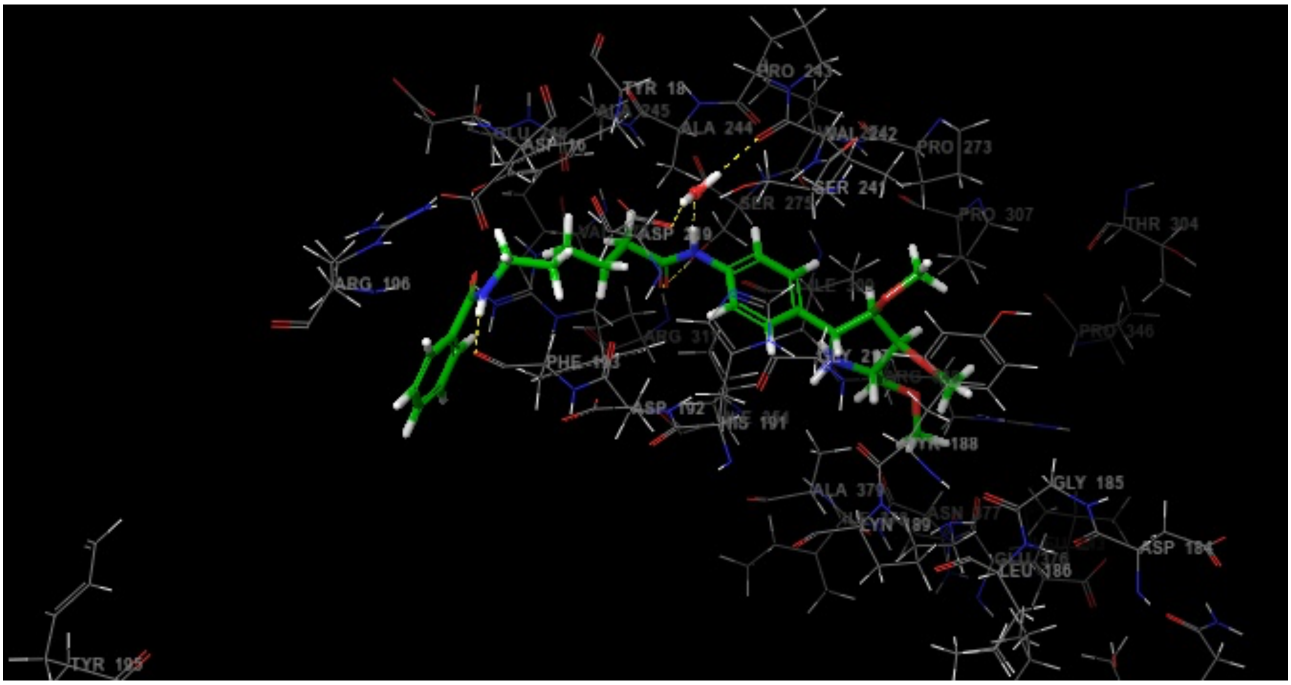

Compound 29

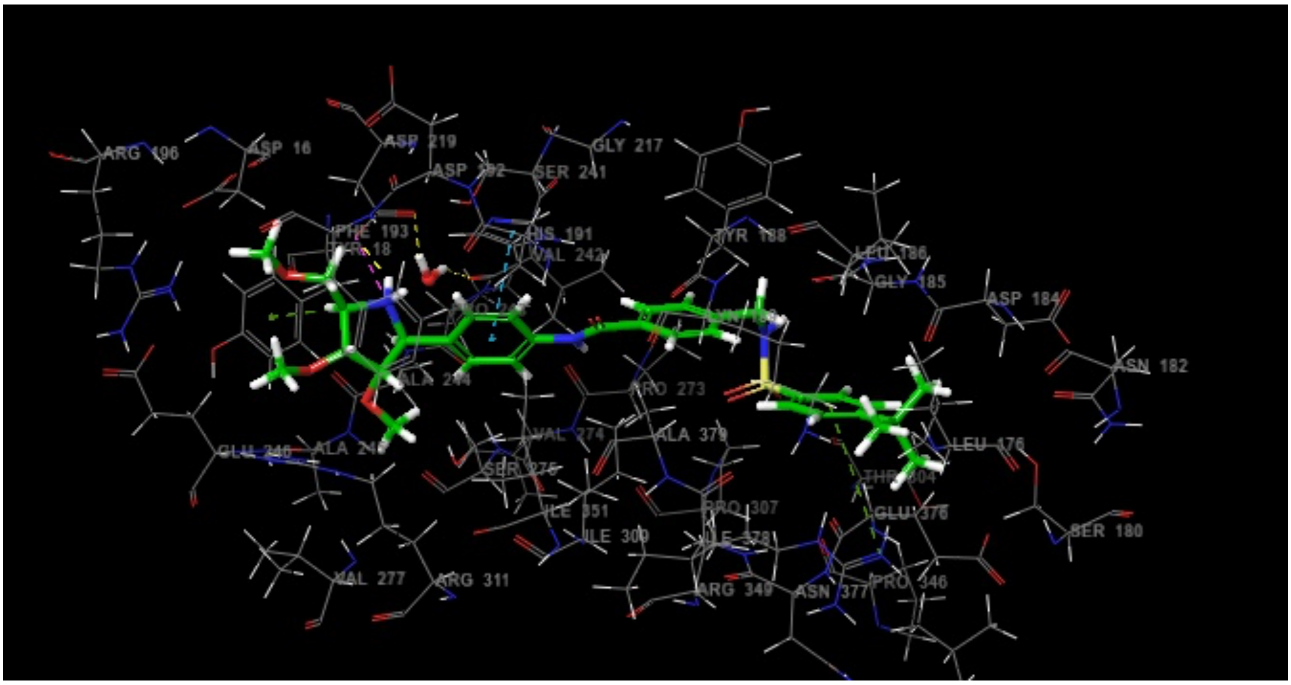

Compound 30

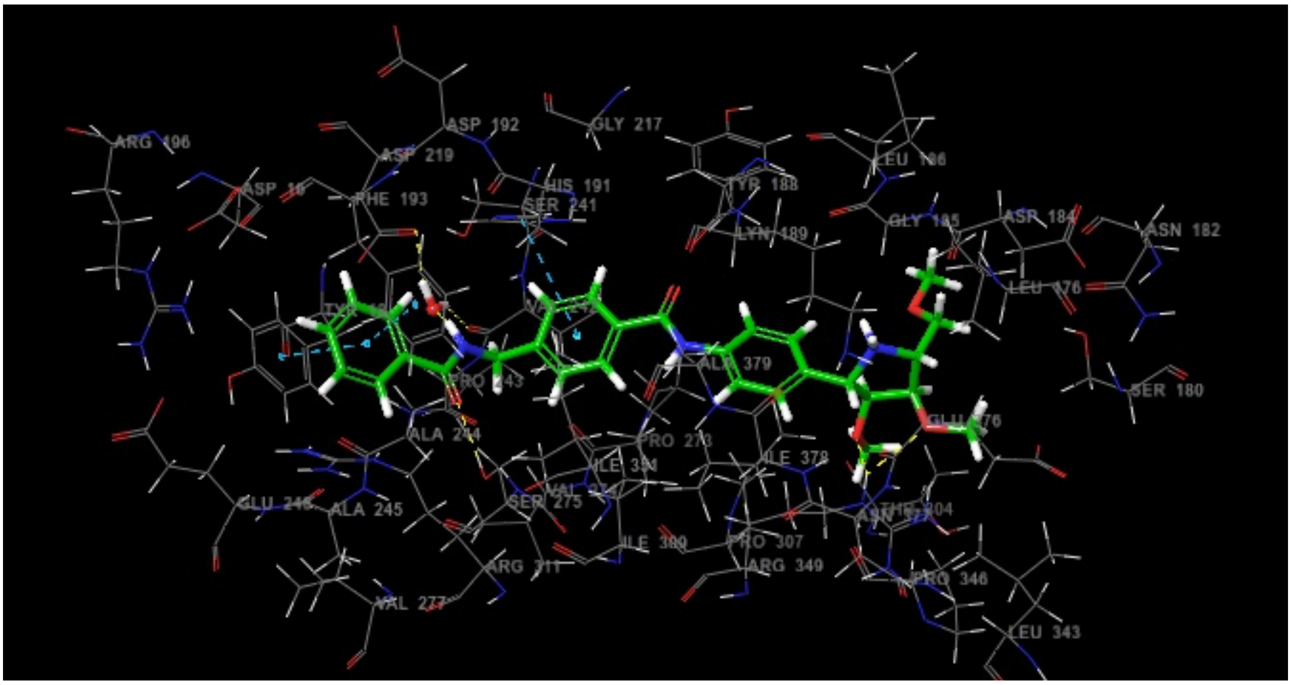

Supplement: Supplementary file 1 [file pharmaceutics-15-01472-s001.zip › pharmaceutics-2319585-supplementary.pdf]
